# Construction of circRNA‐miRNA‐mRNA network in the pathogenesis of recurrent implantation failure using integrated bioinformatics study

**DOI:** 10.1111/jcmm.16586

**Published:** 2021-05-07

**Authors:** Mohsen Ahmadi, Salar Pashangzadeh, Mahta Moraghebi, Soudabeh Sabetian, Mohammad Shekari, Fatemeh Eini, Ensieh Salehi, Pegah Mousavi

**Affiliations:** ^1^ Student Research Committee Hormozgan University of Medical Sciences Bandar Abbas Iran; ^2^ Department of Medical Genetics Faculty of Medicine Hormozgan University of Medical Sciences Bandar Abbas Iran; ^3^ Division of Medical Genetics Booali Medical Diagnostic Laboratory Qom Iran; ^4^ Iranian Research Center for HIV/AIDS Iranian Institute for Reduction of High‐Risk Behaviors Tehran University of Medical Sciences Tehran Iran; ^5^ Infertility Research Center Shiraz University of Medical Sciences Shiraz Iran; ^6^ Fertility and Infertility Research Center Hormozgan University of Medical Sciences Bandar Abbas Iran

**Keywords:** bioinformatic, circular RNA, endometrium, expression, GEO, immune, implantation failure, KEGG, microRNA, protein‐protein interaction

## Abstract

This research attempted to elucidate the molecular components are involved in the pathogenesis of recurrent implantation failure (RIF). We initially identified that 386 mRNAs, 144 miRNAs and 2548 circRNAs were differentially expressed (DE) in RIF and then investigated the genetic cause of the observed abnormal expression by constructing a circRNA‐miRNA‐mRNA network considering the competing endogenous RNA theory. We further analysed the upstream transcription factors and related kinases of DEmRNAs (DEMs) and demonstrated that SUZ12, AR, TP63, NANOG, and TCF3 were the top five TFs binding to these DEMs. Besides, protein‐protein interaction analysis disclosed that ACTB, CXCL10, PTGS2, CXCL12, GNG4, AGT, CXCL11, SST, PENK, and FOXM1 were the top 10 hub genes in the acquired network. Finally, we performed the functional enrichment analysis and found that arrhythmogenic right ventricular cardiomyopathy (ARVC), hypertrophic cardiomyopathy (HCM), pathways in cancer, TNF signalling pathway and steroid hormone biosynthesis were the potentially disrupted pathways in RIF patients. Optimistically, our findings may deepen our apprehensions about the underlying molecular and biological causes of RIF and provide vital clues for future laboratory and clinical experiments that will ultimately bring a better outcome for patients with RIF.

## 1 INTRODUCTION

Recurrent implantation failure (RIF) has been defined as an abnormal condition in which the transfer of more than ten good‐quality embryos frequently fails to implant following at least two cycles of in vitro fertilization (IVF) or intracytoplasmic sperm injection (ICSI).^1^, ^2^ Multiple aetiologies have been proposed for RIF occurrence including, advanced maternal age, increased body mass index, smoking status, stress levels, infectious disease, genetics and immunological factors.^3^ Plenty of literature has highlighted insufficient endometrial receptivity and constitutive endometrial dysfunction as a leading contributor to this condition.^4^ Moreover, many studies also have supported the idea that endometrial gene expression profiles may be altered in patients experiencing RIF.^5^ However, the exact mechanisms are not yet fully apprehended and need further elucidation. Therefore, it is crucial to investigate the possible genetic cause of abnormal endometrial gene expression profiles in RIF’s patients to find appropriate and effective targets for diagnostic and treatment purposes.

Non‐coding RNAs (ncRNAs) are a type of gene without any protein production that modulates cell function through many different mechanisms. Functionally, ncRNAs have been classified into two categories: housekeeping and regulatory ncRNAs. The first ones (eg tRNAs, rRNAs and snRNAs) play crucial roles in many cellular processes. The later ncRNAs (eg miRNAs, siRNAs and lncRNAs) are tissue‐specific and work in response to various internal and environmental stimuli.^6^ Among ncRNAs, circular RNAs (CircRNAs) are newly discovered and have a specific structure recognized by a covalently closed‐loop without 5’‐end caps, or 3’‐end poly (A) tails.^7^ Several research pieces have indicated that circRNAs exert their biological tasks by regulating transcription, maturation of RNAs, methylation of histones, and function as microRNA (miRNA) sponges.^8^ These shreds of evidence demonstrated the potential roles of circRNAs in physiological and pathological cellular conditions.^9^, ^10^ Interestingly, L Liu et al reported that the expressions of certain circRNAs were misregulated in RIF patients versus healthy controls.^11^ In 2011, Salmena et al presented the competing endogenous RNA (CeRNA) theory, in which ncRNAs control the expression of corresponding mRNAs by targeting the shared miRNAs through miRNA response elements.^12^, ^13^ Up to now, CeRNA networks have been constructed for many diseases and osteoarthritis,^14^ Diabetes Mellitus,^15^ coronary artery disease,^16^ lung cancer ^17^ and gastric cancer.^18^ Here, we assumed that the establishment of the circRNA‐miRNA‐mRNA network could help us to boost our apprehension about the involved processes in the RIF.

In this research, first, we gathered the expression profiles of mRNAs, miRNAs and circRNAs in RIF patients from the Gene Expression Omnibus (GEO) database. Next, using GEO2R analysis, the Differentially Expressed mRNAs (DEMs), miRNAs (DEMIs) and circRNAs (DECs) were determined. Furthermore, CircRNA‐miRNA and miRNA‐mRNA axes were established. Then, by integrating them, a circRNA‐miRNA‐mRNA network was established. Moreover, an assessment of the upstream transcription factor (TFs) and Protein Kinases (PKs) of DEMs was performed. Besides, commonly used enrichment analysis approaches such as protein‐protein interactions (PPI), Gene Ontology (GO) and Kyoto Encyclopedia of Genes and Genomes (KEGG) analyses were involved in the prediction of the probable mechanisms and tasks of dysregulated genes and pathways in RIF. The present work could advance our perception about the underlying molecular processes of RIF, providing novel diagnostic biomarkers, therapeutic targets and remarkable hints for further studies.

## 2 MATERIALS AND METHODS

### 2.1 Expression profile data

First, a total of three microarray expression profile data sets were selected from the National Center of Biotechnology Information (NCBI) GEO (https://www.ncbi.nlm.nih.gov/geo/) (Table 1). The GSE111974 data set was mRNA expression microarray data based on the GPL17077, Agilent‐039494 SurePrint G3 Human GE v2 8x60K Microarray 039 381 (Probe Name version), which included 48 samples (24 RIF samples and 24 fertile control patients’ samples). The GSE71332 data set contained the miRNA expression profile of seven RIF patients and five control endometria based on the GPL18402, Agilent‐046064 Unrestricted_Human_miRNA_V19.0_Microarray (miRNA ID version). The GSE147442 data set consisted of the circRNA expression profile of endometrium biopsies for eight RIF patients and eight fertile women based on the GPL21825, 074 301 Arraystar Human CircRNA microarray v2. All investigated samples in the microarray were obtained from Endometrial Tissue.

**TABLE 1:** Basic data of the three data sets involved in the present report

Profile	RNA type	Platform	Experiment type	Sample size (RIF/control)	Region	Year
GSE111974	mRNA	GPL17077	Expression profiling by array	24/24	Turkey	2018
GSE71332	miRNA	GPL18402	Non‐coding RNA profiling by array	7/5	China	2017
GSE147442	circRNA	GPL21825	Non‐coding RNA profiling by array	8/8	China	2020

### 2.2 Demonstration of DEGs

GEO2R (http://www.ncbi.nlm.nih.gov/geo/geo2r/), a web tool based on the R language limma package for comparing more than two groups of samples to obtain differentially expressed gene (DEGs) in almost any GEO series.^19^ We utilized this tool to conducting comparisons on GSE111974, GSE71332 and GSE147442 raw data. For this, we initially assessed the overall characteristics of value distributions. Usually, median‐centred values are representative that the data are normalized. If that was not our case, we used the force normalization option, which applies quantile normalization to the expression data forcing all selected samples to have identical value distribution. Then, we assigned samples from RIF patients and healthy women to ‘case group’ and ‘control group’, respectively. After that, we performed GEO2R analysis, and the results were downloaded and exported to Microsoft Excel 2019 software. Finally, we used a pre‐defined cut‐off criterion (*p*‐values <0.05 and |log Fold change (logFC)| > 1) and the Volcano Plot server (https://paolo.shinyapps.io/ShinyVolcanoPlot/), to identify DEGs in each data set and construct a volcano plot of DEGs.

### 2.3 Processing of DEGs

We used the miRNAmeConverter tool (http://163.172.134.150/miRNAmeConverter‐shiny/) ^20^ to annotate DEMIs names based on the last nomenclature released by the miRBase database (miRBase v.22).^21^ The terminology of DEMs and DECs also was converted according to the ‘Database for Annotation, Visualization, and Integrated Discovery’ (DAVID) v6.8 (https://david.ncifcrf.gov/conversion.jsp) and the circBase (http://www.circbase.org/cgi‐bin/webBlat) databases, respectively.^22^, ^23^, ^24^ When we could not find a known gene for a particular DEG, we eliminated it for further investigations. The DEGs of GSE111974 annotated as any type of gene such as lncRNA, pseudogenes and uncharacterized genes except mRNA (protein‐coding) also were ruled out. We converted the multiple expression values referred to the same gene to a single value using the following approach: Genes with the same direction of expression were averaged and discarded if they had the reversed expression tendency.

### 2.4 Assessment of the reliability of GEO data sets

In order to check the reliability of GEO data sets included in our research, we investigated the expression levels of several proposed marker genes of RIF among obtained DEGs including, TP53, VEGF, COX2, LIF and MUC‐1 ^3^, ^25^, ^26^ in GSE111974; miR‐20b, miR‐155, miR‐718, miR‐99a, miR‐145, miR‐23b and miR‐29c ^27^, ^28^, ^29^ in GSE71332; and hsa_circ_0070617, hsa_circ_0023461, hsa_circ_0038383, hsa_circ_0000115 and hsa_circ_0091053 ^30^, ^31^ in GSE147442.

### 2.5 Construction of the miRNA‐mRNA network

The miRDIP v4.1 online tool (http://ophid.utoronto.ca/mirDIP/), as an integrated database of human microRNA‐target predictions across from 30 independent resources with an integrative score, was used to identify the possible interactions between DEMs and DEMIs via bidirectional mode search under a high confidence filter (score class 5% = high).^32^ It hastwo major approaches for computational prediction of the appropriate miRNA‐mRNA pairs: identification of downstream gene targets for selected miRNA or upstream miRNA regulators for query gene. The second is to implement validation of the miRNA‐gene correlation analysis using previous experiments. As miRNAs tend to control the expression of their desired mRNAs, even to a small extent, only the pair of miRNA‐mRNAs with reverse expression patterns were included for miRNA‐mRNA network construction.^33^, ^34^

### 2.6 Establishment of the circRNA‐miRNA network

Targeting circRNAs of DEMIs in miRNA‐mRNA network was predicted through the circBank (http://www.circbank.cn/) database.^35^ Then, we screened these circRNAs by DECs in GSE147442. As the expression levels of circRNAs typically do not alter the expression of corresponding miRNAs,^36^ the circRNA‐miRNA pairs were selected without the restriction that the correlation between circRNAs and miRNAs must be negative.

### 2.7 Reconstruction of circRNA‐miRNA‐mRNA network

We combined the pairs of circRNA‐miRNA and miRNA‐mRNA to reconstruct the circRNA‐miRNA‐mRNA network. The nodes that could not reach a circRNA‐miRNA‐mRNA axis were eliminated. The circRNA‐miRNA‐mRNA network was visualized utilizing Cytoscape 3.8.0.^37^

### 2.8 TFs and PKs related to DEGs

The upstream regulators and protein kinases related to 386 DEMs in GSE111974 were recognized by submitting their official gene names to Expression2Kinases (X2K) tool (https://amp.pharm.mssm.edu/X2K/).^38^ The top 10 most enriched TFs and PKs were listed regarding the integrated (p‐value and z‐score) score.

### 2.9 PPI network analysis

Based on the 386 DEMs in GSE111974, the Search Tool for the Retrieval of Interacting Genes/Proteins (STRING) database (https://string‐db.org/) ^39^ was employed to perform the PPI network analysis. Nodes that were not connected to the central network and PPI pairs with an integrated score <0.4 were removed to acquire more reliable results. The PPI network was displayed using Cytoscape 3.8.0. The cytoHubba app in Cytoscape was used to disclose the top ten hub genes in the PPI network.^40^

### 2.10 Functional enrichment analysis

The DAVID v6.8 database was applied to execute the GO and KEGG pathway analysis on 386 DEMs in GSE111974. The GO items involved in this study were as follows: molecular function (MF), cellular component (CC) and biological process (BP). The retrieved items were imported to Microsoft Excel 2019, and after removing those terms with a p‐value greater than 0.05, they were ranked based on the p‐value parameter. The bar chart was visualized utilizing Microsoft Excel 2019.

## 3 RESULTS

### 3.1 DEMs, DEMIs and DECs in RIF

**FIGURE 1:**
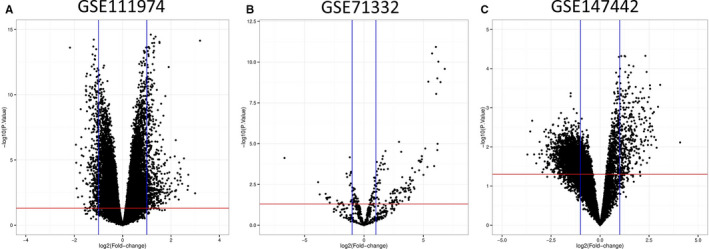
Volcano plot shows the DEMs in GSE111974(A), DEMIs in GSE71332(B) and DECs in GSE147442(C). DEMs: differentially expressed mRNAs; DEMIs: differentially expressed miRNAs [Colour figure can be viewed at wileyonlinelibrary.com]

From the GEO database and employing the GEO2R, we first collected 50 599, 2027 and 12 625 different probe IDs from GSE111974, GSE71332 and GSE147442, respectively. With the pre‐defined threshold (*P* <.05 and |logFC|>1) and using the Volcano Plot server and processing the acquired DEGs, we then obtained a total of 386 DEMs (Table S1), comprising 214 over‐expressed and 172 lower‐expressed mRNAs were recognized between RIF and control patients from GSE111974 (Figure 1A). Furthermore, 144 DEMIs (Table S2) were extracted from GSE71332, containing 113 up‐regulated and 31 down‐regulated miRNAs (Figure 1B). Besides, 2548 DECs (Table S3) were generated from GSE147442. Among them, the expressions of 340 circRNAs were elevated, and 2208 circRNAs undergo down‐regulation (Figure 1C).

### 3.2 Data set validation

To assess the robustness of data sets, we evaluated the expression profile of suggested marker genes of RIF between retrieved DEGs and found that LIF (logFC = −1.353, *P* = 1.77E‐02) was down‐regulated and MUC‐1 (logFC =1.686, *P* = 4.02E‐09) and COX2 (logFC =1.401, *P* = 3.39E‐04) were up‐regulated in GSE111974 (Table S1). Moreover, we observed that hsa‐miR‐99a‐3p (logFC =2.209, *P* = 4.46E‐02), hsa‐miR‐145‐5p (logFC =1.678, *P* = 3.84E‐04), hsa‐miR‐145‐3p (logFC =4.818, *P* = 1.27E‐04) and hsa‐miR‐29c‐5p (logFC =3.286, *P* = 6.62E‐03) were overexpressed in GSE71332 (Table S2). Among those circRNAs that been have proposed for RIF, the expression levels of hsa_circ_0038383 (logFC = −2.508, *P* = 4.00E‐03), hsa_circ_0000115 (logFC = −2.769, *P* = 4.00E‐03) and hsa_circ_0091053 (logFC = −2.101, *P* = 2.00E‐03) were decreased in GSE147442 (Table S3).

### 3.3 Searching for a potential connection between DEMs, DEMIs and DECs

We studied the possible regulatory relationship between DEMIs in GSE71332 and DEMs in GSE111974 through the miRDIP v4.1 database. As depicted in Figure S1, the results presented 966 miRNA‐mRNA pairs in which 87 DEMIs presumably sponged 170 DEMs. Then, we predicted targeting circRNAs of 87 DEMIs in the miRNA‐mRNA network utilizing the circBank database. By overlapping these circRNAs with DECs in GSE147442, we got 381 circRNAs‐miRNAs pairs (Figure S2), including 180 DECs and 77 DEMIs.

### 3.4 Construction of the circRNA‐miRNA‐mRNA network

**FIGURE 2:**
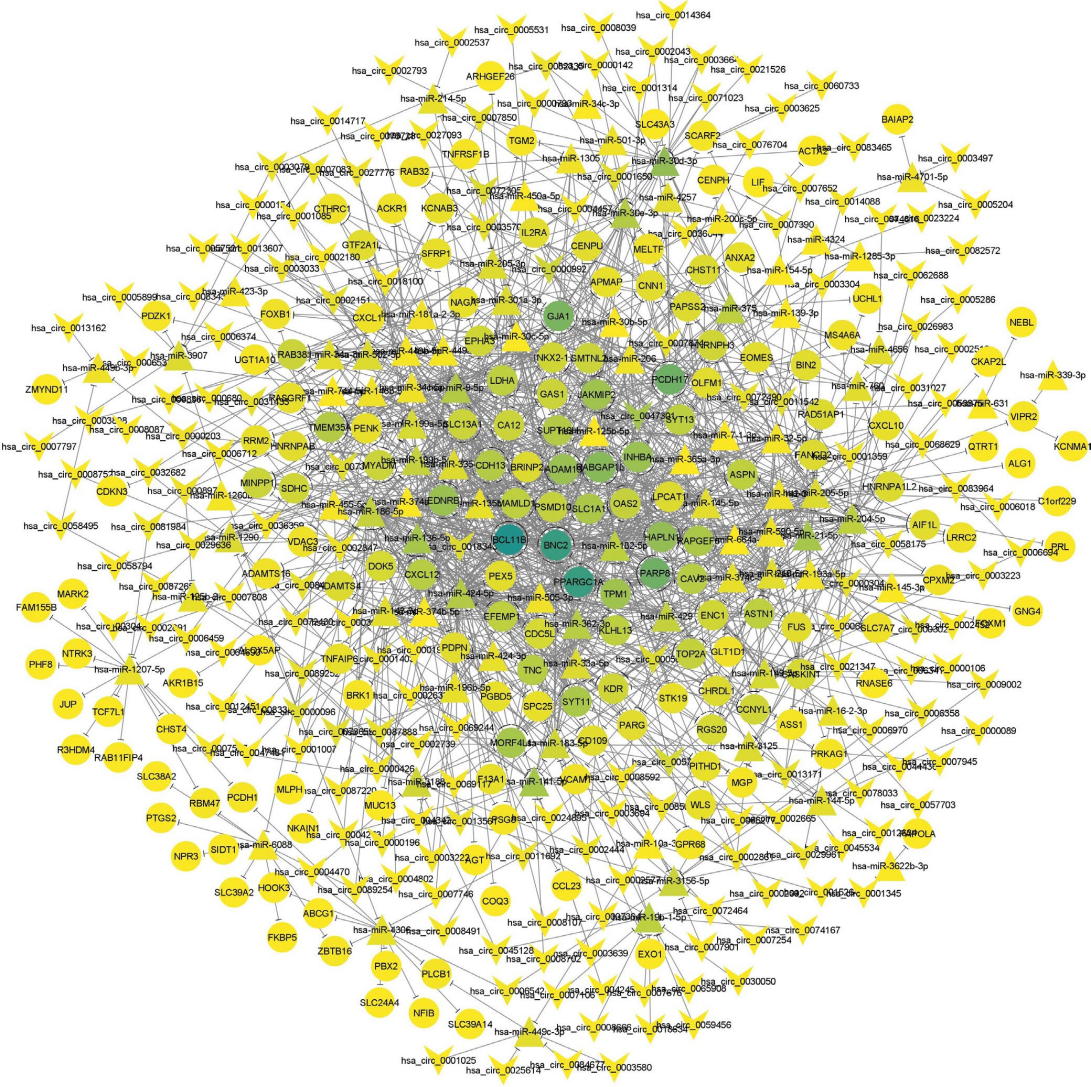
The CeRNA network of circRNA‐miRNA‐mRNA interactions in recurrent implantation failure. V shape indicates circRNAs, triangle indicates miRNAs and round rectangle indicates mRNAs. CeRNA: competitive endogenous RNA [Colour figure can be viewed at wileyonlinelibrary.com]

We established a circRNA‐miRNA‐mRNA network by merging the circRNAs‐miRNAs and miRNA‐mRNA pairs and excluding the nodes that were not linked to the central network. As exhibited in Figure 2, the final network was comprised of 437 nodes and 1375 edges.

### 3.5 Analysis of upstream regulator of DEMs

**FIGURE 3:**
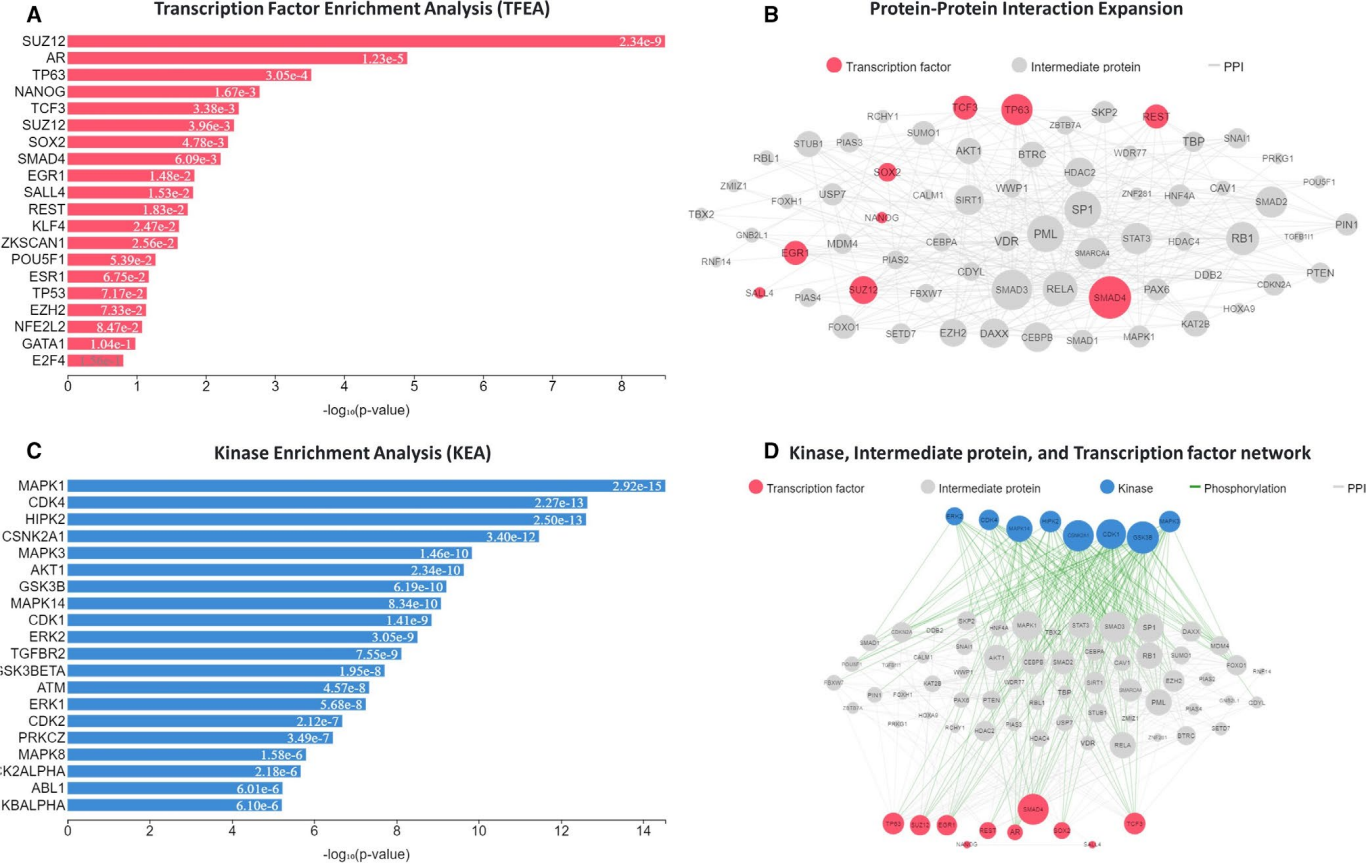
The TFs, mediatory protein and PKs networks. Expression2Kinases investigations signified the most enriched TFs, mediatory protein and kinase from the 386 DEMs in GSE111974 according to the integrated score (p‐value and z‐score). TFs, transpiration factors; PKs, protein kinases; DEMs, differentially expressed mRNAs [Colour figure can be viewed at wileyonlinelibrary.com]

We recognized the enriched upstream TF, mediatory protein, and related PKs to elucidate the molecular processes of 386 DEMs in GSE111974. The X2K approach unveiled that Suz12 Polycomb Repressive Complex 2 Subunit (SUZ12), Androgen Receptor (AR), Tumor Protein P63 (TP63), Nanog Homeobox (NANOG) and Transcription Factor 3 (TCF3) were the top five TFs binding to DEMs (Figure 3A). We also found that these TFs were related to 58 intermediate proteins (Figure 3B). Our investigations then uncovered the prominent protein kinases such as mitogen‐activated protein kinase 1 (MAPK1), cyclin‐dependent kinase 4 (CDK4), homeodomain‐interacting protein kinase 2 (HIPK2), Casein Kinase 2 Alpha 1 (CSNK2A1) and mitogen‐activated protein kinase 3 (MAPK3) for these DEMs (Figure 3C) had relations with a plenty number of mediatory proteins and TFs (Figure 3D).

### 3.6 Identifying hub genes in PPI network construction

**FIGURE 4:**
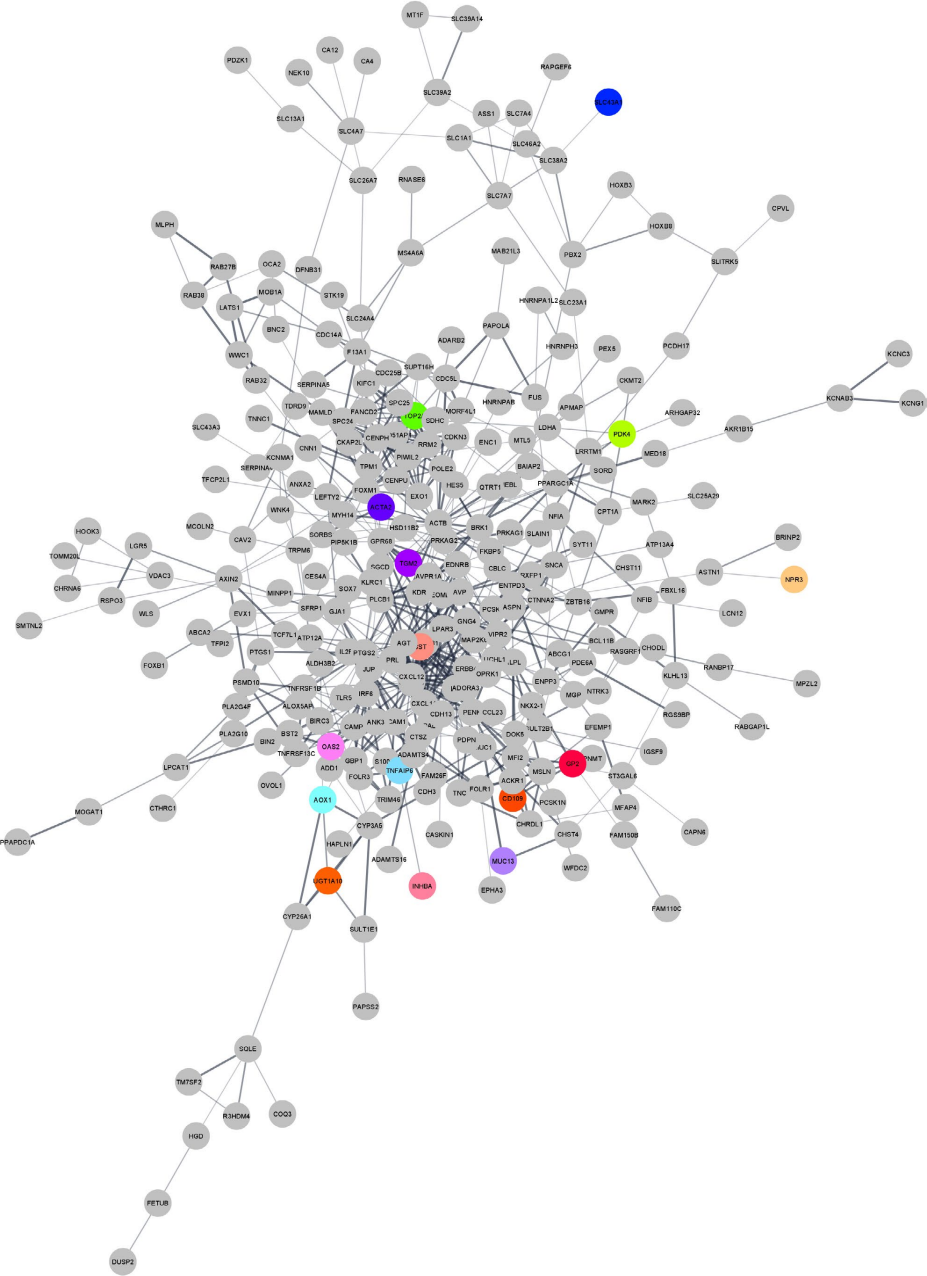
The PPIs networks. The PPIs of 386 DEMs in GSE111974 were established using the STRING database. DEMs, differentially expressed mRNAs; PPI, protein‐protein interaction [Colour figure can be viewed at wileyonlinelibrary.com]

PPI networks were constructed using the STRING database. The PPI network showed 279 nodes and 667 edges (Figure 4). The top 10 identified hub genes were ranked as follows (Table 2): Actin Beta (ACTB), C‐X‐C Motif Chemokine Ligand 10 (CXCL10), Prostaglandin‐Endoperoxide Synthase 2 (PTGS2), C‐X‐C Motif Chemokine Ligand 12 (CXCL12), G Protein Subunit Gamma 4 (GNG4), Angiotensinogen (AGT), C‐X‐C Motif Chemokine Ligand 11 (CXCL11), Somatostatin (SST), Proenkephalin (PENK) and Forkhead Box M1 (FOXM1).

**TABLE 2:** The rank of top ten hub genes through degree parameter

Genes	Degree
ACTB	28
CXCL10	25
PTGS2	25
CXCL12	23
GNG4	22
AGT	21
CXCL11	18
SST	18
PENK	17
FOXM1	17

### 3.7 Enrichr database analysis

**FIGURE 5:**
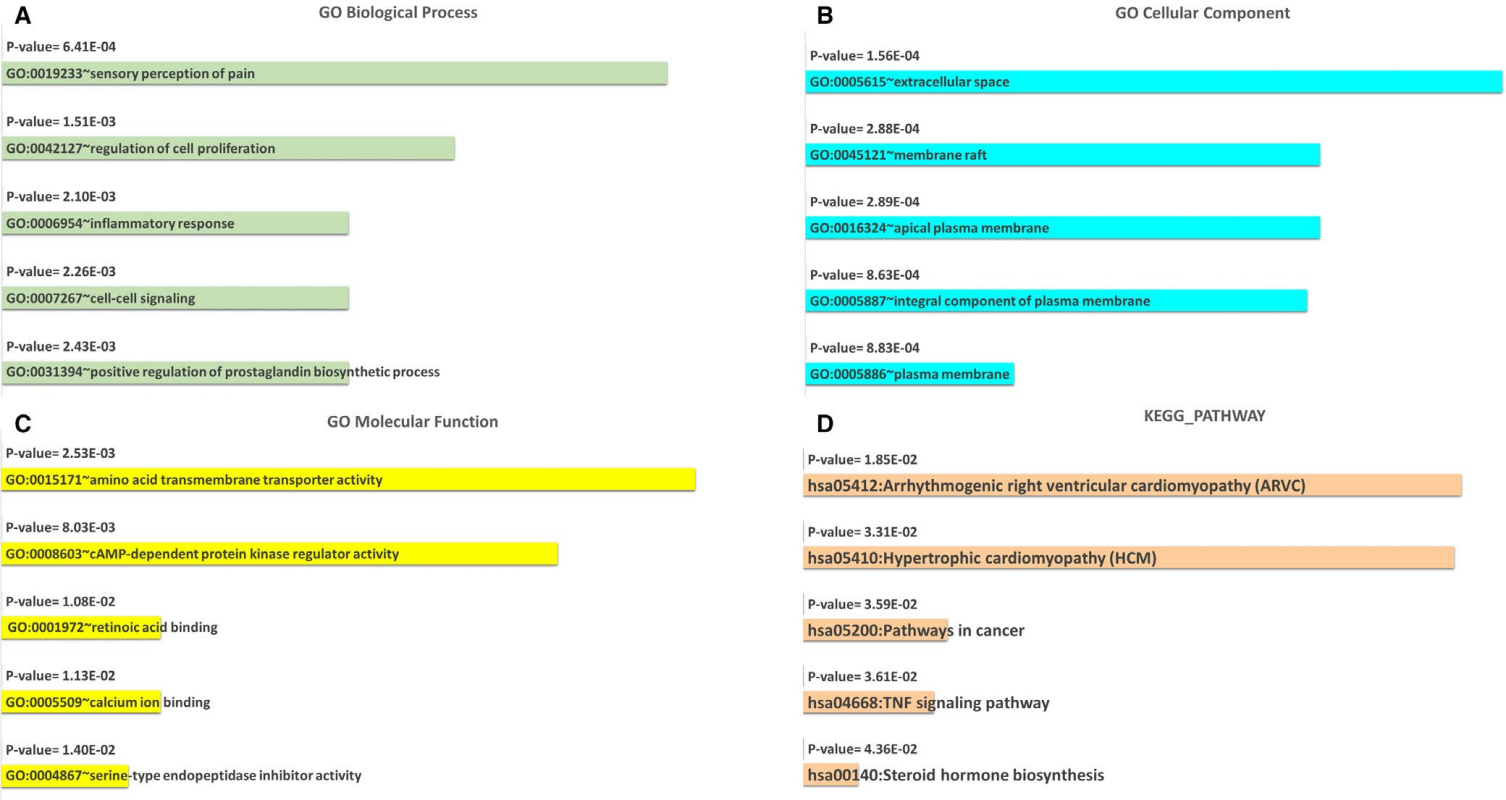
The functional enrichment analysis of 386 DEMs in GSE111974. The top 10 enriched GO (A) BP, (B) CC, (C) MF terms and (D) KEGG pathways. DEMs, differentially expressed mRNAs; GO, gene ontology; KEGG, Kyoto encyclopedia of genes and genomes; BP, biological process; CC, cellular component; MF, molecular function [Colour figure can be viewed at wileyonlinelibrary.com]

The DAVID v6.8 database was utilized for functional enrichment analysis based on 386 DEMs in GSE111974. For GO analysis, when considering BPs, these DEMs were enriched in sensory perception of pain (GO:0 019 233), regulation of cell proliferation (GO:0 042 127), inflammatory response (GO:0 006 954), cell‐cell signalling (GO:0 007 267) and positive regulation of prostaglandin biosynthetic process (GO:0 031 394). With regard to CC, the top five enriched items were extracellular space (GO:0 005 615), membrane raft (GO:0 045 121), apical plasma membrane (GO:0 016 324), an integral component of the plasma membrane (GO:0 005 887) and plasma membrane (GO:0 005 886). In terms of MF, amino acid transmembrane transporter activity (GO:0 015 171), cAMP‐dependent protein kinase regulator activity (GO:0 008 603), retinoic acid binding (GO:0 001 972), calcium ion binding (GO:0 005 509) and serine‐type endopeptidase inhibitor activity (GO:0 004 867) were enriched for the first five places. Pathway enrichment analysis disclosed that the top five predominantly enriched pathways were hsa05412:Arrhythmogenic right ventricular cardiomyopathy (ARVC), hsa05410:Hypertrophic cardiomyopathy (HCM), hsa05200:Pathways in cancer, hsa04668:TNF signalling pathway and hsa00140:Steroid hormone biosynthesis (Figure 5A‐D).

## 4 DISCUSSION

The current study intended to seek remarkable aspects for the diagnosis, treatment of patients with RIF through analysing the gene expression.

In this report, we first demonstrated 386 DEMs from GSE111974, 144 DEMIs from GSE71332 and 2548 DECs from GSE147442. We believe that the altered expression of these genes could have vital roles in the progression of RIF. In accordance with our idea, previously, several studies have attempted to compare the expression of genes in endometrium tissue or peripheral blood taken from patients with RIF with women who experienced successful implantation. For instance, Bastu et al recognized the dysregulation of 641 genes in patients with RIF comparing to healthy women.^41^ Other scientists suggested that a differential expression of 303 genes in the endometrium biopsies of women who underwent IVF could anticipate the event of RIF.^42^ Multiple causes have been introduced for the dysregulation of genes, including miRNAs' aberrant expression.^43^ Furthermore, dysregulation of miRNAs has been proposed to have a duty to modulate embryo implantation.^44^ Revel et al demonstrated the up‐regulation of miR‐99a, miR‐145 and miR‐23b in the endometrial tissues of patients suffering from RIF.^27^ The up‐regulation of miR‐145 was also indicated in women with RIF by others..^45^ Furthermore, Huang et al revealed that during embryo implantation, the increased expression of miR‐23a‐3p enhances the receptivity of the endometrium by regulating the expression of CUL3.^46^ Others proved that the inhibition of PAI‐1 via miR‐145 might take part in the RIF occurrence.^47^ Another report by Griffiths et al discovered that the overexpressed miR‐29c potentially have a role in the progression of implantation failure and infertility through reducing the expression of COL4A1 and disruption of endometrial adhesive capacity.^28^ Along with miRNA, CircRNAs, as a novel group of non‐coding RNAs, also mediate the gene expression levels, and their abnormal expression has been shown to participate in diverse disorders containing RIF.^48^ Liu et al found 856 DECs in the endometrial samples of six RIF patients and control group.^11^

Based on the CeRNA hypothesis, we sought to obtain the potential regulatory between the retrieved differently expressed genes. Therefore, we investigated the possible connections between the miRNAs and DEMs and established 966 miRNA‐mRNA pairs in which 87 DEMIs may modulate the expression of 170 DEMs. Subsequently, we identified the upstream targeting circRNAs of 87 DEMIs in the miRNA‐mRNA network. By intersecting these circRNAs with DECs in GSE147442, 381 circRNAs‐miRNAs pairs were constructed. Next, by merging the circRNAs‐miRNAs and miRNA‐mRNA pairs and removing the nodes without any connection to the central network, we created a circRNA‐miRNA‐mRNA regulatory network in RIF with 437 nodes and 1375 edges.

Next, we disclosed that SUZ12, AR, TP63, NANOG and TCF3 were the top five TFs binding to DEMs in GSE111974. The 58 recognized intermediate proteins might take part in the mechanism of actions of TFs in the RIF. We also showed that retrieved protein kinases for DEMs, including MAPK1, CDK4, HIPK2, CSNK2A1 and MAPK3, were associated with the numerous intermediate proteins and TFs. We thought that these TFs and their downstream target genes may regulate RIF progression and could be further investigated as a potential target for the diagnosis or therapeutic applications.

Besides, we imported the list of 386 DEMs in GSE111974to the STRING database to depict the PPI network. The PPI network is comprised of 667 edges and 279 nodes. The additional analyses told us that ACTB, CXCL10, PTGS2, CXCL12, GNG4, AGT, CXCL11, SST, PENK and FOXM1 were the top 10 hub genes in the established PPI network. When we focused on the expression of these hub genes, we realized that only ACTB and PTGS2 were overexpressed in the endometrium samples of RIF patients. The ACTB is a protein‐coding gene that encodes cytoplasmic b‐actin, which incorporates cellular and biological functions such as cell integrity and intercellular signalling pathways.^49^ Currently, a report published by Cuvertino et al have proposed that a significant decrease in the amount of β‐actin may impair the development of the brain, heart and kidney.^50^ Moreover, certain pathogenic variants in the ACTB gene have been reported in a developmental disorder called the Baraitser‐Winter syndrome.^51^ Besides, it has shown that the ACTB gene variants can increases the susceptibility of the Han Chinese population to diabetic kidney disease.^52^ CXCL10, CXCL11 and CXCL12 are members of the CXC (cys‐x‐cys) subgroup of cytokines. CXCL10 and CXCL11 and their corresponding receptor CXCR3 are included in the mediation of several processes such as apoptosis, cell growth and chemotaxis. The expression levels of CXCL10 and CXCL11 are usually low in homeostatic conditions but can elevate through cytokine stimulation. The association of CXCL10 and CXCL11 with diverse human conditions like immune dysfunction and tumour progression has been documented. After implantation, decidual stromal cells down‐regulate CXCL10 to modify dynamic immunological changes that are a prerequisite for the maternal‐foetal interface, a phenomenon that highlights the importance of CXCL10 in a successful pregnancy.^53^, ^54^ CXCL12, as another constituent of the CXC subfamily, was first realized in the stromal cells of bone marrow. CXCL12 and its receptor CXCR4 are widely identified at the maternal‐foetal interface and are proposed to have a substantial task in the implantation of embryo and successful pregnancy. Several researchers have discovered that CXCL12 reduces apoptosis and elevates the viability of trophoblast cells in culture media. It also has suggested that the CXCL12/CXCR4/CXCR7 axis creates an environment that allows the development of the embryo and contributes to immune tolerance.^55^, ^56^ In addition, The diminished expression of CXCL12 in the endometrium tissues been have confirmed to cause the lessened endometrial function.^57^ Noteworthy, Koo et al showed that pre‐ and peri‐implanting embryos‐derived CXCL12 and its connection with CXCR4 and CXCR7 increase the receptivity of the endometrium and promote angiogenesis. They also indicated that the intra‐uterine application of CXCL12 should be considered as a non‐invasive therapeutic option for treatment RIF patients.^58^ PTGS2, which is also noticed as cyclooxygenase‐2 (COX‐2), is an essential enzyme in prostaglandins' biosynthesis. It has well accepted that the prostaglandins' synthesis might be a central element in the established development of endometrial receptivity. Considerably, the compromised levels of prostaglandins and COX‐2 were noticed in the majority of patients with RIF.^59^ GNG4 is a regulator and transducer of various transmembrane signalling systems and takes part in haemostasis and glucagon response. Specific variants in GNG4 have been correlated with cognitive impairment.^60^ AGT is a protein‐coding gene involved in sustaining blood pressure, homeostasis of electrolytes and body fluid. Genetic variations in the AGT gene have been related to hypertension, endometrial cancer, pre‐eclampsia and spontaneous abortion.^61^, ^62^ SST is a cyclic peptide hormone involved in the secretion of growth hormone, quiescence of stem cells and cancer development.^63^ Zaino et al have demonstrated the up‐regulation of SST in poorly differentiated endometrial cancer cells.^64^ Zou et al also have indicated that the significant overexpression of SST may have roles in the development and progression of endometrial cancer.^65^ PENK is the protein‐coding gene that participates in cellular signalling pathways such as GPCR and NF‐KB. Beunders et al have introduced PENK as a useful biological marker for the assessment of kidney function.^66^ FOXM1, as a transcription factor from the Forkhead family, is engaged in the apoptosis and cell cycle initiation and adjusts the developmental function of diverse organs in the human body.^67^ It has been determined that foxM1 is up‐regulated in endometrial cancer.^68^ Moreover, Xie et al have shown that FOXM1 is a key modulator of endometrial receptivity.^69^

Finally, we carried out the functional enrichment analysis for 386 DEMs in GSE111974. GO analysis revealed that these DEMs were engaged in biological activities such as regulation of cell proliferation, inflammatory response, cell‐cell signalling and positive regulation of the prostaglandin biosynthetic process. They were prominent in extracellular space, membrane raft, apical plasma membrane, an integral component of the plasma membrane and the plasma membrane at the cellular level. When we considered the MF analysis, the results revealed that these DEMs were mainly involved in amino acid transmembrane transporter activity, cAMP‐dependent protein kinase regulator activity, retinoic acid binding, calcium ion binding and serine‐type endopeptidase inhibitor activity. Pathway enrichment assessments determined that the top five enriched pathways were arrhythmogenic right ventricular cardiomyopathy (ARVC), hypertrophic cardiomyopathy (HCM), pathways in cancer, TNF signalling pathway and steroid hormone biosynthesis. We speculated that dysregulated genes are likely exerting their roles in RIF progression through these potential pathways.

To the best of our knowledge, the present research was the first attempt to build a circRNA‐miRNA‐mRNA network and identify the most potential corresponding pathways in the pathogenesis of RIF. However, our study had some limitations. Firstly, due to the limited number of GEO data sets, we only have utilized the data obtained from three data sets deposited in the GEO database. Additionally, the sample size of these data sets was few. Therefore, it was hard to prevent random errors. Besides, our analyses' findings were not confirmed with reliable laboratory analysis, and we just focused on in‐silico assessments. Depending solely on bioinformatics investigations may trigger deviations. Hence, in vitro experiments remain to be carried out to validate the results of the present report.

## 5 CONCLUSION

In conclusion, the present research performed various bioinformatics analyses on the DEGs in the endometrium of RIF patients. These investigations demonstrated that the hub genes in RIF directly or indirectly affect cell cycle, apoptosis, angiogenesis and immune response‐related pathways that modulate the endometrial receptivity and embryo implantation. Hopefully, our study's findings may bear an increased knowledge about the underlying molecular and biological aetiologies of RIF and valuable clues for additional experiments that will ultimately lead to improved capacity for accelerated and accurate diagnosis and treatment of patients with RIF.

## CONFLICT OF INTEREST

The authors declare no competing interests.

## AUTHOR CONTRIBUTIONS

**Mohsen Ahmadi:** Conceptualization (equal); Formal analysis (equal); Investigation (equal); Methodology (equal); Visualization (equal); Writing‐original draft (equal); Writing‐review & editing (equal). **Salar Pashangzadeh:** Writing‐original draft (equal); Writing‐review & editing (equal). **Mahta Moraghebi:** Conceptualization (equal); Formal analysis (equal); Methodology (equal); Visualization (equal). **soudabeh Sabetian:** Project administration (equal); Supervision (equal); Validation (equal); Writing‐review & editing (equal). **mohammad shekari:** Writing‐original draft (equal). **Fatemeh Eini:** Writing‐original draft (equal). **Ensieh Salehi:** Writing‐original draft (equal). **Pegah Mousavi:** Formal analysis (equal); Funding acquisition (equal); Methodology (equal); Project administration (equal); Validation (equal); Writing‐original draft (equal); Writing‐review & editing (equal).

## Supporting information

Supporting Information

## Data Availability

All data generated\examined for the present research are presented in the manuscript context and the supplementary file.
