# Supplementary material for: Construction of circRNA‐miRNA‐mRNA network in the pathogenesis of recurrent implantation failure using integrated bioinformatics study
Source: J Cell Mol Med. 2021 May 7;26(6):1853–64. doi: 10.1111/jcmm.16586 (PMC8918409; doi:10.1111/jcmm.16586)
Supplement: Supplementary file 1 — Supporting Information [file JCMM-26-1853-s001.docx]

**Supplementary table 1. The** **significantly upregulated and downregulated mRNAs in the GSE111974 dataset.**

|  | mRNA_ID | logFC | P-value | Expression |
| --- | --- | --- | --- | --- |
| 1 | PHF8 | 3.197 | 7.18E-15 | Up-regulated |
| 2 | CYP26A1 | 2.996 | 3.56E-03 | Up-regulated |
| 3 | SLC7A4 | 2.711 | 1.36E-04 | Up-regulated |
| 4 | CAPN6 | 2.703 | 9.79E-04 | Up-regulated |
| 5 | TNNC1 | 2.393 | 4.66E-05 | Up-regulated |
| 6 | PNMT | 2.335 | 1.59E-05 | Up-regulated |
| 7 | CHST4 | 2.266 | 3.70E-04 | Up-regulated |
| 8 | ENPP3 | 2.209 | 2.03E-03 | Up-regulated |
| 9 | DUOX1 | 2.192 | 2.35E-03 | Up-regulated |
| 10 | TRPM6 | 2.18 | 6.57E-03 | Up-regulated |
| 11 | FXYD4 | 2.168 | 2.41E-03 | Up-regulated |
| 12 | WNK4 | 2.152 | 5.29E-06 | Up-regulated |
| 13 | MARK2 | 2.096 | 1.24E-19 | Up-regulated |
| 14 | SLC39A14 | 2.046 | 2.75E-03 | Up-regulated |
| 15 | CDH16 | 2.037 | 2.52E-03 | Up-regulated |
| 16 | TFCP2L1 | 1.968 | 6.42E-06 | Up-regulated |
| 17 | MMP26 | 1.936 | 1.01E-02 | Up-regulated |
| 18 | CTSZ | 1.9 | 7.50E-13 | Up-regulated |
| 19 | MPZL2 | 1.891 | 2.59E-04 | Up-regulated |
| 20 | MEGF10 | 1.848 | 3.04E-03 | Up-regulated |
| 21 | SMIM24 | 1.845 | 1.82E-03 | Up-regulated |
| 22 | MAP2K6 | 1.843 | 1.02E-04 | Up-regulated |
| 23 | LCN12 | 1.832 | 1.06E-05 | Up-regulated |
| 24 | GP2 | 1.79 | 2.02E-02 | Up-regulated |
| 25 | PDE6A | 1.781 | 6.43E-04 | Up-regulated |
| 26 | EHF | 1.765 | 1.27E-10 | Up-regulated |
| 27 | RNF183 | 1.753 | 1.68E-05 | Up-regulated |
| 28 | CBLC | 1.742 | 3.57E-06 | Up-regulated |
| 29 | SULT2B1 | 1.736 | 1.16E-04 | Up-regulated |
| 30 | RAB27B | 1.731 | 2.57E-05 | Up-regulated |
| 31 | PLA2G4F | 1.724 | 3.45E-03 | Up-regulated |
| 32 | FAM107A | 1.698 | 2.05E-05 | Up-regulated |
| 33 | MUC1 | 1.686 | 4.02E-09 | Up-regulated |
| 34 | ATP12A | 1.673 | 1.87E-02 | Up-regulated |
| 35 | OPRK1 | 1.66 | 2.08E-02 | Up-regulated |
| 36 | SLC46A2 | 1.654 | 2.89E-03 | Up-regulated |
| 37 | FOLR1 | 1.651 | 5.66E-04 | Up-regulated |
| 38 | SCGB1D2 | 1.645 | 2.73E-04 | Up-regulated |
| 39 | SERPINA5 | 1.599 | 1.31E-03 | Up-regulated |
| 40 | RBM47 | 1.594 | 1.31E-18 | Up-regulated |
| 41 | ZC3H7B | 1.593 | 4.89E-10 | Up-regulated |
| 42 | TFPI2 | 1.586 | 5.65E-04 | Up-regulated |
| 43 | FETUB | 1.583 | 2.29E-02 | Up-regulated |
| 44 | CTNNA2 | 1.581 | 1.14E-02 | Up-regulated |
| 45 | BIRC3 | 1.578 | 1.04E-03 | Up-regulated |
| 46 | MCOLN2 | 1.545 | 7.53E-05 | Up-regulated |
| 47 | LATS1 | 1.521 | 8.95E-15 | Up-regulated |
| 48 | CAMP | 1.517 | 1.72E-02 | Up-regulated |
| 49 | SLC25A48 | 1.509 | 1.41E-02 | Up-regulated |
| 50 | HOMER2 | 1.503 | 4.11E-05 | Up-regulated |
| 51 | ALKAL2 | 1.502 | 3.45E-06 | Up-regulated |
| 52 | ATP13A4 | 1.488 | 3.45E-06 | Up-regulated |
| 53 | FAM155B | 1.474 | 8.54E-03 | Up-regulated |
| 54 | TTC21A | 1.474 | 7.56E-04 | Up-regulated |
| 55 | CES4A | 1.472 | 4.59E-04 | Up-regulated |
| 56 | PLCB1 | 1.472 | 1.88E-06 | Up-regulated |
| 57 | ALDH3B2 | 1.467 | 1.96E-03 | Up-regulated |
| 58 | VTCN1 | 1.46 | 7.23E-05 | Up-regulated |
| 59 | SIDT1 | 1.459 | 6.43E-04 | Up-regulated |
| 60 | NPR3 | 1.437 | 3.34E-03 | Up-regulated |
| 61 | PLA2G10 | 1.433 | 6.15E-03 | Up-regulated |
| 62 | VEPH1 | 1.433 | 4.97E-05 | Up-regulated |
| 63 | SULT1E1 | 1.421 | 2.09E-03 | Up-regulated |
| 64 | ADARB2 | 1.419 | 8.33E-03 | Up-regulated |
| 65 | FOLR3 | 1.412 | 1.10E-04 | Up-regulated |
| 66 | ERBB4 | 1.411 | 3.56E-05 | Up-regulated |
| 67 | KCNG1 | 1.411 | 2.02E-02 | Up-regulated |
| 68 | SLC25A29 | 1.409 | 1.61E-06 | Up-regulated |
| 69 | CHODL | 1.407 | 1.11E-02 | Up-regulated |
| 70 | LRRC1 | 1.406 | 1.72E-03 | Up-regulated |
| 71 | HSD11B2 | 1.402 | 2.82E-04 | Up-regulated |
| 72 | PTGS2 | 1.401 | 3.39E-04 | Up-regulated |
| 73 | SCGB1D1 | 1.392 | 2.32E-04 | Up-regulated |
| 74 | PTGS1 | 1.39 | 9.56E-04 | Up-regulated |
| 75 | SERPINA4 | 1.39 | 3.26E-03 | Up-regulated |
| 76 | CPT1A | 1.385 | 3.31E-06 | Up-regulated |
| 77 | CDC14A | 1.381 | 2.86E-06 | Up-regulated |
| 78 | PRKAG2 | 1.379 | 2.59E-06 | Up-regulated |
| 79 | UPK1B | 1.370 | 4.89E-02 | Up-regulated |
| 80 | NFIB | 1.364 | 3.90E-06 | Up-regulated |
| 81 | SOX7 | 1.358 | 3.65E-03 | Up-regulated |
| 82 | C20ORF96 | 1.352 | 3.84E-07 | Up-regulated |
| 83 | HOXB8 | 1.334 | 1.97E-08 | Up-regulated |
| 84 | FAM110C | 1.321 | 4.25E-03 | Up-regulated |
| 85 | SLAIN1 | 1.311 | 4.16E-03 | Up-regulated |
| 86 | ANK3 | 1.306 | 2.36E-03 | Up-regulated |
| 87 | OCA2 | 1.306 | 7.12E-04 | Up-regulated |
| 88 | TMEM61 | 1.306 | 1.48E-02 | Up-regulated |
| 89 | PLD5 | 1.303 | 2.07E-03 | Up-regulated |
| 90 | GMPR | 1.302 | 1.40E-03 | Up-regulated |
| 91 | PAPOLA | 1.302 | 1.05E-14 | Up-regulated |
| 92 | PPP1R1A | 1.302 | 5.71E-04 | Up-regulated |
| 93 | SLC39A2 | 1.302 | 7.56E-04 | Up-regulated |
| 94 | TMED6 | 1.301 | 3.83E-02 | Up-regulated |
| 95 | MOGAT1 | 1.3 | 1.27E-04 | Up-regulated |
| 96 | WWC1 | 1.297 | 3.57E-08 | Up-regulated |
| 97 | PHF24 | 1.296 | 8.04E-03 | Up-regulated |
| 98 | RALBP1 | 1.294 | 4.22E-14 | Up-regulated |
| 99 | CWH43 | 1.29 | 1.52E-03 | Up-regulated |
| 100 | HOOK3 | 1.289 | 4.99E-15 | Up-regulated |
| 101 | NLRP2 | 1.285 | 1.59E-03 | Up-regulated |
| 102 | ZDHHC11 | 1.285 | 3.49E-05 | Up-regulated |
| 103 | NFIA | 1.279 | 1.53E-05 | Up-regulated |
| 104 | PLCXD3 | 1.279 | 6.55E-03 | Up-regulated |
| 105 | KCNC3 | 1.277 | 1.83E-08 | Up-regulated |
| 106 | HECTD4 | 1.276 | 2.75E-10 | Up-regulated |
| 107 | PTK6 | 1.263 | 3.37E-04 | Up-regulated |
| 108 | MED18 | 1.259 | 1.08E-08 | Up-regulated |
| 109 | KRT31 | 1.253 | 4.27E-03 | Up-regulated |
| 110 | SLC24A4 | 1.248 | 3.28E-02 | Up-regulated |
| 111 | ASCL5 | 1.234 | 5.50E-10 | Up-regulated |
| 112 | GAL | 1.233 | 2.93E-02 | Up-regulated |
| 113 | EDA | 1.231 | 1.43E-05 | Up-regulated |
| 114 | ZBTB16 | 1.231 | 1.99E-02 | Up-regulated |
| 115 | SUSD4 | 1.223 | 1.38E-03 | Up-regulated |
| 116 | ZBED2 | 1.22 | 1.87E-02 | Up-regulated |
| 117 | CCDC71 | 1.217 | 2.51E-10 | Up-regulated |
| 118 | HGD | 1.211 | 8.64E-03 | Up-regulated |
| 119 | CABYR | 1.21 | 2.14E-05 | Up-regulated |
| 120 | PCSK6 | 1.207 | 1.89E-03 | Up-regulated |
| 121 | ARL17A | 1.206 | 3.84E-07 | Up-regulated |
| 122 | CKMT2 | 1.206 | 2.07E-04 | Up-regulated |
| 123 | NBEAL2 | 1.206 | 1.09E-07 | Up-regulated |
| 124 | PDZD8 | 1.203 | 2.77E-11 | Up-regulated |
| 125 | FKBP5 | 1.201 | 3.18E-02 | Up-regulated |
| 126 | MT1F | 1.199 | 1.58E-02 | Up-regulated |
| 127 | SLC43A1 | 1.18 | 1.71E-03 | Up-regulated |
| 128 | NTRK3 | 1.176 | 2.41E-04 | Up-regulated |
| 129 | SORD | 1.172 | 5.35E-03 | Up-regulated |
| 130 | WHRN | 1.171 | 1.28E-03 | Up-regulated |
| 131 | HES5 | 1.166 | 6.40E-05 | Up-regulated |
| 132 | EVX1 | 1.163 | 2.04E-12 | Up-regulated |
| 133 | PIWIL2 | 1.155 | 7.26E-03 | Up-regulated |
| 134 | ACTB | 1.15 | 1.59E-17 | Up-regulated |
| 135 | MYH14 | 1.148 | 6.35E-12 | Up-regulated |
| 136 | ST3GAL6 | 1.148 | 7.74E-03 | Up-regulated |
| 137 | MSLN | 1.147 | 2.81E-04 | Up-regulated |
| 138 | OVOL1 | 1.147 | 1.83E-03 | Up-regulated |
| 139 | PRR15L | 1.147 | 2.20E-02 | Up-regulated |
| 140 | ENTPD3 | 1.146 | 1.13E-02 | Up-regulated |
| 141 | RAB11FIP4 | 1.146 | 1.34E-06 | Up-regulated |
| 142 | SLC26A7 | 1.141 | 4.37E-04 | Up-regulated |
| 143 | SLC4A7 | 1.14 | 3.23E-03 | Up-regulated |
| 144 | LGR5 | 1.138 | 3.33E-05 | Up-regulated |
| 145 | SLC38A2 | 1.138 | 1.59E-09 | Up-regulated |
| 146 | MICALCL | 1.134 | 5.17E-04 | Up-regulated |
| 147 | TCF7L1 | 1.13 | 7.37E-07 | Up-regulated |
| 148 | ABCG1 | 1.128 | 7.47E-03 | Up-regulated |
| 149 | LONRF3 | 1.128 | 1.66E-03 | Up-regulated |
| 150 | SLC34A2 | 1.121 | 4.05E-04 | Up-regulated |
| 151 | SPATA13 | 1.119 | 8.89E-11 | Up-regulated |
| 152 | MOB1A | 1.117 | 9.77E-17 | Up-regulated |
| 153 | SERHL2 | 1.117 | 5.15E-03 | Up-regulated |
| 154 | FAM83B | 1.116 | 4.82E-09 | Up-regulated |
| 155 | HOXB3 | 1.104 | 2.86E-09 | Up-regulated |
| 156 | USP33 | 1.104 | 2.71E-14 | Up-regulated |
| 157 | SH3RF2 | 1.103 | 1.88E-04 | Up-regulated |
| 158 | RANBP17 | 1.091 | 1.32E-03 | Up-regulated |
| 159 | AXIN2 | 1.089 | 4.78E-07 | Up-regulated |
| 160 | TNFRSF13C | 1.086 | 2.30E-06 | Up-regulated |
| 161 | LRRTM1 | 1.085 | 6.90E-03 | Up-regulated |
| 162 | DUSP2 | 1.083 | 5.02E-03 | Up-regulated |
| 163 | TESMIN | 1.081 | 2.41E-03 | Up-regulated |
| 164 | RXFP1 | 1.078 | 1.83E-02 | Up-regulated |
| 165 | TXNDC16 | 1.078 | 4.88E-04 | Up-regulated |
| 166 | PDK4 | 1.077 | 1.07E-02 | Up-regulated |
| 167 | LPAR3 | 1.075 | 1.22E-04 | Up-regulated |
| 168 | SLC23A1 | 1.075 | 1.20E-04 | Up-regulated |
| 169 | CAPS | 1.069 | 3.70E-07 | Up-regulated |
| 170 | ABCA2 | 1.067 | 3.13E-07 | Up-regulated |
| 171 | MAB21L3 | 1.067 | 4.96E-04 | Up-regulated |
| 172 | WFDC2 | 1.067 | 2.13E-04 | Up-regulated |
| 173 | UNC5B | 1.066 | 3.15E-04 | Up-regulated |
| 174 | NEK10 | 1.065 | 2.41E-03 | Up-regulated |
| 175 | FBXL16 | 1.06 | 4.95E-02 | Up-regulated |
| 176 | JUP | 1.059 | 1.74E-05 | Up-regulated |
| 177 | CA4 | 1.058 | 1.88E-03 | Up-regulated |
| 178 | COL23A1 | 1.053 | 6.61E-05 | Up-regulated |
| 179 | DRC1 | 1.052 | 1.24E-03 | Up-regulated |
| 180 | ARHGAP32 | 1.051 | 6.30E-12 | Up-regulated |
| 181 | BAIAP2 | 1.051 | 8.12E-07 | Up-regulated |
| 182 | TM7SF2 | 1.051 | 1.24E-03 | Up-regulated |
| 183 | KCNMA1 | 1.047 | 4.45E-06 | Up-regulated |
| 184 | PBX2 | 1.047 | 5.42E-11 | Up-regulated |
| 185 | RGS9BP | 1.047 | 6.14E-03 | Up-regulated |
| 186 | ADD1 | 1.045 | 2.20E-11 | Up-regulated |
| 187 | TOMM20L | 1.044 | 5.00E-07 | Up-regulated |
| 188 | PIP5K1B | 1.036 | 1.51E-03 | Up-regulated |
| 189 | R3HDM4 | 1.036 | 4.11E-12 | Up-regulated |
| 190 | IGSF9 | 1.03 | 8.54E-04 | Up-regulated |
| 191 | TLR5 | 1.028 | 2.81E-03 | Up-regulated |
| 192 | FGD4 | 1.026 | 9.70E-05 | Up-regulated |
| 193 | MROH1 | 1.026 | 2.19E-07 | Up-regulated |
| 194 | NEBL | 1.025 | 5.66E-03 | Up-regulated |
| 195 | PCDH1 | 1.025 | 3.22E-04 | Up-regulated |
| 196 | ALPL | 1.024 | 2.87E-02 | Up-regulated |
| 197 | AVPR1A | 1.024 | 5.51E-05 | Up-regulated |
| 198 | TDRD9 | 1.023 | 4.85E-03 | Up-regulated |
| 199 | SMAD9 | 1.02 | 3.23E-04 | Up-regulated |
| 200 | CTAGE4 | 1.019 | 9.55E-07 | Up-regulated |
| 201 | DNAH9 | 1.019 | 2.31E-03 | Up-regulated |
| 202 | OR7A5 | 1.018 | 1.89E-02 | Up-regulated |
| 203 | USP53 | 1.011 | 7.52E-04 | Up-regulated |
| 204 | SGCD | 1.009 | 1.32E-02 | Up-regulated |
| 205 | SLITRK5 | 1.009 | 7.35E-03 | Up-regulated |
| 206 | SQLE | 1.007 | 4.69E-03 | Up-regulated |
| 207 | IRF6 | 1.006 | 5.35E-07 | Up-regulated |
| 208 | STOML3 | 1.005 | 4.75E-05 | Up-regulated |
| 209 | ZMYND11 | 1.003 | 2.15E-03 | Up-regulated |
| 210 | DAPK2 | 1.002 | 2.69E-04 | Up-regulated |
| 211 | VIRMA | 1.002 | 2.97E-16 | Up-regulated |
| 212 | CHRNA6 | 1.001 | 6.75E-03 | Up-regulated |
| 213 | FBRSL1 | 1.001 | 8.95E-10 | Up-regulated |
| 214 | FHAD1 | 1.001 | 1.60E-04 | Up-regulated |
| 215 | RNASE6 | -1 | 4.68E-08 | Down-regulated |
| 216 | PDPN | -1.002 | 3.77E-04 | Down-regulated |
| 217 | CENPV | -1.003 | 1.79E-07 | Down-regulated |
| 218 | QTRT1 | -1.003 | 1.50E-07 | Down-regulated |
| 219 | ADORA3 | -1.004 | 6.85E-05 | Down-regulated |
| 220 | PEX5 | -1.005 | 6.40E-08 | Down-regulated |
| 221 | FOXM1 | -1.006 | 1.61E-02 | Down-regulated |
| 222 | CENPU | -1.008 | 4.46E-03 | Down-regulated |
| 223 | JAKMIP2 | -1.013 | 2.79E-05 | Down-regulated |
| 224 | TGM2 | -1.013 | 2.67E-04 | Down-regulated |
| 225 | POLE2 | -1.015 | 1.30E-04 | Down-regulated |
| 226 | RASGRF1 | -1.015 | 4.94E-03 | Down-regulated |
| 227 | PSMD10 | -1.016 | 9.72E-12 | Down-regulated |
| 228 | ANXA2 | -1.018 | 5.20E-05 | Down-regulated |
| 229 | CPVL | -1.019 | 5.18E-06 | Down-regulated |
| 230 | PARG | -1.02 | 5.65E-07 | Down-regulated |
| 231 | MORF4L1 | -1.026 | 2.93E-07 | Down-regulated |
| 232 | CXCL10 | -1.027 | 1.69E-03 | Down-regulated |
| 233 | HNRNPAB | -1.029 | 3.20E-07 | Down-regulated |
| 234 | KDR | -1.029 | 1.21E-05 | Down-regulated |
| 235 | SYT11 | -1.029 | 6.97E-06 | Down-regulated |
| 236 | PARP8 | -1.03 | 2.36E-08 | Down-regulated |
| 237 | PSG8 | -1.03 | 1.57E-02 | Down-regulated |
| 238 | COQ3 | -1.032 | 9.64E-13 | Down-regulated |
| 239 | CDH3 | -1.033 | 3.62E-02 | Down-regulated |
| 240 | SLC7A7 | -1.035 | 1.68E-04 | Down-regulated |
| 241 | AIF1L | -1.036 | 2.70E-05 | Down-regulated |
| 242 | MARCH5 | -1.037 | 1.03E-08 | Down-regulated |
| 243 | BNC2 | -1.038 | 3.32E-04 | Down-regulated |
| 244 | CHI3L2 | -1.038 | 1.15E-02 | Down-regulated |
| 245 | KLHL13 | -1.04 | 1.61E-06 | Down-regulated |
| 246 | LEFTY2 | -1.041 | 8.16E-03 | Down-regulated |
| 247 | NKX2-1 | -1.043 | 1.29E-03 | Down-regulated |
| 248 | TNFRSF1B | -1.044 | 2.24E-05 | Down-regulated |
| 249 | ADAM19 | -1.045 | 1.62E-06 | Down-regulated |
| 250 | EXO1 | -1.046 | 4.56E-03 | Down-regulated |
| 251 | LRRC2 | -1.046 | 2.57E-03 | Down-regulated |
| 252 | GTF2A1L | -1.048 | 7.17E-04 | Down-regulated |
| 253 | RAB32 | -1.048 | 1.72E-09 | Down-regulated |
| 254 | APMAP | -1.051 | 3.38E-09 | Down-regulated |
| 255 | KLRC1 | -1.051 | 3.81E-03 | Down-regulated |
| 256 | SUPT16H | -1.051 | 6.41E-06 | Down-regulated |
| 257 | TNC | -1.051 | 4.97E-03 | Down-regulated |
| 258 | HNRNPA1L2 | -1.052 | 6.90E-08 | Down-regulated |
| 259 | KIFC1 | -1.053 | 1.00E-02 | Down-regulated |
| 260 | GPR68 | -1.054 | 5.35E-06 | Down-regulated |
| 261 | MUC13 | -1.054 | 6.20E-04 | Down-regulated |
| 262 | LDHA | -1.057 | 8.35E-05 | Down-regulated |
| 263 | SPC25 | -1.057 | 1.19E-02 | Down-regulated |
| 264 | ADAMTS4 | -1.058 | 1.00E-05 | Down-regulated |
| 265 | KIR2DS2 | -1.06 | 9.20E-03 | Down-regulated |
| 266 | MYADM | -1.06 | 5.68E-07 | Down-regulated |
| 267 | BIN2 | -1.061 | 1.88E-07 | Down-regulated |
| 268 | BCL11B | -1.063 | 3.24E-07 | Down-regulated |
| 269 | SFRP1 | -1.064 | 2.30E-02 | Down-regulated |
| 270 | SLC13A1 | -1.064 | 2.09E-04 | Down-regulated |
| 271 | SPC24 | -1.066 | 1.20E-03 | Down-regulated |
| 272 | CHRDL1 | -1.068 | 5.26E-03 | Down-regulated |
| 273 | SMTNL2 | -1.069 | 1.61E-03 | Down-regulated |
| 274 | CDC25B | -1.072 | 1.55E-07 | Down-regulated |
| 275 | VDAC3 | -1.075 | 4.41E-12 | Down-regulated |
| 276 | BDKRB1 | -1.076 | 1.25E-04 | Down-regulated |
| 277 | EOMES | -1.078 | 7.18E-04 | Down-regulated |
| 278 | CCNYL1 | -1.079 | 7.39E-08 | Down-regulated |
| 279 | VIPR2 | -1.08 | 1.62E-04 | Down-regulated |
| 280 | CENPH | -1.081 | 3.02E-04 | Down-regulated |
| 281 | MINPP1 | -1.083 | 3.46E-10 | Down-regulated |
| 282 | OLFM1 | -1.083 | 1.41E-02 | Down-regulated |
| 283 | ARHGEF26 | -1.088 | 2.51E-03 | Down-regulated |
| 284 | KLRC3 | -1.093 | 5.79E-04 | Down-regulated |
| 285 | SCARF2 | -1.093 | 3.27E-14 | Down-regulated |
| 286 | EPHA3 | -1.094 | 5.14E-05 | Down-regulated |
| 287 | ALG1 | -1.097 | 9.22E-06 | Down-regulated |
| 288 | HNRNPH3 | -1.099 | 1.30E-08 | Down-regulated |
| 289 | S100A9 | -1.1 | 1.12E-02 | Down-regulated |
| 290 | F13A1 | -1.104 | 5.75E-03 | Down-regulated |
| 291 | CA12 | -1.105 | 4.79E-03 | Down-regulated |
| 292 | MGP | -1.105 | 1.21E-03 | Down-regulated |
| 293 | MAMLD1 | -1.11 | 1.47E-06 | Down-regulated |
| 294 | PGBD5 | -1.11 | 1.03E-03 | Down-regulated |
| 295 | RGS20 | -1.11 | 1.58E-03 | Down-regulated |
| 296 | NKAIN1 | -1.112 | 8.96E-03 | Down-regulated |
| 297 | SGK223 | -1.113 | 3.75E-05 | Down-regulated |
| 298 | KCNAB3 | -1.114 | 8.89E-05 | Down-regulated |
| 299 | EFEMP1 | -1.116 | 5.23E-03 | Down-regulated |
| 300 | RABGAP1L | -1.116 | 1.18E-11 | Down-regulated |
| 301 | NAGA | -1.121 | 1.31E-09 | Down-regulated |
| 302 | GIMAP2 | -1.129 | 2.29E-11 | Down-regulated |
| 303 | CDH13 | -1.135 | 5.32E-04 | Down-regulated |
| 304 | TRIM46 | -1.137 | 1.02E-13 | Down-regulated |
| 305 | SORBS1 | -1.139 | 4.09E-04 | Down-regulated |
| 306 | CKAP2L | -1.14 | 1.98E-03 | Down-regulated |
| 307 | RRM2 | -1.141 | 7.79E-03 | Down-regulated |
| 308 | RAPGEF6 | -1.142 | 2.06E-13 | Down-regulated |
| 309 | CASKIN1 | -1.143 | 1.89E-11 | Down-regulated |
| 310 | CXCL11 | -1.143 | 5.40E-03 | Down-regulated |
| 311 | RAB38 | -1.145 | 6.14E-04 | Down-regulated |
| 312 | ADAMTS16 | -1.153 | 1.36E-02 | Down-regulated |
| 313 | CD109 | -1.158 | 2.31E-05 | Down-regulated |
| 314 | IL2RA | -1.158 | 1.77E-10 | Down-regulated |
| 315 | PCSK1N | -1.159 | 1.90E-13 | Down-regulated |
| 316 | CDC5L | -1.16 | 4.14E-05 | Down-regulated |
| 317 | ALOX5AP | -1.163 | 1.29E-05 | Down-regulated |
| 318 | BRK1 | -1.169 | 1.07E-12 | Down-regulated |
| 319 | EDNRB | -1.173 | 4.31E-03 | Down-regulated |
| 320 | CCL23 | -1.176 | 1.78E-04 | Down-regulated |
| 321 | MELTF | -1.184 | 2.86E-05 | Down-regulated |
| 322 | TPM1 | -1.186 | 3.35E-07 | Down-regulated |
| 323 | SNCA | -1.189 | 4.13E-05 | Down-regulated |
| 324 | PPARGC1A | -1.19 | 4.88E-03 | Down-regulated |
| 325 | ASS1 | -1.192 | 1.54E-04 | Down-regulated |
| 326 | GBP1 | -1.196 | 1.26E-06 | Down-regulated |
| 327 | PITHD1 | -1.196 | 4.87E-13 | Down-regulated |
| 328 | PRL | -1.197 | 3.52E-04 | Down-regulated |
| 329 | CAV2 | -1.203 | 3.93E-08 | Down-regulated |
| 330 | ACKR1 | -1.205 | 7.05E-03 | Down-regulated |
| 331 | SLC43A3 | -1.209 | 6.71E-06 | Down-regulated |
| 332 | SYT13 | -1.211 | 1.50E-02 | Down-regulated |
| 333 | ASPN | -1.215 | 1.15E-05 | Down-regulated |
| 334 | GNG4 | -1.215 | 1.28E-05 | Down-regulated |
| 335 | CPXM2 | -1.217 | 3.36E-03 | Down-regulated |
| 336 | INHBA | -1.219 | 7.19E-04 | Down-regulated |
| 337 | ASTN1 | -1.22 | 2.06E-03 | Down-regulated |
| 338 | MLPH | -1.221 | 2.61E-03 | Down-regulated |
| 339 | CTHRC1 | -1.226 | 4.42E-08 | Down-regulated |
| 340 | OAS2 | -1.228 | 8.38E-06 | Down-regulated |
| 341 | CHST11 | -1.236 | 4.13E-06 | Down-regulated |
| 342 | SST | -1.249 | 1.43E-02 | Down-regulated |
| 343 | UCHL1 | -1.25 | 1.13E-04 | Down-regulated |
| 344 | AKR1B15 | -1.252 | 3.09E-02 | Down-regulated |
| 345 | LPCAT1 | -1.252 | 9.99E-11 | Down-regulated |
| 346 | STK19 | -1.263 | 2.50E-07 | Down-regulated |
| 347 | AVP | -1.268 | 3.34E-11 | Down-regulated |
| 348 | CXCL12 | -1.27 | 3.35E-04 | Down-regulated |
| 349 | CALHM6 | -1.276 | 7.78E-08 | Down-regulated |
| 350 | BST2 | -1.289 | 6.45E-06 | Down-regulated |
| 351 | MFAP4 | -1.292 | 6.42E-05 | Down-regulated |
| 352 | MS4A6A | -1.292 | 7.11E-09 | Down-regulated |
| 353 | TMEM35A | -1.301 | 1.93E-05 | Down-regulated |
| 354 | FANCD2 | -1.309 | 8.90E-06 | Down-regulated |
| 355 | WLS | -1.323 | 3.64E-05 | Down-regulated |
| 356 | AGT | -1.326 | 1.05E-03 | Down-regulated |
| 357 | UGT1A10 | -1.337 | 2.38E-02 | Down-regulated |
| 358 | SLC1A1 | -1.338 | 4.03E-02 | Down-regulated |
| 359 | TNFAIP6 | -1.339 | 2.49E-03 | Down-regulated |
| 360 | ACTA2 | -1.346 | 2.79E-05 | Down-regulated |
| 361 | LIF | -1.353 | 1.77E-02 | Down-regulated |
| 362 | SDHC | -1.357 | 9.00E-09 | Down-regulated |
| 363 | PRKAG1 | -1.36 | 2.76E-10 | Down-regulated |
| 364 | VCAM1 | -1.37 | 1.96E-06 | Down-regulated |
| 365 | FUS | -1.379 | 2.46E-10 | Down-regulated |
| 366 | CYP3A5 | -1.382 | 4.82E-02 | Down-regulated |
| 367 | PCDH17 | -1.422 | 1.64E-05 | Down-regulated |
| 368 | TOP2A | -1.442 | 7.57E-04 | Down-regulated |
| 369 | GAS1 | -1.459 | 3.63E-05 | Down-regulated |
| 370 | BRINP2 | -1.495 | 6.18E-04 | Down-regulated |
| 371 | DOK5 | -1.497 | 6.90E-04 | Down-regulated |
| 372 | C1ORF229 | -1.5 | 4.97E-16 | Down-regulated |
| 373 | FOXB1 | -1.502 | 5.46E-11 | Down-regulated |
| 374 | RSPO3 | -1.509 | 1.39E-06 | Down-regulated |
| 375 | PLPP4 | -1.539 | 2.69E-04 | Down-regulated |
| 376 | AOX1 | -1.54 | 4.63E-02 | Down-regulated |
| 377 | PAPSS2 | -1.623 | 5.20E-06 | Down-regulated |
| 378 | CDKN3 | -1.633 | 3.96E-04 | Down-regulated |
| 379 | RAD51AP1 | -1.686 | 4.43E-06 | Down-regulated |
| 380 | GJA1 | -1.705 | 1.79E-07 | Down-regulated |
| 381 | HAPLN1 | -1.727 | 2.58E-05 | Down-regulated |
| 382 | GLT1D1 | -1.766 | 9.64E-05 | Down-regulated |
| 383 | PENK | -1.842 | 7.25E-03 | Down-regulated |
| 384 | PDZK1 | -1.904 | 1.54E-03 | Down-regulated |
| 385 | CNN1 | -1.917 | 1.65E-05 | Down-regulated |
| 386 | ENC1 | -1.93 | 6.84E-07 | Down-regulated |

**Supplementary table 2. significantly upregulated and downregulated microRNAs in the GSE71332 dataset.**

|  | miRNA_ID | logFC | P-value | Expression |
| --- | --- | --- | --- | --- |
| 1 | hsa-miR-186-5p | 6.799 | 2.61E-10 | Up-regulated |
| 2 | hsa-miR-135b-5p | 6.445 | 1.68E-09 | Up-regulated |
| 3 | hsa-miR-3125 | 6.259 | 9.44E-11 | Up-regulated |
| 4 | hsa-miR-136-5p | 6.198 | 1.03E-05 | Up-regulated |
| 5 | hsa-miR-204-5p | 6.179 | 2.50E-05 | Up-regulated |
| 6 | hsa-miR-3907 | 6.176 | 9.85E-10 | Up-regulated |
| 7 | hsa-miR-30d-3p | 6.085 | 8.86E-09 | Up-regulated |
| 8 | hsa-miR-1288-3p | 6.053 | 1.18E-11 | Up-regulated |
| 9 | hsa-miR-371b-5p | 5.906 | 2.39E-04 | Up-regulated |
| 10 | hsa-miR-374c-5p | 5.737 | 2.93E-11 | Up-regulated |
| 11 | hsa-miR-32-5p | 5.423 | 1.57E-09 | Up-regulated |
| 12 | hsa-miR-6512-5p | 5.338 | 2.72E-04 | Up-regulated |
| 13 | hsa-miR-1914-3p | 5.162 | 1.99E-05 | Up-regulated |
| 14 | hsa-miR-205-5p | 5.005 | 8.25E-04 | Up-regulated |
| 15 | hsa-miR-505-3p | 4.881 | 5.92E-05 | Up-regulated |
| 16 | hsa-miR-7-1-3p | 4.875 | 9.21E-05 | Up-regulated |
| 17 | hsa-miR-449b-5p | 4.859 | 5.81E-05 | Up-regulated |
| 18 | hsa-miR-145-3p | 4.818 | 1.27E-04 | Up-regulated |
| 19 | hsa-miR-4734 | 4.743 | 1.34E-04 | Up-regulated |
| 20 | hsa-miR-144-5p | 4.695 | 1.11E-04 | Up-regulated |
| 21 | hsa-miR-9-5p | 4.640 | 4.32E-04 | Up-regulated |
| 22 | hsa-miR-4690-5p | 4.602 | 1.12E-04 | Up-regulated |
| 23 | hsa-miR-744-5p | 4.299 | 5.58E-04 | Up-regulated |
| 24 | hsa-miR-4486 | 4.244 | 5.92E-03 | Up-regulated |
| 25 | hsa-miR-6132 | 4.193 | 4.70E-03 | Up-regulated |
| 26 | hsa-miR-149-5p | 4.117 | 1.75E-03 | Up-regulated |
| 27 | hsa-miR-1307-5p | 4.057 | 3.17E-04 | Up-regulated |
| 28 | hsa-miR-424-3p | 4.002 | 1.90E-03 | Up-regulated |
| 29 | hsa-miR-4324 | 3.949 | 9.42E-03 | Up-regulated |
| 30 | hsa-miR-154-5p | 3.898 | 2.96E-03 | Up-regulated |
| 31 | hsa-miR-10a-3p | 3.897 | 6.74E-03 | Up-regulated |
| 32 | hsa-miR-141-5p | 3.872 | 1.18E-03 | Up-regulated |
| 33 | hsa-miR-501-3p | 3.859 | 1.57E-03 | Up-regulated |
| 34 | hsa-miR-1290 | 3.824 | 5.12E-03 | Up-regulated |
| 35 | hsa-miR-206 | 3.820 | 1.87E-02 | Up-regulated |
| 36 | hsa-miR-4257 | 3.773 | 7.21E-03 | Up-regulated |
| 37 | hsa-miR-1234-3p | 3.772 | 2.80E-02 | Up-regulated |
| 38 | hsa-miR-3127-5p | 3.756 | 2.17E-02 | Up-regulated |
| 39 | hsa-miR-375-3p | 3.744 | 3.69E-03 | Up-regulated |
| 40 | hsa-miR-3156-5p | 3.701 | 5.56E-03 | Up-regulated |
| 41 | hsa-miR-598-3p | 3.548 | 2.57E-03 | Up-regulated |
| 42 | hsa-miR-5088-5p | 3.518 | 8.62E-03 | Up-regulated |
| 43 | hsa-miR-4746-3p | 3.479 | 7.47E-03 | Up-regulated |
| 44 | hsa-miR-4726-5p | 3.466 | 1.87E-02 | Up-regulated |
| 45 | hsa-miR-4656 | 3.451 | 5.32E-03 | Up-regulated |
| 46 | hsa-miR-33a-5p | 3.403 | 8.67E-03 | Up-regulated |
| 47 | hsa-let-7i-3p | 3.309 | 6.27E-03 | Up-regulated |
| 48 | hsa-miR-34c-3p | 3.294 | 6.45E-03 | Up-regulated |
| 49 | hsa-miR-1285-3p | 3.291 | 5.51E-03 | Up-regulated |
| 50 | hsa-miR-29c-5p | 3.287 | 6.62E-03 | Up-regulated |
| 51 | hsa-miR-215-5p | 3.226 | 3.33E-02 | Up-regulated |
| 52 | hsa-miR-16-2-3p | 3.222 | 1.13E-03 | Up-regulated |
| 53 | hsa-miR-142-3p | 3.134 | 3.17E-05 | Up-regulated |
| 54 | hsa-miR-34a-3p | 3.129 | 4.94E-03 | Up-regulated |
| 55 | hsa-miR-4695-5p | 3.077 | 9.93E-03 | Up-regulated |
| 56 | hsa-miR-205-3p | 3.027 | 3.09E-02 | Up-regulated |
| 57 | hsa-miR-193a-5p | 2.982 | 1.07E-02 | Up-regulated |
| 58 | hsa-miR-374a-5p | 2.953 | 7.77E-06 | Up-regulated |
| 59 | hsa-miR-181a-3p | 2.909 | 4.85E-02 | Up-regulated |
| 60 | hsa-miR-182-5p | 2.831 | 7.60E-03 | Up-regulated |
| 61 | hsa-miR-3188 | 2.807 | 3.88E-02 | Up-regulated |
| 62 | hsa-miR-203a-3p | 2.790 | 6.93E-03 | Up-regulated |
| 63 | hsa-miR-301b-3p | 2.787 | 8.23E-03 | Up-regulated |
| 64 | hsa-miR-3926 | 2.775 | 3.68E-02 | Up-regulated |
| 65 | hsa-miR-4745-5p | 2.751 | 2.79E-02 | Up-regulated |
| 66 | hsa-miR-450a-5p | 2.727 | 1.87E-02 | Up-regulated |
| 67 | hsa-miR-6131 | 2.690 | 2.11E-02 | Up-regulated |
| 68 | hsa-miR-200a-5p | 2.626 | 2.89E-02 | Up-regulated |
| 69 | hsa-miR-887-3p | 2.610 | 6.85E-03 | Up-regulated |
| 70 | hsa-miR-19b-1-5p | 2.599 | 1.10E-02 | Up-regulated |
| 71 | hsa-miR-590-5p | 2.574 | 6.45E-03 | Up-regulated |
| 72 | hsa-miR-125b-2-3p | 2.573 | 2.95E-02 | Up-regulated |
| 73 | hsa-miR-664b-5p | 2.542 | 3.84E-02 | Up-regulated |
| 74 | hsa-miR-139-3p | 2.499 | 3.85E-02 | Up-regulated |
| 75 | hsa-miR-200c-5p | 2.493 | 7.98E-03 | Up-regulated |
| 76 | hsa-miR-181a-2-3p | 2.485 | 3.63E-02 | Up-regulated |
| 77 | hsa-miR-664a-5p | 2.445 | 3.93E-02 | Up-regulated |
| 78 | hsa-miR-1471 | 2.437 | 4.05E-02 | Up-regulated |
| 79 | hsa-miR-214-5p | 2.432 | 1.13E-02 | Up-regulated |
| 80 | hsa-miR-30e-3p | 2.427 | 9.00E-03 | Up-regulated |
| 81 | hsa-miR-183-5p | 2.404 | 3.55E-02 | Up-regulated |
| 82 | hsa-miR-340-3p | 2.399 | 3.97E-02 | Up-regulated |
| 83 | hsa-miR-502-5p | 2.376 | 3.01E-02 | Up-regulated |
| 84 | hsa-miR-760 | 2.352 | 4.04E-02 | Up-regulated |
| 85 | hsa-miR-218-5p | 2.257 | 2.03E-02 | Up-regulated |
| 86 | hsa-miR-423-3p | 2.234 | 1.16E-02 | Up-regulated |
| 87 | hsa-miR-99a-3p | 2.209 | 4.46E-02 | Up-regulated |
| 88 | hsa-miR-339-3p | 2.153 | 4.29E-02 | Up-regulated |
| 89 | hsa-miR-455-5p | 2.013 | 3.56E-04 | Up-regulated |
| 90 | hsa-miR-136-3p | 1.838 | 3.21E-02 | Up-regulated |
| 91 | hsa-miR-30c-5p | 1.810 | 2.82E-05 | Up-regulated |
| 92 | hsa-miR-1260b | 1.753 | 1.69E-04 | Up-regulated |
| 93 | hsa-miR-145-5p | 1.679 | 3.84E-04 | Up-regulated |
| 94 | hsa-miR-362-3p | 1.609 | 1.87E-04 | Up-regulated |
| 95 | hsa-miR-374b-5p | 1.585 | 5.85E-05 | Up-regulated |
| 96 | hsa-miR-30b-5p | 1.553 | 8.72E-05 | Up-regulated |
| 97 | hsa-miR-429 | 1.438 | 3.78E-04 | Up-regulated |
| 98 | hsa-miR-4428 | 1.429 | 1.51E-04 | Up-regulated |
| 99 | hsa-miR-196b-5p | 1.345 | 2.58E-03 | Up-regulated |
| 100 | hsa-miR-199a-5p | 1.342 | 2.20E-04 | Up-regulated |
| 101 | hsa-miR-199b-5p | 1.315 | 1.74E-03 | Up-regulated |
| 102 | hsa-miR-34c-5p | 1.292 | 4.44E-02 | Up-regulated |
| 103 | hsa-miR-143-3p | 1.289 | 1.80E-03 | Up-regulated |
| 104 | hsa-miR-449a | 1.228 | 2.53E-02 | Up-regulated |
| 105 | hsa-miR-6717-5p | 1.206 | 3.49E-04 | Up-regulated |
| 106 | hsa-miR-301a-3p | 1.204 | 2.55E-03 | Up-regulated |
| 107 | hsa-miR-424-5p | 1.132 | 6.05E-03 | Up-regulated |
| 108 | hsa-miR-335-5p | 1.105 | 8.35E-04 | Up-regulated |
| 109 | hsa-miR-125b-5p | 1.094 | 2.79E-03 | Up-regulated |
| 110 | hsa-miR-1305 | 1.079 | 1.32E-04 | Up-regulated |
| 111 | hsa-miR-365a-3p | 1.068 | 4.99E-04 | Up-regulated |
| 112 | hsa-miR-146b-5p | 1.045 | 8.83E-03 | Up-regulated |
| 113 | hsa-miR-21-5p | 1.032 | 4.00E-03 | Up-regulated |
| 114 | hsa-miR-572 | -1.057 | 2.90E-02 | Down-regulated |
| 115 | hsa-miR-6165 | -1.094 | 5.30E-04 | Down-regulated |
| 116 | hsa-miR-5739 | -1.112 | 2.75E-03 | Down-regulated |
| 117 | hsa-miR-6088 | -1.118 | 6.50E-04 | Down-regulated |
| 118 | hsa-miR-1229-5p | -1.123 | 3.58E-02 | Down-regulated |
| 119 | hsa-miR-4788 | -1.184 | 8.59E-04 | Down-regulated |
| 120 | hsa-miR-3162-3p | -1.194 | 3.58E-03 | Down-regulated |
| 121 | hsa-miR-4306 | -1.194 | 6.85E-05 | Down-regulated |
| 122 | hsa-miR-1207-5p | -1.420 | 1.11E-03 | Down-regulated |
| 123 | hsa-miR-134-5p | -1.472 | 6.91E-04 | Down-regulated |
| 124 | hsa-miR-3622b-3p | -1.848 | 4.00E-02 | Down-regulated |
| 125 | hsa-miR-3617-3p | -1.850 | 4.01E-02 | Down-regulated |
| 126 | hsa-miR-4740-3p | -1.854 | 4.03E-02 | Down-regulated |
| 127 | hsa-miR-4297 | -1.862 | 4.07E-02 | Down-regulated |
| 128 | hsa-miR-2277-3p | -1.943 | 4.48E-02 | Down-regulated |
| 129 | hsa-miR-1910-5p | -2.039 | 4.97E-02 | Down-regulated |
| 130 | hsa-miR-4290 | -2.153 | 4.61E-02 | Down-regulated |
| 131 | hsa-miR-328-3p | -2.266 | 3.11E-02 | Down-regulated |
| 132 | hsa-miR-939-3p | -2.859 | 1.95E-02 | Down-regulated |
| 133 | hsa-miR-1224-3p | -2.885 | 4.15E-02 | Down-regulated |
| 134 | hsa-miR-631 | -2.896 | 1.96E-02 | Down-regulated |
| 135 | hsa-miR-1296-5p | -2.943 | 1.99E-02 | Down-regulated |
| 136 | hsa-miR-122b-3p | -2.970 | 2.03E-02 | Down-regulated |
| 137 | hsa-miR-4763-5p | -3.076 | 2.06E-02 | Down-regulated |
| 138 | hsa-miR-449b-3p | -3.089 | 2.08E-02 | Down-regulated |
| 139 | hsa-miR-449c-3p | -3.101 | 2.57E-02 | Down-regulated |
| 140 | hsa-miR-4695-3p | -3.176 | 2.13E-02 | Down-regulated |
| 141 | hsa-miR-4689 | -3.229 | 1.19E-02 | Down-regulated |
| 142 | hsa-miR-4701-5p | -3.732 | 1.29E-02 | Down-regulated |
| 143 | hsa-miR-4254 | -3.867 | 2.29E-03 | Down-regulated |
| 144 | hsa-miR-4668-5p | -6.694 | 7.57E-05 | Down-regulated |

**Supplementary table 3. The significantly upregulated and downregulated circular RNAs in the GSE147442 dataset.**

|  | circRNA_ID | logFC | P-Value | Expression |
| --- | --- | --- | --- | --- |
| 1 | hsa_circ_0001568 | 4.088 | 8.00E-03 | Up-regulated |
| 2 | hsa_circ_0056386 | 3.066 | 0.00E+00 | Up-regulated |
| 3 | hsa_circ_0092337 | 2.956 | 1.00E-03 | Up-regulated |
| 4 | hsa_circ_0034762 | 2.887 | 1.00E-02 | Up-regulated |
| 5 | hsa_circ_0004121 | 2.846 | 7.00E-03 | Up-regulated |
| 6 | hsa_circ_0071915 | 2.765 | 6.00E-03 | Up-regulated |
| 7 | hsa_circ_0002739 | 2.748 | 1.00E-03 | Up-regulated |
| 8 | hsa_circ_0034642 | 2.686 | 4.00E-03 | Up-regulated |
| 9 | hsa_circ_0089762 | 2.685 | 1.00E-03 | Up-regulated |
| 10 | hsa_circ_0041821 | 2.654 | 3.00E-03 | Up-regulated |
| 11 | hsa_circ_0072665 | 2.615 | 5.00E-03 | Up-regulated |
| 12 | hsa_circ_0040994 | 2.611 | 9.00E-03 | Up-regulated |
| 13 | hsa_circ_0006853 | 2.586 | 1.00E-03 | Up-regulated |
| 14 | hsa_circ_0054912 | 2.576 | 4.00E-03 | Up-regulated |
| 15 | hsa_circ_0041100 | 2.562 | 3.00E-03 | Up-regulated |
| 16 | hsa_circ_0001005 | 2.553 | 0.00E+00 | Up-regulated |
| 17 | hsa_circ_0049637 | 2.546 | 3.00E-03 | Up-regulated |
| 18 | hsa_circ_0091070 | 2.546 | 0.00E+00 | Up-regulated |
| 19 | hsa_circ_0000511 | 2.541 | 2.00E-03 | Up-regulated |
| 20 | hsa_circ_0066096 | 2.520 | 1.90E-02 | Up-regulated |
| 21 | hsa_circ_0000512 | 2.500 | 3.00E-03 | Up-regulated |
| 22 | hsa_circ_0033572 | 2.493 | 1.00E-03 | Up-regulated |
| 23 | hsa_circ_0000517 | 2.444 | 4.00E-03 | Up-regulated |
| 24 | hsa_circ_0000518 | 2.443 | 7.00E-03 | Up-regulated |
| 25 | hsa_circ_0000742 | 2.441 | 9.00E-03 | Up-regulated |
| 26 | hsa_circ_0065932 | 2.425 | 1.00E-03 | Up-regulated |
| 27 | hsa_circ_0092342 | 2.391 | 2.60E-02 | Up-regulated |
| 28 | hsa_circ_0067185 | 2.373 | 0.00E+00 | Up-regulated |
| 29 | hsa_circ_0000515 | 2.369 | 2.00E-03 | Up-regulated |
| 30 | hsa_circ_0013958 | 2.342 | 6.00E-03 | Up-regulated |
| 31 | hsa_circ_0040507 | 2.322 | 2.00E-03 | Up-regulated |
| 32 | hsa_circ_0003892 | 2.311 | 0.00E+00 | Up-regulated |
| 33 | hsa_circ_0027470 | 2.303 | 7.00E-03 | Up-regulated |
| 34 | hsa_circ_0089763 | 2.294 | 2.00E-03 | Up-regulated |
| 35 | hsa_circ_0041506 | 2.269 | 4.00E-03 | Up-regulated |
| 36 | hsa_circ_0050867 | 2.262 | 1.00E-03 | Up-regulated |
| 37 | hsa_circ_0007237 | 2.250 | 1.00E-03 | Up-regulated |
| 38 | hsa_circ_0036113 | 2.241 | 1.30E-02 | Up-regulated |
| 39 | hsa_circ_0000519 | 2.240 | 2.00E-03 | Up-regulated |
| 40 | hsa_circ_0004074 | 2.237 | 4.00E-03 | Up-regulated |
| 41 | hsa_circ_0000514 | 2.211 | 1.00E-03 | Up-regulated |
| 42 | hsa_circ_0001605 | 2.209 | 9.00E-03 | Up-regulated |
| 43 | hsa_circ_0011692 | 2.193 | 1.00E-03 | Up-regulated |
| 44 | hsa_circ_0035385 | 2.183 | 0.00E+00 | Up-regulated |
| 45 | hsa_circ_0068401 | 2.182 | 2.20E-02 | Up-regulated |
| 46 | hsa_circ_0066113 | 2.137 | 1.00E-03 | Up-regulated |
| 47 | hsa_circ_0001876 | 2.130 | 2.00E-03 | Up-regulated |
| 48 | hsa_circ_0000706 | 2.129 | 1.00E-03 | Up-regulated |
| 49 | hsa_circ_0046419 | 2.123 | 8.00E-03 | Up-regulated |
| 50 | hsa_circ_0092360 | 2.107 | 7.00E-03 | Up-regulated |
| 51 | hsa_circ_0000708 | 2.090 | 9.00E-03 | Up-regulated |
| 52 | hsa_circ_0000026 | 2.085 | 3.00E-03 | Up-regulated |
| 53 | hsa_circ_0073620 | 2.082 | 2.90E-02 | Up-regulated |
| 54 | hsa_circ_0031448 | 2.077 | 6.00E-03 | Up-regulated |
| 55 | hsa_circ_0089252 | 2.064 | 0.00E+00 | Up-regulated |
| 56 | hsa_circ_0040922 | 2.034 | 4.50E-02 | Up-regulated |
| 57 | hsa_circ_0000480 | 2.032 | 1.90E-02 | Up-regulated |
| 58 | hsa_circ_0082326 | 2.032 | 4.30E-02 | Up-regulated |
| 59 | hsa_circ_0077693 | 2.017 | 2.00E-03 | Up-regulated |
| 60 | hsa_circ_0004855 | 2.015 | 1.80E-02 | Up-regulated |
| 61 | hsa_circ_0092353 | 2.012 | 2.00E-02 | Up-regulated |
| 62 | hsa_circ_0050395 | 1.997 | 1.00E-03 | Up-regulated |
| 63 | hsa_circ_0076260 | 1.993 | 2.00E-03 | Up-regulated |
| 64 | hsa_circ_0005108 | 1.991 | 1.00E-03 | Up-regulated |
| 65 | hsa_circ_0086567 | 1.989 | 1.10E-02 | Up-regulated |
| 66 | hsa_circ_0001257 | 1.975 | 1.50E-02 | Up-regulated |
| 67 | hsa_circ_0089254 | 1.963 | 1.00E-03 | Up-regulated |
| 68 | hsa_circ_0015278 | 1.948 | 1.10E-02 | Up-regulated |
| 69 | hsa_circ_0038632 | 1.922 | 1.00E-03 | Up-regulated |
| 70 | hsa_circ_0060876 | 1.881 | 5.00E-03 | Up-regulated |
| 71 | hsa_circ_0069317 | 1.867 | 2.40E-02 | Up-regulated |
| 72 | hsa_circ_0019224 | 1.865 | 1.40E-02 | Up-regulated |
| 73 | hsa_circ_0006296 | 1.860 | 1.10E-02 | Up-regulated |
| 74 | hsa_circ_0007215 | 1.858 | 3.00E-03 | Up-regulated |
| 75 | hsa_circ_0051671 | 1.853 | 1.00E-03 | Up-regulated |
| 76 | hsa_circ_0005272 | 1.852 | 3.00E-03 | Up-regulated |
| 77 | hsa_circ_0016598 | 1.850 | 2.60E-02 | Up-regulated |
| 78 | hsa_circ_0077216 | 1.846 | 1.00E-02 | Up-regulated |
| 79 | hsa_circ_0005531 | 1.842 | 5.00E-03 | Up-regulated |
| 80 | hsa_circ_0083776 | 1.819 | 0.00E+00 | Up-regulated |
| 81 | hsa_circ_0026462 | 1.808 | 2.70E-02 | Up-regulated |
| 82 | hsa_circ_0092355 | 1.790 | 7.00E-03 | Up-regulated |
| 83 | hsa_circ_0033163 | 1.783 | 3.00E-03 | Up-regulated |
| 84 | hsa_circ_0074944 | 1.773 | 7.00E-03 | Up-regulated |
| 85 | hsa_circ_0001204 | 1.771 | 4.00E-03 | Up-regulated |
| 86 | hsa_circ_0079712 | 1.769 | 1.00E-02 | Up-regulated |
| 87 | hsa_circ_0068563 | 1.762 | 1.30E-02 | Up-regulated |
| 88 | hsa_circ_0000930 | 1.758 | 4.00E-03 | Up-regulated |
| 89 | hsa_circ_0008132 | 1.755 | 7.00E-03 | Up-regulated |
| 90 | hsa_circ_0077527 | 1.739 | 2.00E-03 | Up-regulated |
| 91 | hsa_circ_0006847 | 1.720 | 1.00E-03 | Up-regulated |
| 92 | hsa_circ_0009017 | 1.711 | 5.00E-03 | Up-regulated |
| 93 | hsa_circ_0000788 | 1.708 | 2.00E-03 | Up-regulated |
| 94 | hsa_circ_0001015 | 1.704 | 1.00E-02 | Up-regulated |
| 95 | hsa_circ_0092299 | 1.704 | 1.70E-02 | Up-regulated |
| 96 | hsa_circ_0041446 | 1.700 | 0.00E+00 | Up-regulated |
| 97 | hsa_circ_0021019 | 1.685 | 1.00E-03 | Up-regulated |
| 98 | hsa_circ_0046435 | 1.678 | 1.00E-03 | Up-regulated |
| 99 | hsa_circ_0001655 | 1.677 | 6.00E-03 | Up-regulated |
| 100 | hsa_circ_0061829 | 1.674 | 4.00E-03 | Up-regulated |
| 101 | hsa_circ_0057139 | 1.673 | 5.00E-03 | Up-regulated |
| 102 | hsa_circ_0050649 | 1.662 | 7.00E-03 | Up-regulated |
| 103 | hsa_circ_0018004 | 1.655 | 0.00E+00 | Up-regulated |
| 104 | hsa_circ_0007850 | 1.649 | 3.50E-02 | Up-regulated |
| 105 | hsa_circ_0006773 | 1.627 | 1.10E-02 | Up-regulated |
| 106 | hsa_circ_0008584 | 1.622 | 2.00E-03 | Up-regulated |
| 107 | hsa_circ_0000223 | 1.621 | 1.20E-02 | Up-regulated |
| 108 | hsa_circ_0007788 | 1.620 | 5.00E-03 | Up-regulated |
| 109 | hsa_circ_0045881 | 1.620 | 8.00E-03 | Up-regulated |
| 110 | hsa_circ_0003096 | 1.618 | 4.00E-03 | Up-regulated |
| 111 | hsa_circ_0001197 | 1.614 | 2.00E-03 | Up-regulated |
| 112 | hsa_circ_0005075 | 1.607 | 2.00E-03 | Up-regulated |
| 113 | hsa_circ_0003785 | 1.606 | 1.00E-03 | Up-regulated |
| 114 | hsa_circ_0004646 | 1.605 | 4.00E-03 | Up-regulated |
| 115 | hsa_circ_0045788 | 1.602 | 1.10E-02 | Up-regulated |
| 116 | hsa_circ_0008508 | 1.592 | 8.00E-03 | Up-regulated |
| 117 | hsa_circ_0026616 | 1.591 | 1.30E-02 | Up-regulated |
| 118 | hsa_circ_0000585 | 1.589 | 1.00E-02 | Up-regulated |
| 119 | hsa_circ_0067159 | 1.586 | 3.00E-03 | Up-regulated |
| 120 | hsa_circ_0070054 | 1.585 | 6.00E-03 | Up-regulated |
| 121 | hsa_circ_0000735 | 1.584 | 1.00E-03 | Up-regulated |
| 122 | hsa_circ_0000273 | 1.581 | 3.00E-03 | Up-regulated |
| 123 | hsa_circ_0007158 | 1.577 | 7.00E-03 | Up-regulated |
| 124 | hsa_circ_0080968 | 1.573 | 0.00E+00 | Up-regulated |
| 125 | hsa_circ_0009172 | 1.566 | 5.00E-03 | Up-regulated |
| 126 | hsa_circ_0007217 | 1.561 | 2.00E-03 | Up-regulated |
| 127 | hsa_circ_0007221 | 1.559 | 3.00E-03 | Up-regulated |
| 128 | hsa_circ_0005019 | 1.543 | 6.00E-03 | Up-regulated |
| 129 | hsa_circ_0000710 | 1.541 | 1.90E-02 | Up-regulated |
| 130 | hsa_circ_0001177 | 1.538 | 1.20E-02 | Up-regulated |
| 131 | hsa_circ_0063603 | 1.536 | 2.00E-03 | Up-regulated |
| 132 | hsa_circ_0008842 | 1.535 | 1.20E-02 | Up-regulated |
| 133 | hsa_circ_0087040 | 1.531 | 3.00E-03 | Up-regulated |
| 134 | hsa_circ_0010423 | 1.528 | 2.00E-03 | Up-regulated |
| 135 | hsa_circ_0051778 | 1.527 | 2.60E-02 | Up-regulated |
| 136 | hsa_circ_0025858 | 1.527 | 1.00E-03 | Up-regulated |
| 137 | hsa_circ_0058537 | 1.517 | 2.30E-02 | Up-regulated |
| 138 | hsa_circ_0023990 | 1.514 | 1.20E-02 | Up-regulated |
| 139 | hsa_circ_0039908 | 1.512 | 0.00E+00 | Up-regulated |
| 140 | hsa_circ_0016470 | 1.511 | 7.00E-03 | Up-regulated |
| 141 | hsa_circ_0007548 | 1.510 | 2.30E-02 | Up-regulated |
| 142 | hsa_circ_0001208 | 1.509 | 2.00E-03 | Up-regulated |
| 143 | hsa_circ_0048492 | 1.507 | 1.00E-03 | Up-regulated |
| 144 | hsa_circ_0023016 | 1.506 | 1.50E-02 | Up-regulated |
| 145 | hsa_circ_0063072 | 1.505 | 1.80E-02 | Up-regulated |
| 146 | hsa_circ_0003266 | 1.499 | 4.00E-03 | Up-regulated |
| 147 | hsa_circ_0028332 | 1.497 | 1.30E-02 | Up-regulated |
| 148 | hsa_circ_0000395 | 1.486 | 2.60E-02 | Up-regulated |
| 149 | hsa_circ_0064288 | 1.485 | 1.30E-02 | Up-regulated |
| 150 | hsa_circ_0061817 | 1.483 | 8.00E-03 | Up-regulated |
| 151 | hsa_circ_0000520 | 1.482 | 1.20E-02 | Up-regulated |
| 152 | hsa_circ_0035277 | 1.479 | 4.20E-02 | Up-regulated |
| 153 | hsa_circ_0079385 | 1.476 | 2.30E-02 | Up-regulated |
| 154 | hsa_circ_0000700 | 1.473 | 0.00E+00 | Up-regulated |
| 155 | hsa_circ_0079972 | 1.471 | 3.60E-02 | Up-regulated |
| 156 | hsa_circ_0005086 | 1.469 | 1.40E-02 | Up-regulated |
| 157 | hsa_circ_0003494 | 1.466 | 0.00E+00 | Up-regulated |
| 158 | hsa_circ_0065215 | 1.464 | 3.00E-03 | Up-regulated |
| 159 | hsa_circ_0002453 | 1.460 | 1.00E-03 | Up-regulated |
| 160 | hsa_circ_0063678 | 1.458 | 1.00E-03 | Up-regulated |
| 161 | hsa_circ_0045556 | 1.458 | 0.00E+00 | Up-regulated |
| 162 | hsa_circ_0047546 | 1.457 | 0.00E+00 | Up-regulated |
| 163 | hsa_circ_0051036 | 1.456 | 0.00E+00 | Up-regulated |
| 164 | hsa_circ_0001296 | 1.451 | 2.00E-02 | Up-regulated |
| 165 | hsa_circ_0000435 | 1.450 | 6.00E-03 | Up-regulated |
| 166 | hsa_circ_0066842 | 1.450 | 4.00E-03 | Up-regulated |
| 167 | hsa_circ_0091722 | 1.449 | 3.00E-03 | Up-regulated |
| 168 | hsa_circ_0091186 | 1.440 | 3.40E-02 | Up-regulated |
| 169 | hsa_circ_0036691 | 1.433 | 3.80E-02 | Up-regulated |
| 170 | hsa_circ_0038844 | 1.423 | 2.80E-02 | Up-regulated |
| 171 | hsa_circ_0080638 | 1.416 | 0.00E+00 | Up-regulated |
| 172 | hsa_circ_0002824 | 1.410 | 3.50E-02 | Up-regulated |
| 173 | hsa_circ_0068669 | 1.409 | 4.50E-02 | Up-regulated |
| 174 | hsa_circ_0001730 | 1.408 | 7.00E-03 | Up-regulated |
| 175 | hsa_circ_0004896 | 1.407 | 1.70E-02 | Up-regulated |
| 176 | hsa_circ_0019272 | 1.402 | 3.80E-02 | Up-regulated |
| 177 | hsa_circ_0053944 | 1.397 | 3.10E-02 | Up-regulated |
| 178 | hsa_circ_0047301 | 1.394 | 0.00E+00 | Up-regulated |
| 179 | hsa_circ_0073009 | 1.390 | 1.20E-02 | Up-regulated |
| 180 | hsa_circ_0020934 | 1.381 | 2.00E-03 | Up-regulated |
| 181 | hsa_circ_0003748 | 1.378 | 4.80E-02 | Up-regulated |
| 182 | hsa_circ_0092310 | 1.373 | 3.10E-02 | Up-regulated |
| 183 | hsa_circ_0000407 | 1.368 | 6.00E-03 | Up-regulated |
| 184 | hsa_circ_0090650 | 1.357 | 4.60E-02 | Up-regulated |
| 185 | hsa_circ_0036205 | 1.352 | 1.00E-03 | Up-regulated |
| 186 | hsa_circ_0020929 | 1.345 | 1.00E-02 | Up-regulated |
| 187 | hsa_circ_0073695 | 1.341 | 2.00E-02 | Up-regulated |
| 188 | hsa_circ_0091223 | 1.335 | 2.00E-03 | Up-regulated |
| 189 | hsa_circ_0003478 | 1.324 | 3.00E-03 | Up-regulated |
| 190 | hsa_circ_0007099 | 1.324 | 4.00E-03 | Up-regulated |
| 191 | hsa_circ_0058957 | 1.322 | 6.00E-03 | Up-regulated |
| 192 | hsa_circ_0045355 | 1.319 | 1.90E-02 | Up-regulated |
| 193 | hsa_circ_0092332 | 1.316 | 9.00E-03 | Up-regulated |
| 194 | hsa_circ_0026134 | 1.315 | 0.00E+00 | Up-regulated |
| 195 | hsa_circ_0074909 | 1.315 | 1.10E-02 | Up-regulated |
| 196 | hsa_circ_0006836 | 1.313 | 4.60E-02 | Up-regulated |
| 197 | hsa_circ_0001828 | 1.313 | 1.10E-02 | Up-regulated |
| 198 | hsa_circ_0005616 | 1.310 | 1.00E-03 | Up-regulated |
| 199 | hsa_circ_0030428 | 1.308 | 1.10E-02 | Up-regulated |
| 200 | hsa_circ_0001819 | 1.307 | 5.00E-03 | Up-regulated |
| 201 | hsa_circ_0059629 | 1.306 | 0.00E+00 | Up-regulated |
| 202 | hsa_circ_0006841 | 1.296 | 3.00E-03 | Up-regulated |
| 203 | hsa_circ_0046598 | 1.296 | 1.60E-02 | Up-regulated |
| 204 | hsa_circ_0069397 | 1.293 | 1.00E-03 | Up-regulated |
| 205 | hsa_circ_0009684 | 1.292 | 5.00E-03 | Up-regulated |
| 206 | hsa_circ_0081481 | 1.292 | 2.40E-02 | Up-regulated |
| 207 | hsa_circ_0017330 | 1.291 | 0.00E+00 | Up-regulated |
| 208 | hsa_circ_0001818 | 1.289 | 1.70E-02 | Up-regulated |
| 209 | hsa_circ_0067674 | 1.287 | 0.00E+00 | Up-regulated |
| 210 | hsa_circ_0015546 | 1.287 | 1.70E-02 | Up-regulated |
| 211 | hsa_circ_0012687 | 1.287 | 1.00E-03 | Up-regulated |
| 212 | hsa_circ_0047478 | 1.284 | 5.00E-03 | Up-regulated |
| 213 | hsa_circ_0062980 | 1.282 | 3.10E-02 | Up-regulated |
| 214 | hsa_circ_0000294 | 1.279 | 0.00E+00 | Up-regulated |
| 215 | hsa_circ_0033023 | 1.277 | 3.10E-02 | Up-regulated |
| 216 | hsa_circ_0011977 | 1.273 | 1.00E-02 | Up-regulated |
| 217 | hsa_circ_0077618 | 1.271 | 1.00E-02 | Up-regulated |
| 218 | hsa_circ_0056430 | 1.268 | 1.00E-02 | Up-regulated |
| 219 | hsa_circ_0065214 | 1.264 | 1.00E-03 | Up-regulated |
| 220 | hsa_circ_0035482 | 1.264 | 4.70E-02 | Up-regulated |
| 221 | hsa_circ_0079675 | 1.263 | 1.00E-03 | Up-regulated |
| 222 | hsa_circ_0076507 | 1.261 | 1.30E-02 | Up-regulated |
| 223 | hsa_circ_0057897 | 1.261 | 3.00E-03 | Up-regulated |
| 224 | hsa_circ_0005623 | 1.260 | 4.00E-03 | Up-regulated |
| 225 | hsa_circ_0045368 | 1.260 | 3.00E-03 | Up-regulated |
| 226 | hsa_circ_0039187 | 1.252 | 0.00E+00 | Up-regulated |
| 227 | hsa_circ_0053030 | 1.249 | 2.00E-03 | Up-regulated |
| 228 | hsa_circ_0000979 | 1.246 | 6.00E-03 | Up-regulated |
| 229 | hsa_circ_0045834 | 1.246 | 1.30E-02 | Up-regulated |
| 230 | hsa_circ_0034183 | 1.245 | 1.10E-02 | Up-regulated |
| 231 | hsa_circ_0000751 | 1.238 | 1.00E-03 | Up-regulated |
| 232 | hsa_circ_0005699 | 1.236 | 3.60E-02 | Up-regulated |
| 233 | hsa_circ_0092289 | 1.234 | 3.40E-02 | Up-regulated |
| 234 | hsa_circ_0005957 | 1.224 | 3.40E-02 | Up-regulated |
| 235 | hsa_circ_0071262 | 1.221 | 9.00E-03 | Up-regulated |
| 236 | hsa_circ_0015879 | 1.220 | 1.90E-02 | Up-regulated |
| 237 | hsa_circ_0025522 | 1.219 | 1.50E-02 | Up-regulated |
| 238 | hsa_circ_0039888 | 1.218 | 1.00E-03 | Up-regulated |
| 239 | hsa_circ_0027702 | 1.216 | 1.40E-02 | Up-regulated |
| 240 | hsa_circ_0074216 | 1.216 | 1.00E-03 | Up-regulated |
| 241 | hsa_circ_0008913 | 1.210 | 3.00E-03 | Up-regulated |
| 242 | hsa_circ_0005927 | 1.209 | 1.10E-02 | Up-regulated |
| 243 | hsa_circ_0063419 | 1.209 | 4.40E-02 | Up-regulated |
| 244 | hsa_circ_0007340 | 1.205 | 4.90E-02 | Up-regulated |
| 245 | hsa_circ_0006002 | 1.197 | 1.00E-03 | Up-regulated |
| 246 | hsa_circ_0071774 | 1.195 | 2.00E-03 | Up-regulated |
| 247 | hsa_circ_0083948 | 1.195 | 3.30E-02 | Up-regulated |
| 248 | hsa_circ_0001549 | 1.194 | 7.00E-03 | Up-regulated |
| 249 | hsa_circ_0073158 | 1.193 | 9.00E-03 | Up-regulated |
| 250 | hsa_circ_0092367 | 1.193 | 3.40E-02 | Up-regulated |
| 251 | hsa_circ_0004647 | 1.184 | 4.00E-02 | Up-regulated |
| 252 | hsa_circ_0086760 | 1.180 | 2.60E-02 | Up-regulated |
| 253 | hsa_circ_0027621 | 1.177 | 1.00E-03 | Up-regulated |
| 254 | hsa_circ_0005414 | 1.175 | 1.00E-03 | Up-regulated |
| 255 | hsa_circ_0046422 | 1.175 | 5.00E-03 | Up-regulated |
| 256 | hsa_circ_0000877 | 1.172 | 6.00E-03 | Up-regulated |
| 257 | hsa_circ_0092288 | 1.169 | 1.60E-02 | Up-regulated |
| 258 | hsa_circ_0055547 | 1.168 | 4.60E-02 | Up-regulated |
| 259 | hsa_circ_0034804 | 1.166 | 3.30E-02 | Up-regulated |
| 260 | hsa_circ_0070421 | 1.163 | 1.00E-03 | Up-regulated |
| 261 | hsa_circ_0083759 | 1.162 | 1.90E-02 | Up-regulated |
| 262 | hsa_circ_0000253 | 1.161 | 1.60E-02 | Up-regulated |
| 263 | hsa_circ_0021347 | 1.158 | 8.00E-03 | Up-regulated |
| 264 | hsa_circ_0008039 | 1.157 | 0.00E+00 | Up-regulated |
| 265 | hsa_circ_0081648 | 1.157 | 2.00E-02 | Up-regulated |
| 266 | hsa_circ_0033144 | 1.152 | 3.60E-02 | Up-regulated |
| 267 | hsa_circ_0008189 | 1.147 | 1.60E-02 | Up-regulated |
| 268 | hsa_circ_0087849 | 1.145 | 7.00E-03 | Up-regulated |
| 269 | hsa_circ_0000432 | 1.139 | 7.00E-03 | Up-regulated |
| 270 | hsa_circ_0067301 | 1.139 | 2.00E-03 | Up-regulated |
| 271 | hsa_circ_0081728 | 1.139 | 1.10E-02 | Up-regulated |
| 272 | hsa_circ_0004528 | 1.135 | 2.20E-02 | Up-regulated |
| 273 | hsa_circ_0072088 | 1.131 | 4.00E-03 | Up-regulated |
| 274 | hsa_circ_0000281 | 1.128 | 3.00E-03 | Up-regulated |
| 275 | hsa_circ_0008016 | 1.123 | 2.20E-02 | Up-regulated |
| 276 | hsa_circ_0077526 | 1.122 | 3.00E-02 | Up-regulated |
| 277 | hsa_circ_0005500 | 1.120 | 4.60E-02 | Up-regulated |
| 278 | hsa_circ_0003738 | 1.120 | 3.40E-02 | Up-regulated |
| 279 | hsa_circ_0063226 | 1.119 | 1.40E-02 | Up-regulated |
| 280 | hsa_circ_0007192 | 1.118 | 3.00E-03 | Up-regulated |
| 281 | hsa_circ_0063411 | 1.118 | 3.70E-02 | Up-regulated |
| 282 | hsa_circ_0013058 | 1.116 | 4.00E-03 | Up-regulated |
| 283 | hsa_circ_0063811 | 1.114 | 6.00E-03 | Up-regulated |
| 284 | hsa_circ_0068367 | 1.108 | 2.40E-02 | Up-regulated |
| 285 | hsa_circ_0001357 | 1.108 | 3.00E-03 | Up-regulated |
| 286 | hsa_circ_0039319 | 1.107 | 2.00E-02 | Up-regulated |
| 287 | hsa_circ_0004884 | 1.103 | 4.00E-03 | Up-regulated |
| 288 | hsa_circ_0089761 | 1.101 | 2.90E-02 | Up-regulated |
| 289 | hsa_circ_0004864 | 1.097 | 1.90E-02 | Up-regulated |
| 290 | hsa_circ_0028196 | 1.097 | 3.50E-02 | Up-regulated |
| 291 | hsa_circ_0050898 | 1.090 | 8.00E-03 | Up-regulated |
| 292 | hsa_circ_0060144 | 1.090 | 4.10E-02 | Up-regulated |
| 293 | hsa_circ_0092283 | 1.090 | 4.60E-02 | Up-regulated |
| 294 | hsa_circ_0003497 | 1.089 | 7.00E-03 | Up-regulated |
| 295 | hsa_circ_0008027 | 1.087 | 7.00E-03 | Up-regulated |
| 296 | hsa_circ_0002643 | 1.086 | 1.50E-02 | Up-regulated |
| 297 | hsa_circ_0012634 | 1.081 | 7.00E-03 | Up-regulated |
| 298 | hsa_circ_0005707 | 1.079 | 3.00E-03 | Up-regulated |
| 299 | hsa_circ_0087888 | 1.078 | 0.00E+00 | Up-regulated |
| 300 | hsa_circ_0007928 | 1.076 | 9.00E-03 | Up-regulated |
| 301 | hsa_circ_0003141 | 1.073 | 0.00E+00 | Up-regulated |
| 302 | hsa_circ_0004988 | 1.073 | 1.20E-02 | Up-regulated |
| 303 | hsa_circ_0009012 | 1.072 | 1.30E-02 | Up-regulated |
| 304 | hsa_circ_0039943 | 1.068 | 1.90E-02 | Up-regulated |
| 305 | hsa_circ_0005896 | 1.068 | 1.10E-02 | Up-regulated |
| 306 | hsa_circ_0005881 | 1.066 | 4.10E-02 | Up-regulated |
| 307 | hsa_circ_0092333 | 1.061 | 0.00E+00 | Up-regulated |
| 308 | hsa_circ_0025460 | 1.055 | 4.30E-02 | Up-regulated |
| 309 | hsa_circ_0007586 | 1.054 | 0.00E+00 | Up-regulated |
| 310 | hsa_circ_0052165 | 1.049 | 5.00E-03 | Up-regulated |
| 311 | hsa_circ_0019774 | 1.048 | 1.80E-02 | Up-regulated |
| 312 | hsa_circ_0088510 | 1.046 | 1.00E-02 | Up-regulated |
| 313 | hsa_circ_0052300 | 1.044 | 4.00E-03 | Up-regulated |
| 314 | hsa_circ_0001618 | 1.044 | 3.30E-02 | Up-regulated |
| 315 | hsa_circ_0007833 | 1.043 | 1.00E-03 | Up-regulated |
| 316 | hsa_circ_0038959 | 1.040 | 1.00E-03 | Up-regulated |
| 317 | hsa_circ_0001171 | 1.038 | 0.00E+00 | Up-regulated |
| 318 | hsa_circ_0020080 | 1.035 | 5.00E-03 | Up-regulated |
| 319 | hsa_circ_0068772 | 1.035 | 4.10E-02 | Up-regulated |
| 320 | hsa_circ_0006055 | 1.031 | 9.00E-03 | Up-regulated |
| 321 | hsa_circ_0034447 | 1.031 | 6.00E-03 | Up-regulated |
| 322 | hsa_circ_0003945 | 1.029 | 4.30E-02 | Up-regulated |
| 323 | hsa_circ_0034833 | 1.028 | 3.30E-02 | Up-regulated |
| 324 | hsa_circ_0007597 | 1.027 | 3.70E-02 | Up-regulated |
| 325 | hsa_circ_0002131 | 1.025 | 3.70E-02 | Up-regulated |
| 326 | hsa_circ_0008183 | 1.021 | 4.90E-02 | Up-regulated |
| 327 | hsa_circ_0021529 | 1.018 | 4.40E-02 | Up-regulated |
| 328 | hsa_circ_0085952 | 1.018 | 2.00E-03 | Up-regulated |
| 329 | hsa_circ_0012125 | 1.018 | 3.00E-03 | Up-regulated |
| 330 | hsa_circ_0071016 | 1.018 | 1.00E-03 | Up-regulated |
| 331 | hsa_circ_0069086 | 1.017 | 0.00E+00 | Up-regulated |
| 332 | hsa_circ_0006909 | 1.014 | 9.00E-03 | Up-regulated |
| 333 | hsa_circ_0007874 | 1.011 | 7.00E-03 | Up-regulated |
| 334 | hsa_circ_0006187 | 1.009 | 2.00E-03 | Up-regulated |
| 335 | hsa_circ_0066380 | 1.007 | 1.00E-02 | Up-regulated |
| 336 | hsa_circ_0074136 | 1.004 | 1.00E-03 | Up-regulated |
| 337 | hsa_circ_0058495 | 1.002 | 3.00E-03 | Up-regulated |
| 338 | hsa_circ_0089851 | 1.002 | 1.60E-02 | Up-regulated |
| 339 | hsa_circ_0001489 | 1.002 | 1.20E-02 | Up-regulated |
| 340 | hsa_circ_0005204 | 1.001 | 3.00E-03 | Up-regulated |
| 341 | hsa_circ_0004002 | -1.000 | 3.10E-02 | Down-regulated |
| 342 | hsa_circ_0001110 | -1.001 | 2.60E-02 | Down-regulated |
| 343 | hsa_circ_0067200 | -1.002 | 4.40E-02 | Down-regulated |
| 344 | hsa_circ_0004608 | -1.002 | 2.30E-02 | Down-regulated |
| 345 | hsa_circ_0008773 | -1.002 | 2.30E-02 | Down-regulated |
| 346 | hsa_circ_0038348 | -1.002 | 4.30E-02 | Down-regulated |
| 347 | hsa_circ_0036320 | -1.003 | 3.10E-02 | Down-regulated |
| 348 | hsa_circ_0005523 | -1.003 | 1.30E-02 | Down-regulated |
| 349 | hsa_circ_0005749 | -1.004 | 1.00E-02 | Down-regulated |
| 350 | hsa_circ_0035944 | -1.004 | 4.80E-02 | Down-regulated |
| 351 | hsa_circ_0090142 | -1.004 | 4.10E-02 | Down-regulated |
| 352 | hsa_circ_0025359 | -1.004 | 4.40E-02 | Down-regulated |
| 353 | hsa_circ_0038480 | -1.004 | 3.50E-02 | Down-regulated |
| 354 | hsa_circ_0007275 | -1.005 | 3.30E-02 | Down-regulated |
| 355 | hsa_circ_0025489 | -1.005 | 3.20E-02 | Down-regulated |
| 356 | hsa_circ_0039253 | -1.005 | 3.40E-02 | Down-regulated |
| 357 | hsa_circ_0006146 | -1.006 | 2.20E-02 | Down-regulated |
| 358 | hsa_circ_0007319 | -1.006 | 3.70E-02 | Down-regulated |
| 359 | hsa_circ_0015169 | -1.006 | 2.30E-02 | Down-regulated |
| 360 | hsa_circ_0003255 | -1.006 | 4.00E-02 | Down-regulated |
| 361 | hsa_circ_0007587 | -1.007 | 3.80E-02 | Down-regulated |
| 362 | hsa_circ_0000192 | -1.007 | 3.30E-02 | Down-regulated |
| 363 | hsa_circ_0052712 | -1.008 | 1.60E-02 | Down-regulated |
| 364 | hsa_circ_0025967 | -1.009 | 3.00E-02 | Down-regulated |
| 365 | hsa_circ_0054498 | -1.009 | 1.70E-02 | Down-regulated |
| 366 | hsa_circ_0040366 | -1.009 | 1.20E-02 | Down-regulated |
| 367 | hsa_circ_0006970 | -1.009 | 1.40E-02 | Down-regulated |
| 368 | hsa_circ_0006618 | -1.010 | 2.60E-02 | Down-regulated |
| 369 | hsa_circ_0005210 | -1.011 | 4.60E-02 | Down-regulated |
| 370 | hsa_circ_0057906 | -1.012 | 4.80E-02 | Down-regulated |
| 371 | hsa_circ_0023904 | -1.012 | 4.40E-02 | Down-regulated |
| 372 | hsa_circ_0027914 | -1.013 | 4.00E-02 | Down-regulated |
| 373 | hsa_circ_0091743 | -1.014 | 1.80E-02 | Down-regulated |
| 374 | hsa_circ_0005955 | -1.014 | 3.80E-02 | Down-regulated |
| 375 | hsa_circ_0000304 | -1.015 | 4.00E-02 | Down-regulated |
| 376 | hsa_circ_0067809 | -1.015 | 4.00E-02 | Down-regulated |
| 377 | hsa_circ_0046909 | -1.016 | 4.10E-02 | Down-regulated |
| 378 | hsa_circ_0008654 | -1.016 | 1.90E-02 | Down-regulated |
| 379 | hsa_circ_0004273 | -1.016 | 4.70E-02 | Down-regulated |
| 380 | hsa_circ_0086735 | -1.016 | 4.20E-02 | Down-regulated |
| 381 | hsa_circ_0005755 | -1.016 | 3.60E-02 | Down-regulated |
| 382 | hsa_circ_0083390 | -1.017 | 4.20E-02 | Down-regulated |
| 383 | hsa_circ_0012366 | -1.017 | 2.60E-02 | Down-regulated |
| 384 | hsa_circ_0008087 | -1.018 | 7.00E-03 | Down-regulated |
| 385 | hsa_circ_0046660 | -1.018 | 3.00E-02 | Down-regulated |
| 386 | hsa_circ_0026523 | -1.018 | 2.10E-02 | Down-regulated |
| 387 | hsa_circ_0000070 | -1.018 | 3.00E-02 | Down-regulated |
| 388 | hsa_circ_0008762 | -1.018 | 3.50E-02 | Down-regulated |
| 389 | hsa_circ_0006646 | -1.019 | 4.40E-02 | Down-regulated |
| 390 | hsa_circ_0066568 | -1.019 | 3.40E-02 | Down-regulated |
| 391 | hsa_circ_0074269 | -1.020 | 3.20E-02 | Down-regulated |
| 392 | hsa_circ_0026787 | -1.020 | 2.80E-02 | Down-regulated |
| 393 | hsa_circ_0004964 | -1.020 | 2.70E-02 | Down-regulated |
| 394 | hsa_circ_0058793 | -1.020 | 3.00E-02 | Down-regulated |
| 395 | hsa_circ_0003316 | -1.022 | 2.80E-02 | Down-regulated |
| 396 | hsa_circ_0011264 | -1.022 | 1.10E-02 | Down-regulated |
| 397 | hsa_circ_0003400 | -1.022 | 2.90E-02 | Down-regulated |
| 398 | hsa_circ_0090162 | -1.022 | 2.30E-02 | Down-regulated |
| 399 | hsa_circ_0069382 | -1.023 | 4.20E-02 | Down-regulated |
| 400 | hsa_circ_0073859 | -1.024 | 3.70E-02 | Down-regulated |
| 401 | hsa_circ_0000926 | -1.024 | 3.60E-02 | Down-regulated |
| 402 | hsa_circ_0047003 | -1.024 | 3.70E-02 | Down-regulated |
| 403 | hsa_circ_0068656 | -1.025 | 2.30E-02 | Down-regulated |
| 404 | hsa_circ_0036380 | -1.026 | 2.90E-02 | Down-regulated |
| 405 | hsa_circ_0002620 | -1.026 | 6.00E-03 | Down-regulated |
| 406 | hsa_circ_0025699 | -1.026 | 2.70E-02 | Down-regulated |
| 407 | hsa_circ_0001629 | -1.026 | 1.80E-02 | Down-regulated |
| 408 | hsa_circ_0040704 | -1.027 | 3.80E-02 | Down-regulated |
| 409 | hsa_circ_0029104 | -1.027 | 1.50E-02 | Down-regulated |
| 410 | hsa_circ_0001058 | -1.028 | 4.70E-02 | Down-regulated |
| 411 | hsa_circ_0091398 | -1.028 | 2.00E-02 | Down-regulated |
| 412 | hsa_circ_0044737 | -1.028 | 3.60E-02 | Down-regulated |
| 413 | hsa_circ_0000159 | -1.028 | 1.80E-02 | Down-regulated |
| 414 | hsa_circ_0060633 | -1.028 | 4.90E-02 | Down-regulated |
| 415 | hsa_circ_0006386 | -1.029 | 2.20E-02 | Down-regulated |
| 416 | hsa_circ_0024748 | -1.030 | 4.20E-02 | Down-regulated |
| 417 | hsa_circ_0018148 | -1.031 | 1.10E-02 | Down-regulated |
| 418 | hsa_circ_0026066 | -1.031 | 1.80E-02 | Down-regulated |
| 419 | hsa_circ_0022074 | -1.032 | 2.50E-02 | Down-regulated |
| 420 | hsa_circ_0079573 | -1.032 | 2.60E-02 | Down-regulated |
| 421 | hsa_circ_0066803 | -1.034 | 3.60E-02 | Down-regulated |
| 422 | hsa_circ_0086475 | -1.034 | 2.90E-02 | Down-regulated |
| 423 | hsa_circ_0005286 | -1.035 | 1.20E-02 | Down-regulated |
| 424 | hsa_circ_0032777 | -1.035 | 2.70E-02 | Down-regulated |
| 425 | hsa_circ_0031429 | -1.035 | 4.60E-02 | Down-regulated |
| 426 | hsa_circ_0008763 | -1.035 | 2.50E-02 | Down-regulated |
| 427 | hsa_circ_0007966 | -1.036 | 2.90E-02 | Down-regulated |
| 428 | hsa_circ_0052612 | -1.036 | 3.60E-02 | Down-regulated |
| 429 | hsa_circ_0046953 | -1.036 | 2.30E-02 | Down-regulated |
| 430 | hsa_circ_0004453 | -1.037 | 3.00E-02 | Down-regulated |
| 431 | hsa_circ_0050518 | -1.037 | 3.60E-02 | Down-regulated |
| 432 | hsa_circ_0013367 | -1.037 | 7.00E-03 | Down-regulated |
| 433 | hsa_circ_0014188 | -1.037 | 4.00E-02 | Down-regulated |
| 434 | hsa_circ_0000045 | -1.037 | 4.50E-02 | Down-regulated |
| 435 | hsa_circ_0008405 | -1.037 | 3.80E-02 | Down-regulated |
| 436 | hsa_circ_0021570 | -1.037 | 2.70E-02 | Down-regulated |
| 437 | hsa_circ_0000982 | -1.038 | 2.20E-02 | Down-regulated |
| 438 | hsa_circ_0084499 | -1.038 | 1.40E-02 | Down-regulated |
| 439 | hsa_circ_0029646 | -1.038 | 1.20E-02 | Down-regulated |
| 440 | hsa_circ_0087570 | -1.039 | 3.80E-02 | Down-regulated |
| 441 | hsa_circ_0019005 | -1.039 | 3.00E-02 | Down-regulated |
| 442 | hsa_circ_0002186 | -1.040 | 4.50E-02 | Down-regulated |
| 443 | hsa_circ_0032682 | -1.041 | 4.90E-02 | Down-regulated |
| 444 | hsa_circ_0086205 | -1.041 | 4.60E-02 | Down-regulated |
| 445 | hsa_circ_0017972 | -1.041 | 3.20E-02 | Down-regulated |
| 446 | hsa_circ_0001311 | -1.041 | 4.00E-02 | Down-regulated |
| 447 | hsa_circ_0087220 | -1.041 | 2.90E-02 | Down-regulated |
| 448 | hsa_circ_0003022 | -1.042 | 3.30E-02 | Down-regulated |
| 449 | hsa_circ_0000836 | -1.042 | 3.40E-02 | Down-regulated |
| 450 | hsa_circ_0001494 | -1.042 | 3.80E-02 | Down-regulated |
| 451 | hsa_circ_0005394 | -1.043 | 3.60E-02 | Down-regulated |
| 452 | hsa_circ_0056586 | -1.043 | 1.60E-02 | Down-regulated |
| 453 | hsa_circ_0066642 | -1.043 | 4.20E-02 | Down-regulated |
| 454 | hsa_circ_0006211 | -1.044 | 1.90E-02 | Down-regulated |
| 455 | hsa_circ_0074543 | -1.044 | 4.00E-02 | Down-regulated |
| 456 | hsa_circ_0055954 | -1.044 | 2.20E-02 | Down-regulated |
| 457 | hsa_circ_0044646 | -1.044 | 2.80E-02 | Down-regulated |
| 458 | hsa_circ_0003692 | -1.045 | 2.00E-02 | Down-regulated |
| 459 | hsa_circ_0043140 | -1.045 | 2.40E-02 | Down-regulated |
| 460 | hsa_circ_0006810 | -1.045 | 1.70E-02 | Down-regulated |
| 461 | hsa_circ_0075437 | -1.045 | 3.50E-02 | Down-regulated |
| 462 | hsa_circ_0008137 | -1.046 | 1.90E-02 | Down-regulated |
| 463 | hsa_circ_0057827 | -1.047 | 3.10E-02 | Down-regulated |
| 464 | hsa_circ_0004300 | -1.047 | 2.30E-02 | Down-regulated |
| 465 | hsa_circ_0031830 | -1.048 | 1.60E-02 | Down-regulated |
| 466 | hsa_circ_0042176 | -1.048 | 1.90E-02 | Down-regulated |
| 467 | hsa_circ_0007986 | -1.048 | 3.50E-02 | Down-regulated |
| 468 | hsa_circ_0030327 | -1.048 | 3.20E-02 | Down-regulated |
| 469 | hsa_circ_0001859 | -1.049 | 4.90E-02 | Down-regulated |
| 470 | hsa_circ_0013162 | -1.049 | 1.20E-02 | Down-regulated |
| 471 | hsa_circ_0002747 | -1.050 | 1.90E-02 | Down-regulated |
| 472 | hsa_circ_0003574 | -1.050 | 1.00E-02 | Down-regulated |
| 473 | hsa_circ_0025853 | -1.051 | 3.20E-02 | Down-regulated |
| 474 | hsa_circ_0072309 | -1.051 | 3.10E-02 | Down-regulated |
| 475 | hsa_circ_0001315 | -1.051 | 1.50E-02 | Down-regulated |
| 476 | hsa_circ_0008720 | -1.051 | 2.60E-02 | Down-regulated |
| 477 | hsa_circ_0043537 | -1.052 | 2.60E-02 | Down-regulated |
| 478 | hsa_circ_0005367 | -1.052 | 1.20E-02 | Down-regulated |
| 479 | hsa_circ_0030681 | -1.052 | 2.20E-02 | Down-regulated |
| 480 | hsa_circ_0005490 | -1.052 | 3.20E-02 | Down-regulated |
| 481 | hsa_circ_0004344 | -1.052 | 2.50E-02 | Down-regulated |
| 482 | hsa_circ_0057684 | -1.053 | 2.90E-02 | Down-regulated |
| 483 | hsa_circ_0019565 | -1.053 | 2.80E-02 | Down-regulated |
| 484 | hsa_circ_0008404 | -1.053 | 1.60E-02 | Down-regulated |
| 485 | hsa_circ_0085362 | -1.053 | 1.90E-02 | Down-regulated |
| 486 | hsa_circ_0066640 | -1.053 | 3.40E-02 | Down-regulated |
| 487 | hsa_circ_0089629 | -1.054 | 1.30E-02 | Down-regulated |
| 488 | hsa_circ_0014405 | -1.054 | 1.90E-02 | Down-regulated |
| 489 | hsa_circ_0001351 | -1.054 | 3.00E-02 | Down-regulated |
| 490 | hsa_circ_0005693 | -1.055 | 3.20E-02 | Down-regulated |
| 491 | hsa_circ_0005852 | -1.055 | 3.10E-02 | Down-regulated |
| 492 | hsa_circ_0009160 | -1.056 | 9.00E-03 | Down-regulated |
| 493 | hsa_circ_0084149 | -1.056 | 3.30E-02 | Down-regulated |
| 494 | hsa_circ_0074927 | -1.056 | 1.70E-02 | Down-regulated |
| 495 | hsa_circ_0005941 | -1.058 | 1.50E-02 | Down-regulated |
| 496 | hsa_circ_0050511 | -1.058 | 4.00E-02 | Down-regulated |
| 497 | hsa_circ_0000501 | -1.059 | 1.10E-02 | Down-regulated |
| 498 | hsa_circ_0005227 | -1.060 | 3.30E-02 | Down-regulated |
| 499 | hsa_circ_0023612 | -1.060 | 1.40E-02 | Down-regulated |
| 500 | hsa_circ_0081984 | -1.060 | 9.00E-03 | Down-regulated |
| 501 | hsa_circ_0000636 | -1.060 | 4.60E-02 | Down-regulated |
| 502 | hsa_circ_0000164 | -1.061 | 1.90E-02 | Down-regulated |
| 503 | hsa_circ_0023836 | -1.061 | 4.40E-02 | Down-regulated |
| 504 | hsa_circ_0012332 | -1.061 | 4.20E-02 | Down-regulated |
| 505 | hsa_circ_0037002 | -1.061 | 1.70E-02 | Down-regulated |
| 506 | hsa_circ_0052054 | -1.062 | 2.70E-02 | Down-regulated |
| 507 | hsa_circ_0001472 | -1.063 | 3.10E-02 | Down-regulated |
| 508 | hsa_circ_0017803 | -1.063 | 6.00E-03 | Down-regulated |
| 509 | hsa_circ_0001454 | -1.063 | 2.70E-02 | Down-regulated |
| 510 | hsa_circ_0054033 | -1.063 | 3.50E-02 | Down-regulated |
| 511 | hsa_circ_0013573 | -1.063 | 4.50E-02 | Down-regulated |
| 512 | hsa_circ_0091024 | -1.064 | 2.00E-02 | Down-regulated |
| 513 | hsa_circ_0000543 | -1.065 | 1.60E-02 | Down-regulated |
| 514 | hsa_circ_0007614 | -1.065 | 1.60E-02 | Down-regulated |
| 515 | hsa_circ_0005879 | -1.065 | 3.60E-02 | Down-regulated |
| 516 | hsa_circ_0000780 | -1.066 | 2.90E-02 | Down-regulated |
| 517 | hsa_circ_0091725 | -1.066 | 3.00E-03 | Down-regulated |
| 518 | hsa_circ_0020398 | -1.066 | 1.80E-02 | Down-regulated |
| 519 | hsa_circ_0050516 | -1.066 | 3.00E-02 | Down-regulated |
| 520 | hsa_circ_0056090 | -1.067 | 2.90E-02 | Down-regulated |
| 521 | hsa_circ_0000756 | -1.067 | 3.70E-02 | Down-regulated |
| 522 | hsa_circ_0002513 | -1.068 | 5.00E-02 | Down-regulated |
| 523 | hsa_circ_0049055 | -1.068 | 3.00E-02 | Down-regulated |
| 524 | hsa_circ_0000260 | -1.069 | 6.00E-03 | Down-regulated |
| 525 | hsa_circ_0008702 | -1.069 | 4.00E-02 | Down-regulated |
| 526 | hsa_circ_0006018 | -1.069 | 4.60E-02 | Down-regulated |
| 527 | hsa_circ_0059490 | -1.069 | 3.10E-02 | Down-regulated |
| 528 | hsa_circ_0000224 | -1.070 | 2.20E-02 | Down-regulated |
| 529 | hsa_circ_0023923 | -1.071 | 4.60E-02 | Down-regulated |
| 530 | hsa_circ_0039254 | -1.071 | 2.30E-02 | Down-regulated |
| 531 | hsa_circ_0006610 | -1.071 | 3.70E-02 | Down-regulated |
| 532 | hsa_circ_0015579 | -1.073 | 3.60E-02 | Down-regulated |
| 533 | hsa_circ_0003602 | -1.073 | 1.10E-02 | Down-regulated |
| 534 | hsa_circ_0008213 | -1.073 | 6.00E-03 | Down-regulated |
| 535 | hsa_circ_0002692 | -1.073 | 4.60E-02 | Down-regulated |
| 536 | hsa_circ_0004345 | -1.074 | 3.30E-02 | Down-regulated |
| 537 | hsa_circ_0004802 | -1.074 | 1.20E-02 | Down-regulated |
| 538 | hsa_circ_0054663 | -1.075 | 1.20E-02 | Down-regulated |
| 539 | hsa_circ_0009166 | -1.075 | 2.90E-02 | Down-regulated |
| 540 | hsa_circ_0059077 | -1.076 | 3.30E-02 | Down-regulated |
| 541 | hsa_circ_0012772 | -1.076 | 2.80E-02 | Down-regulated |
| 542 | hsa_circ_0007689 | -1.076 | 3.50E-02 | Down-regulated |
| 543 | hsa_circ_0032683 | -1.076 | 4.00E-02 | Down-regulated |
| 544 | hsa_circ_0086447 | -1.076 | 3.70E-02 | Down-regulated |
| 545 | hsa_circ_0050525 | -1.076 | 4.80E-02 | Down-regulated |
| 546 | hsa_circ_0032523 | -1.077 | 8.00E-03 | Down-regulated |
| 547 | hsa_circ_0003222 | -1.078 | 4.00E-02 | Down-regulated |
| 548 | hsa_circ_0002487 | -1.078 | 3.10E-02 | Down-regulated |
| 549 | hsa_circ_0032462 | -1.079 | 2.20E-02 | Down-regulated |
| 550 | hsa_circ_0005991 | -1.079 | 4.90E-02 | Down-regulated |
| 551 | hsa_circ_0005080 | -1.079 | 2.60E-02 | Down-regulated |
| 552 | hsa_circ_0009111 | -1.080 | 6.00E-03 | Down-regulated |
| 553 | hsa_circ_0004776 | -1.080 | 2.90E-02 | Down-regulated |
| 554 | hsa_circ_0038711 | -1.081 | 1.00E-02 | Down-regulated |
| 555 | hsa_circ_0004083 | -1.081 | 4.70E-02 | Down-regulated |
| 556 | hsa_circ_0072585 | -1.081 | 3.10E-02 | Down-regulated |
| 557 | hsa_circ_0008197 | -1.081 | 2.80E-02 | Down-regulated |
| 558 | hsa_circ_0072464 | -1.082 | 2.30E-02 | Down-regulated |
| 559 | hsa_circ_0031433 | -1.082 | 3.60E-02 | Down-regulated |
| 560 | hsa_circ_0002371 | -1.082 | 2.50E-02 | Down-regulated |
| 561 | hsa_circ_0005648 | -1.083 | 1.70E-02 | Down-regulated |
| 562 | hsa_circ_0009610 | -1.084 | 2.00E-02 | Down-regulated |
| 563 | hsa_circ_0018343 | -1.084 | 1.80E-02 | Down-regulated |
| 564 | hsa_circ_0003200 | -1.085 | 7.00E-03 | Down-regulated |
| 565 | hsa_circ_0000521 | -1.086 | 1.00E-02 | Down-regulated |
| 566 | hsa_circ_0006275 | -1.086 | 2.50E-02 | Down-regulated |
| 567 | hsa_circ_0036450 | -1.086 | 1.20E-02 | Down-regulated |
| 568 | hsa_circ_0054118 | -1.086 | 6.00E-03 | Down-regulated |
| 569 | hsa_circ_0016028 | -1.086 | 2.10E-02 | Down-regulated |
| 570 | hsa_circ_0061170 | -1.087 | 2.60E-02 | Down-regulated |
| 571 | hsa_circ_0007770 | -1.087 | 8.00E-03 | Down-regulated |
| 572 | hsa_circ_0000846 | -1.087 | 8.00E-03 | Down-regulated |
| 573 | hsa_circ_0006107 | -1.088 | 4.70E-02 | Down-regulated |
| 574 | hsa_circ_0004334 | -1.088 | 2.70E-02 | Down-regulated |
| 575 | hsa_circ_0007085 | -1.088 | 2.20E-02 | Down-regulated |
| 576 | hsa_circ_0063745 | -1.088 | 3.30E-02 | Down-regulated |
| 577 | hsa_circ_0007930 | -1.088 | 2.00E-02 | Down-regulated |
| 578 | hsa_circ_0064753 | -1.089 | 2.30E-02 | Down-regulated |
| 579 | hsa_circ_0001352 | -1.089 | 3.10E-02 | Down-regulated |
| 580 | hsa_circ_0006867 | -1.089 | 3.00E-03 | Down-regulated |
| 581 | hsa_circ_0021461 | -1.090 | 3.90E-02 | Down-regulated |
| 582 | hsa_circ_0076138 | -1.090 | 1.30E-02 | Down-regulated |
| 583 | hsa_circ_0003896 | -1.090 | 1.50E-02 | Down-regulated |
| 584 | hsa_circ_0075812 | -1.091 | 2.00E-02 | Down-regulated |
| 585 | hsa_circ_0072437 | -1.091 | 1.40E-02 | Down-regulated |
| 586 | hsa_circ_0007468 | -1.092 | 4.80E-02 | Down-regulated |
| 587 | hsa_circ_0005967 | -1.092 | 4.00E-02 | Down-regulated |
| 588 | hsa_circ_0029614 | -1.092 | 3.10E-02 | Down-regulated |
| 589 | hsa_circ_0070819 | -1.092 | 3.00E-03 | Down-regulated |
| 590 | hsa_circ_0045220 | -1.093 | 3.70E-02 | Down-regulated |
| 591 | hsa_circ_0006575 | -1.093 | 3.20E-02 | Down-regulated |
| 592 | hsa_circ_0017264 | -1.095 | 3.80E-02 | Down-regulated |
| 593 | hsa_circ_0075320 | -1.096 | 2.60E-02 | Down-regulated |
| 594 | hsa_circ_0012195 | -1.097 | 2.80E-02 | Down-regulated |
| 595 | hsa_circ_0008356 | -1.098 | 2.60E-02 | Down-regulated |
| 596 | hsa_circ_0077913 | -1.098 | 2.60E-02 | Down-regulated |
| 597 | hsa_circ_0005045 | -1.098 | 2.00E-02 | Down-regulated |
| 598 | hsa_circ_0052721 | -1.099 | 2.60E-02 | Down-regulated |
| 599 | hsa_circ_0007049 | -1.099 | 1.40E-02 | Down-regulated |
| 600 | hsa_circ_0050486 | -1.099 | 4.50E-02 | Down-regulated |
| 601 | hsa_circ_0006397 | -1.099 | 4.10E-02 | Down-regulated |
| 602 | hsa_circ_0062760 | -1.100 | 2.40E-02 | Down-regulated |
| 603 | hsa_circ_0084656 | -1.100 | 3.80E-02 | Down-regulated |
| 604 | hsa_circ_0049224 | -1.101 | 2.60E-02 | Down-regulated |
| 605 | hsa_circ_0000977 | -1.101 | 3.10E-02 | Down-regulated |
| 606 | hsa_circ_0007263 | -1.101 | 2.90E-02 | Down-regulated |
| 607 | hsa_circ_0053907 | -1.101 | 1.70E-02 | Down-regulated |
| 608 | hsa_circ_0055521 | -1.102 | 4.20E-02 | Down-regulated |
| 609 | hsa_circ_0047818 | -1.102 | 1.20E-02 | Down-regulated |
| 610 | hsa_circ_0030039 | -1.103 | 1.40E-02 | Down-regulated |
| 611 | hsa_circ_0044650 | -1.103 | 3.40E-02 | Down-regulated |
| 612 | hsa_circ_0027690 | -1.103 | 4.80E-02 | Down-regulated |
| 613 | hsa_circ_0069776 | -1.104 | 2.90E-02 | Down-regulated |
| 614 | hsa_circ_0038343 | -1.104 | 3.40E-02 | Down-regulated |
| 615 | hsa_circ_0024895 | -1.104 | 4.90E-02 | Down-regulated |
| 616 | hsa_circ_0003664 | -1.104 | 2.80E-02 | Down-regulated |
| 617 | hsa_circ_0032080 | -1.104 | 1.90E-02 | Down-regulated |
| 618 | hsa_circ_0002884 | -1.104 | 2.80E-02 | Down-regulated |
| 619 | hsa_circ_0085401 | -1.105 | 3.40E-02 | Down-regulated |
| 620 | hsa_circ_0075092 | -1.106 | 4.00E-02 | Down-regulated |
| 621 | hsa_circ_0002281 | -1.106 | 3.90E-02 | Down-regulated |
| 622 | hsa_circ_0035818 | -1.107 | 3.20E-02 | Down-regulated |
| 623 | hsa_circ_0086306 | -1.107 | 4.20E-02 | Down-regulated |
| 624 | hsa_circ_0049201 | -1.107 | 2.40E-02 | Down-regulated |
| 625 | hsa_circ_0025850 | -1.108 | 2.90E-02 | Down-regulated |
| 626 | hsa_circ_0060020 | -1.109 | 1.90E-02 | Down-regulated |
| 627 | hsa_circ_0089474 | -1.110 | 1.50E-02 | Down-regulated |
| 628 | hsa_circ_0064501 | -1.110 | 3.30E-02 | Down-regulated |
| 629 | hsa_circ_0064996 | -1.110 | 3.80E-02 | Down-regulated |
| 630 | hsa_circ_0031085 | -1.111 | 1.60E-02 | Down-regulated |
| 631 | hsa_circ_0072592 | -1.112 | 4.60E-02 | Down-regulated |
| 632 | hsa_circ_0005106 | -1.112 | 1.80E-02 | Down-regulated |
| 633 | hsa_circ_0083849 | -1.112 | 2.60E-02 | Down-regulated |
| 634 | hsa_circ_0038479 | -1.112 | 7.00E-03 | Down-regulated |
| 635 | hsa_circ_0003148 | -1.112 | 2.60E-02 | Down-regulated |
| 636 | hsa_circ_0016868 | -1.113 | 1.10E-02 | Down-regulated |
| 637 | hsa_circ_0090039 | -1.113 | 4.60E-02 | Down-regulated |
| 638 | hsa_circ_0056550 | -1.113 | 2.30E-02 | Down-regulated |
| 639 | hsa_circ_0046265 | -1.114 | 2.10E-02 | Down-regulated |
| 640 | hsa_circ_0074816 | -1.114 | 5.00E-02 | Down-regulated |
| 641 | hsa_circ_0083395 | -1.114 | 4.40E-02 | Down-regulated |
| 642 | hsa_circ_0040747 | -1.115 | 2.00E-03 | Down-regulated |
| 643 | hsa_circ_0067108 | -1.116 | 1.80E-02 | Down-regulated |
| 644 | hsa_circ_0000234 | -1.116 | 4.90E-02 | Down-regulated |
| 645 | hsa_circ_0040540 | -1.116 | 1.20E-02 | Down-regulated |
| 646 | hsa_circ_0001913 | -1.116 | 1.80E-02 | Down-regulated |
| 647 | hsa_circ_0005964 | -1.116 | 2.70E-02 | Down-regulated |
| 648 | hsa_circ_0047347 | -1.117 | 4.10E-02 | Down-regulated |
| 649 | hsa_circ_0032971 | -1.117 | 6.00E-03 | Down-regulated |
| 650 | hsa_circ_0011162 | -1.117 | 1.20E-02 | Down-regulated |
| 651 | hsa_circ_0050894 | -1.118 | 1.30E-02 | Down-regulated |
| 652 | hsa_circ_0016586 | -1.118 | 2.80E-02 | Down-regulated |
| 653 | hsa_circ_0005739 | -1.118 | 4.00E-02 | Down-regulated |
| 654 | hsa_circ_0084606 | -1.120 | 1.10E-02 | Down-regulated |
| 655 | hsa_circ_0002447 | -1.120 | 4.80E-02 | Down-regulated |
| 656 | hsa_circ_0041872 | -1.121 | 4.50E-02 | Down-regulated |
| 657 | hsa_circ_0087051 | -1.121 | 1.70E-02 | Down-regulated |
| 658 | hsa_circ_0077142 | -1.121 | 3.50E-02 | Down-regulated |
| 659 | hsa_circ_0076439 | -1.122 | 3.30E-02 | Down-regulated |
| 660 | hsa_circ_0026978 | -1.122 | 2.50E-02 | Down-regulated |
| 661 | hsa_circ_0002444 | -1.122 | 3.70E-02 | Down-regulated |
| 662 | hsa_circ_0002781 | -1.122 | 1.60E-02 | Down-regulated |
| 663 | hsa_circ_0008184 | -1.122 | 3.70E-02 | Down-regulated |
| 664 | hsa_circ_0002617 | -1.123 | 1.60E-02 | Down-regulated |
| 665 | hsa_circ_0005046 | -1.123 | 3.00E-02 | Down-regulated |
| 666 | hsa_circ_0023865 | -1.123 | 1.10E-02 | Down-regulated |
| 667 | hsa_circ_0001591 | -1.123 | 7.00E-03 | Down-regulated |
| 668 | hsa_circ_0006037 | -1.123 | 4.80E-02 | Down-regulated |
| 669 | hsa_circ_0031655 | -1.123 | 3.30E-02 | Down-regulated |
| 670 | hsa_circ_0061985 | -1.123 | 4.50E-02 | Down-regulated |
| 671 | hsa_circ_0025859 | -1.124 | 1.30E-02 | Down-regulated |
| 672 | hsa_circ_0082021 | -1.124 | 3.10E-02 | Down-regulated |
| 673 | hsa_circ_0039365 | -1.124 | 1.90E-02 | Down-regulated |
| 674 | hsa_circ_0001738 | -1.124 | 3.30E-02 | Down-regulated |
| 675 | hsa_circ_0016601 | -1.125 | 1.10E-02 | Down-regulated |
| 676 | hsa_circ_0004631 | -1.125 | 1.70E-02 | Down-regulated |
| 677 | hsa_circ_0083299 | -1.125 | 3.20E-02 | Down-regulated |
| 678 | hsa_circ_0001025 | -1.126 | 3.10E-02 | Down-regulated |
| 679 | hsa_circ_0036359 | -1.126 | 3.60E-02 | Down-regulated |
| 680 | hsa_circ_0058851 | -1.126 | 4.30E-02 | Down-regulated |
| 681 | hsa_circ_0005248 | -1.127 | 2.40E-02 | Down-regulated |
| 682 | hsa_circ_0007816 | -1.128 | 3.80E-02 | Down-regulated |
| 683 | hsa_circ_0087581 | -1.128 | 4.90E-02 | Down-regulated |
| 684 | hsa_circ_0071418 | -1.128 | 3.50E-02 | Down-regulated |
| 685 | hsa_circ_0055066 | -1.130 | 4.50E-02 | Down-regulated |
| 686 | hsa_circ_0001345 | -1.131 | 3.00E-02 | Down-regulated |
| 687 | hsa_circ_0002820 | -1.131 | 3.10E-02 | Down-regulated |
| 688 | hsa_circ_0004513 | -1.131 | 6.00E-03 | Down-regulated |
| 689 | hsa_circ_0000413 | -1.131 | 4.10E-02 | Down-regulated |
| 690 | hsa_circ_0014088 | -1.131 | 5.00E-03 | Down-regulated |
| 691 | hsa_circ_0003127 | -1.131 | 3.90E-02 | Down-regulated |
| 692 | hsa_circ_0063329 | -1.132 | 4.20E-02 | Down-regulated |
| 693 | hsa_circ_0077861 | -1.132 | 2.80E-02 | Down-regulated |
| 694 | hsa_circ_0006818 | -1.132 | 2.20E-02 | Down-regulated |
| 695 | hsa_circ_0078224 | -1.132 | 2.90E-02 | Down-regulated |
| 696 | hsa_circ_0087283 | -1.133 | 2.00E-02 | Down-regulated |
| 697 | hsa_circ_0070356 | -1.133 | 2.80E-02 | Down-regulated |
| 698 | hsa_circ_0006158 | -1.133 | 2.30E-02 | Down-regulated |
| 699 | hsa_circ_0002998 | -1.134 | 4.80E-02 | Down-regulated |
| 700 | hsa_circ_0002887 | -1.134 | 4.90E-02 | Down-regulated |
| 701 | hsa_circ_0001431 | -1.135 | 4.60E-02 | Down-regulated |
| 702 | hsa_circ_0018550 | -1.135 | 4.00E-02 | Down-regulated |
| 703 | hsa_circ_0006605 | -1.135 | 2.70E-02 | Down-regulated |
| 704 | hsa_circ_0018814 | -1.135 | 4.50E-02 | Down-regulated |
| 705 | hsa_circ_0081730 | -1.136 | 1.10E-02 | Down-regulated |
| 706 | hsa_circ_0001011 | -1.136 | 3.60E-02 | Down-regulated |
| 707 | hsa_circ_0012905 | -1.136 | 4.00E-03 | Down-regulated |
| 708 | hsa_circ_0005362 | -1.137 | 4.90E-02 | Down-regulated |
| 709 | hsa_circ_0090302 | -1.137 | 9.00E-03 | Down-regulated |
| 710 | hsa_circ_0068293 | -1.138 | 1.70E-02 | Down-regulated |
| 711 | hsa_circ_0003223 | -1.138 | 2.60E-02 | Down-regulated |
| 712 | hsa_circ_0002707 | -1.139 | 3.20E-02 | Down-regulated |
| 713 | hsa_circ_0063862 | -1.139 | 2.20E-02 | Down-regulated |
| 714 | hsa_circ_0025636 | -1.139 | 1.50E-02 | Down-regulated |
| 715 | hsa_circ_0075445 | -1.139 | 3.40E-02 | Down-regulated |
| 716 | hsa_circ_0072060 | -1.140 | 3.40E-02 | Down-regulated |
| 717 | hsa_circ_0061613 | -1.140 | 2.30E-02 | Down-regulated |
| 718 | hsa_circ_0029628 | -1.140 | 1.30E-02 | Down-regulated |
| 719 | hsa_circ_0013533 | -1.141 | 2.60E-02 | Down-regulated |
| 720 | hsa_circ_0007676 | -1.141 | 2.20E-02 | Down-regulated |
| 721 | hsa_circ_0003875 | -1.141 | 3.50E-02 | Down-regulated |
| 722 | hsa_circ_0000619 | -1.142 | 1.70E-02 | Down-regulated |
| 723 | hsa_circ_0052941 | -1.142 | 3.00E-02 | Down-regulated |
| 724 | hsa_circ_0016600 | -1.143 | 3.50E-02 | Down-regulated |
| 725 | hsa_circ_0030175 | -1.143 | 8.00E-03 | Down-regulated |
| 726 | hsa_circ_0003126 | -1.143 | 4.50E-02 | Down-regulated |
| 727 | hsa_circ_0078353 | -1.143 | 4.10E-02 | Down-regulated |
| 728 | hsa_circ_0077225 | -1.143 | 1.30E-02 | Down-regulated |
| 729 | hsa_circ_0004680 | -1.143 | 2.00E-02 | Down-regulated |
| 730 | hsa_circ_0065908 | -1.144 | 3.90E-02 | Down-regulated |
| 731 | hsa_circ_0028669 | -1.144 | 4.60E-02 | Down-regulated |
| 732 | hsa_circ_0045534 | -1.145 | 4.10E-02 | Down-regulated |
| 733 | hsa_circ_0002041 | -1.145 | 2.90E-02 | Down-regulated |
| 734 | hsa_circ_0000196 | -1.145 | 2.10E-02 | Down-regulated |
| 735 | hsa_circ_0016263 | -1.145 | 3.50E-02 | Down-regulated |
| 736 | hsa_circ_0073593 | -1.146 | 3.40E-02 | Down-regulated |
| 737 | hsa_circ_0004700 | -1.146 | 2.20E-02 | Down-regulated |
| 738 | hsa_circ_0038673 | -1.146 | 3.90E-02 | Down-regulated |
| 739 | hsa_circ_0086627 | -1.146 | 1.70E-02 | Down-regulated |
| 740 | hsa_circ_0023908 | -1.146 | 3.10E-02 | Down-regulated |
| 741 | hsa_circ_0002854 | -1.147 | 4.90E-02 | Down-regulated |
| 742 | hsa_circ_0069376 | -1.147 | 2.90E-02 | Down-regulated |
| 743 | hsa_circ_0002687 | -1.147 | 1.40E-02 | Down-regulated |
| 744 | hsa_circ_0000604 | -1.147 | 2.70E-02 | Down-regulated |
| 745 | hsa_circ_0000154 | -1.148 | 1.50E-02 | Down-regulated |
| 746 | hsa_circ_0009434 | -1.148 | 3.60E-02 | Down-regulated |
| 747 | hsa_circ_0045989 | -1.149 | 1.70E-02 | Down-regulated |
| 748 | hsa_circ_0008138 | -1.149 | 1.70E-02 | Down-regulated |
| 749 | hsa_circ_0005062 | -1.149 | 4.10E-02 | Down-regulated |
| 750 | hsa_circ_0000698 | -1.149 | 2.00E-02 | Down-regulated |
| 751 | hsa_circ_0061111 | -1.149 | 1.00E-02 | Down-regulated |
| 752 | hsa_circ_0028342 | -1.150 | 4.50E-02 | Down-regulated |
| 753 | hsa_circ_0079812 | -1.150 | 2.00E-02 | Down-regulated |
| 754 | hsa_circ_0003976 | -1.151 | 2.80E-02 | Down-regulated |
| 755 | hsa_circ_0003000 | -1.151 | 1.80E-02 | Down-regulated |
| 756 | hsa_circ_0008821 | -1.151 | 2.10E-02 | Down-regulated |
| 757 | hsa_circ_0077173 | -1.152 | 1.20E-02 | Down-regulated |
| 758 | hsa_circ_0000106 | -1.152 | 2.20E-02 | Down-regulated |
| 759 | hsa_circ_0018497 | -1.153 | 2.00E-02 | Down-regulated |
| 760 | hsa_circ_0073659 | -1.153 | 2.60E-02 | Down-regulated |
| 761 | hsa_circ_0063408 | -1.153 | 3.00E-02 | Down-regulated |
| 762 | hsa_circ_0001359 | -1.153 | 3.00E-02 | Down-regulated |
| 763 | hsa_circ_0069451 | -1.153 | 4.90E-02 | Down-regulated |
| 764 | hsa_circ_0018902 | -1.154 | 5.00E-02 | Down-regulated |
| 765 | hsa_circ_0072917 | -1.154 | 4.40E-02 | Down-regulated |
| 766 | hsa_circ_0006532 | -1.154 | 1.10E-02 | Down-regulated |
| 767 | hsa_circ_0008666 | -1.154 | 2.50E-02 | Down-regulated |
| 768 | hsa_circ_0008882 | -1.154 | 1.00E-02 | Down-regulated |
| 769 | hsa_circ_0068281 | -1.155 | 1.80E-02 | Down-regulated |
| 770 | hsa_circ_0081004 | -1.156 | 1.60E-02 | Down-regulated |
| 771 | hsa_circ_0085050 | -1.156 | 3.50E-02 | Down-regulated |
| 772 | hsa_circ_0067103 | -1.156 | 3.20E-02 | Down-regulated |
| 773 | hsa_circ_0054809 | -1.157 | 4.20E-02 | Down-regulated |
| 774 | hsa_circ_0007370 | -1.158 | 3.30E-02 | Down-regulated |
| 775 | hsa_circ_0068014 | -1.158 | 3.00E-02 | Down-regulated |
| 776 | hsa_circ_0005655 | -1.158 | 1.20E-02 | Down-regulated |
| 777 | hsa_circ_0000785 | -1.158 | 2.00E-02 | Down-regulated |
| 778 | hsa_circ_0085465 | -1.159 | 3.20E-02 | Down-regulated |
| 779 | hsa_circ_0000328 | -1.159 | 2.00E-02 | Down-regulated |
| 780 | hsa_circ_0006693 | -1.159 | 4.60E-02 | Down-regulated |
| 781 | hsa_circ_0071439 | -1.159 | 4.00E-03 | Down-regulated |
| 782 | hsa_circ_0004441 | -1.160 | 1.10E-02 | Down-regulated |
| 783 | hsa_circ_0084885 | -1.161 | 3.20E-02 | Down-regulated |
| 784 | hsa_circ_0031147 | -1.161 | 3.40E-02 | Down-regulated |
| 785 | hsa_circ_0073653 | -1.161 | 4.80E-02 | Down-regulated |
| 786 | hsa_circ_0005907 | -1.161 | 3.70E-02 | Down-regulated |
| 787 | hsa_circ_0003033 | -1.162 | 3.20E-02 | Down-regulated |
| 788 | hsa_circ_0026652 | -1.162 | 2.10E-02 | Down-regulated |
| 789 | hsa_circ_0083795 | -1.163 | 1.90E-02 | Down-regulated |
| 790 | hsa_circ_0057786 | -1.163 | 2.00E-02 | Down-regulated |
| 791 | hsa_circ_0029813 | -1.163 | 1.60E-02 | Down-regulated |
| 792 | hsa_circ_0011290 | -1.163 | 3.70E-02 | Down-regulated |
| 793 | hsa_circ_0001017 | -1.164 | 2.90E-02 | Down-regulated |
| 794 | hsa_circ_0081227 | -1.164 | 2.40E-02 | Down-regulated |
| 795 | hsa_circ_0061975 | -1.165 | 1.00E-02 | Down-regulated |
| 796 | hsa_circ_0008847 | -1.165 | 4.70E-02 | Down-regulated |
| 797 | hsa_circ_0001632 | -1.165 | 4.20E-02 | Down-regulated |
| 798 | hsa_circ_0002633 | -1.166 | 2.30E-02 | Down-regulated |
| 799 | hsa_circ_0031632 | -1.166 | 1.80E-02 | Down-regulated |
| 800 | hsa_circ_0075270 | -1.167 | 2.20E-02 | Down-regulated |
| 801 | hsa_circ_0082148 | -1.167 | 1.70E-02 | Down-regulated |
| 802 | hsa_circ_0016369 | -1.167 | 3.80E-02 | Down-regulated |
| 803 | hsa_circ_0001194 | -1.168 | 2.60E-02 | Down-regulated |
| 804 | hsa_circ_0057074 | -1.169 | 2.60E-02 | Down-regulated |
| 805 | hsa_circ_0028215 | -1.169 | 1.50E-02 | Down-regulated |
| 806 | hsa_circ_0084716 | -1.169 | 1.50E-02 | Down-regulated |
| 807 | hsa_circ_0083530 | -1.169 | 4.40E-02 | Down-regulated |
| 808 | hsa_circ_0080917 | -1.170 | 2.30E-02 | Down-regulated |
| 809 | hsa_circ_0001871 | -1.170 | 1.50E-02 | Down-regulated |
| 810 | hsa_circ_0009924 | -1.170 | 2.30E-02 | Down-regulated |
| 811 | hsa_circ_0088498 | -1.170 | 3.60E-02 | Down-regulated |
| 812 | hsa_circ_0054124 | -1.171 | 9.00E-03 | Down-regulated |
| 813 | hsa_circ_0014717 | -1.171 | 1.70E-02 | Down-regulated |
| 814 | hsa_circ_0003661 | -1.171 | 2.10E-02 | Down-regulated |
| 815 | hsa_circ_0052852 | -1.172 | 3.50E-02 | Down-regulated |
| 816 | hsa_circ_0006565 | -1.172 | 9.00E-03 | Down-regulated |
| 817 | hsa_circ_0008751 | -1.173 | 1.00E-02 | Down-regulated |
| 818 | hsa_circ_0088568 | -1.173 | 3.50E-02 | Down-regulated |
| 819 | hsa_circ_0026153 | -1.173 | 2.70E-02 | Down-regulated |
| 820 | hsa_circ_0012824 | -1.174 | 5.00E-02 | Down-regulated |
| 821 | hsa_circ_0064110 | -1.174 | 2.80E-02 | Down-regulated |
| 822 | hsa_circ_0071538 | -1.174 | 3.50E-02 | Down-regulated |
| 823 | hsa_circ_0082501 | -1.176 | 1.70E-02 | Down-regulated |
| 824 | hsa_circ_0008030 | -1.178 | 2.20E-02 | Down-regulated |
| 825 | hsa_circ_0001501 | -1.178 | 2.10E-02 | Down-regulated |
| 826 | hsa_circ_0034343 | -1.179 | 4.90E-02 | Down-regulated |
| 827 | hsa_circ_0001417 | -1.179 | 4.80E-02 | Down-regulated |
| 828 | hsa_circ_0000641 | -1.180 | 6.00E-03 | Down-regulated |
| 829 | hsa_circ_0003697 | -1.180 | 3.20E-02 | Down-regulated |
| 830 | hsa_circ_0089282 | -1.180 | 3.80E-02 | Down-regulated |
| 831 | hsa_circ_0055940 | -1.181 | 1.00E-02 | Down-regulated |
| 832 | hsa_circ_0016627 | -1.181 | 4.00E-03 | Down-regulated |
| 833 | hsa_circ_0054949 | -1.181 | 2.30E-02 | Down-regulated |
| 834 | hsa_circ_0036083 | -1.181 | 2.30E-02 | Down-regulated |
| 835 | hsa_circ_0005365 | -1.181 | 2.70E-02 | Down-regulated |
| 836 | hsa_circ_0059501 | -1.182 | 3.70E-02 | Down-regulated |
| 837 | hsa_circ_0006008 | -1.182 | 1.40E-02 | Down-regulated |
| 838 | hsa_circ_0034528 | -1.182 | 5.00E-03 | Down-regulated |
| 839 | hsa_circ_0064516 | -1.183 | 2.70E-02 | Down-regulated |
| 840 | hsa_circ_0062191 | -1.183 | 5.00E-03 | Down-regulated |
| 841 | hsa_circ_0006629 | -1.183 | 1.70E-02 | Down-regulated |
| 842 | hsa_circ_0054134 | -1.183 | 4.00E-02 | Down-regulated |
| 843 | hsa_circ_0017777 | -1.184 | 1.80E-02 | Down-regulated |
| 844 | hsa_circ_0068264 | -1.184 | 1.20E-02 | Down-regulated |
| 845 | hsa_circ_0078784 | -1.185 | 1.00E-02 | Down-regulated |
| 846 | hsa_circ_0009360 | -1.185 | 2.40E-02 | Down-regulated |
| 847 | hsa_circ_0027093 | -1.185 | 3.10E-02 | Down-regulated |
| 848 | hsa_circ_0058040 | -1.185 | 3.70E-02 | Down-regulated |
| 849 | hsa_circ_0074556 | -1.185 | 1.10E-02 | Down-regulated |
| 850 | hsa_circ_0000126 | -1.185 | 1.50E-02 | Down-regulated |
| 851 | hsa_circ_0007232 | -1.185 | 2.30E-02 | Down-regulated |
| 852 | hsa_circ_0090040 | -1.186 | 2.30E-02 | Down-regulated |
| 853 | hsa_circ_0026046 | -1.186 | 4.10E-02 | Down-regulated |
| 854 | hsa_circ_0069117 | -1.186 | 1.10E-02 | Down-regulated |
| 855 | hsa_circ_0005990 | -1.186 | 4.80E-02 | Down-regulated |
| 856 | hsa_circ_0091482 | -1.187 | 1.80E-02 | Down-regulated |
| 857 | hsa_circ_0008594 | -1.187 | 3.90E-02 | Down-regulated |
| 858 | hsa_circ_0063763 | -1.187 | 3.70E-02 | Down-regulated |
| 859 | hsa_circ_0002452 | -1.187 | 4.30E-02 | Down-regulated |
| 860 | hsa_circ_0029853 | -1.187 | 4.00E-02 | Down-regulated |
| 861 | hsa_circ_0055947 | -1.188 | 2.70E-02 | Down-regulated |
| 862 | hsa_circ_0071041 | -1.188 | 2.80E-02 | Down-regulated |
| 863 | hsa_circ_0062165 | -1.188 | 2.30E-02 | Down-regulated |
| 864 | hsa_circ_0017997 | -1.189 | 3.80E-02 | Down-regulated |
| 865 | hsa_circ_0017693 | -1.189 | 1.00E-03 | Down-regulated |
| 866 | hsa_circ_0006542 | -1.189 | 3.20E-02 | Down-regulated |
| 867 | hsa_circ_0002995 | -1.189 | 9.00E-03 | Down-regulated |
| 868 | hsa_circ_0008752 | -1.189 | 1.00E-02 | Down-regulated |
| 869 | hsa_circ_0008244 | -1.189 | 4.10E-02 | Down-regulated |
| 870 | hsa_circ_0053441 | -1.189 | 4.10E-02 | Down-regulated |
| 871 | hsa_circ_0029069 | -1.190 | 3.50E-02 | Down-regulated |
| 872 | hsa_circ_0077382 | -1.190 | 2.00E-02 | Down-regulated |
| 873 | hsa_circ_0007658 | -1.190 | 7.00E-03 | Down-regulated |
| 874 | hsa_circ_0001686 | -1.190 | 1.60E-02 | Down-regulated |
| 875 | hsa_circ_0072380 | -1.190 | 1.20E-02 | Down-regulated |
| 876 | hsa_circ_0074243 | -1.191 | 3.20E-02 | Down-regulated |
| 877 | hsa_circ_0036374 | -1.192 | 3.00E-02 | Down-regulated |
| 878 | hsa_circ_0007656 | -1.192 | 3.20E-02 | Down-regulated |
| 879 | hsa_circ_0069891 | -1.193 | 1.80E-02 | Down-regulated |
| 880 | hsa_circ_0073616 | -1.194 | 1.50E-02 | Down-regulated |
| 881 | hsa_circ_0026286 | -1.194 | 1.60E-02 | Down-regulated |
| 882 | hsa_circ_0023928 | -1.194 | 1.80E-02 | Down-regulated |
| 883 | hsa_circ_0035217 | -1.195 | 1.40E-02 | Down-regulated |
| 884 | hsa_circ_0001134 | -1.195 | 2.80E-02 | Down-regulated |
| 885 | hsa_circ_0036398 | -1.195 | 2.40E-02 | Down-regulated |
| 886 | hsa_circ_0070003 | -1.195 | 1.10E-02 | Down-regulated |
| 887 | hsa_circ_0007100 | -1.195 | 4.50E-02 | Down-regulated |
| 888 | hsa_circ_0031027 | -1.196 | 2.00E-02 | Down-regulated |
| 889 | hsa_circ_0081001 | -1.196 | 4.10E-02 | Down-regulated |
| 890 | hsa_circ_0050461 | -1.196 | 2.40E-02 | Down-regulated |
| 891 | hsa_circ_0006497 | -1.197 | 4.20E-02 | Down-regulated |
| 892 | hsa_circ_0072547 | -1.197 | 2.10E-02 | Down-regulated |
| 893 | hsa_circ_0028883 | -1.197 | 2.40E-02 | Down-regulated |
| 894 | hsa_circ_0005759 | -1.197 | 3.10E-02 | Down-regulated |
| 895 | hsa_circ_0084148 | -1.198 | 4.80E-02 | Down-regulated |
| 896 | hsa_circ_0000943 | -1.198 | 2.50E-02 | Down-regulated |
| 897 | hsa_circ_0008495 | -1.198 | 2.40E-02 | Down-regulated |
| 898 | hsa_circ_0009002 | -1.198 | 3.20E-02 | Down-regulated |
| 899 | hsa_circ_0013235 | -1.198 | 1.90E-02 | Down-regulated |
| 900 | hsa_circ_0071991 | -1.198 | 2.50E-02 | Down-regulated |
| 901 | hsa_circ_0002853 | -1.199 | 2.30E-02 | Down-regulated |
| 902 | hsa_circ_0057874 | -1.199 | 4.70E-02 | Down-regulated |
| 903 | hsa_circ_0003071 | -1.199 | 3.00E-02 | Down-regulated |
| 904 | hsa_circ_0070930 | -1.200 | 3.10E-02 | Down-regulated |
| 905 | hsa_circ_0082352 | -1.200 | 4.00E-02 | Down-regulated |
| 906 | hsa_circ_0028048 | -1.201 | 2.50E-02 | Down-regulated |
| 907 | hsa_circ_0059577 | -1.201 | 7.00E-03 | Down-regulated |
| 908 | hsa_circ_0049962 | -1.201 | 2.10E-02 | Down-regulated |
| 909 | hsa_circ_0007923 | -1.201 | 9.00E-03 | Down-regulated |
| 910 | hsa_circ_0020151 | -1.202 | 1.80E-02 | Down-regulated |
| 911 | hsa_circ_0034095 | -1.202 | 3.80E-02 | Down-regulated |
| 912 | hsa_circ_0090999 | -1.202 | 1.50E-02 | Down-regulated |
| 913 | hsa_circ_0000793 | -1.202 | 3.50E-02 | Down-regulated |
| 914 | hsa_circ_0005702 | -1.203 | 2.40E-02 | Down-regulated |
| 915 | hsa_circ_0008261 | -1.203 | 4.70E-02 | Down-regulated |
| 916 | hsa_circ_0014315 | -1.203 | 3.50E-02 | Down-regulated |
| 917 | hsa_circ_0082381 | -1.203 | 9.00E-03 | Down-regulated |
| 918 | hsa_circ_0079518 | -1.204 | 1.70E-02 | Down-regulated |
| 919 | hsa_circ_0087006 | -1.204 | 4.10E-02 | Down-regulated |
| 920 | hsa_circ_0013744 | -1.204 | 1.70E-02 | Down-regulated |
| 921 | hsa_circ_0082734 | -1.204 | 3.60E-02 | Down-regulated |
| 922 | hsa_circ_0002844 | -1.204 | 1.80E-02 | Down-regulated |
| 923 | hsa_circ_0001989 | -1.205 | 2.10E-02 | Down-regulated |
| 924 | hsa_circ_0078373 | -1.206 | 2.10E-02 | Down-regulated |
| 925 | hsa_circ_0060953 | -1.206 | 1.60E-02 | Down-regulated |
| 926 | hsa_circ_0026457 | -1.207 | 3.10E-02 | Down-regulated |
| 927 | hsa_circ_0006583 | -1.207 | 1.20E-02 | Down-regulated |
| 928 | hsa_circ_0003795 | -1.208 | 1.70E-02 | Down-regulated |
| 929 | hsa_circ_0009161 | -1.208 | 3.80E-02 | Down-regulated |
| 930 | hsa_circ_0045590 | -1.208 | 2.80E-02 | Down-regulated |
| 931 | hsa_circ_0057183 | -1.208 | 1.10E-02 | Down-regulated |
| 932 | hsa_circ_0007745 | -1.209 | 3.40E-02 | Down-regulated |
| 933 | hsa_circ_0021591 | -1.209 | 1.50E-02 | Down-regulated |
| 934 | hsa_circ_0044966 | -1.209 | 2.10E-02 | Down-regulated |
| 935 | hsa_circ_0074712 | -1.210 | 4.40E-02 | Down-regulated |
| 936 | hsa_circ_0082415 | -1.210 | 2.80E-02 | Down-regulated |
| 937 | hsa_circ_0063526 | -1.210 | 2.80E-02 | Down-regulated |
| 938 | hsa_circ_0065301 | -1.210 | 4.50E-02 | Down-regulated |
| 939 | hsa_circ_0006485 | -1.211 | 1.10E-02 | Down-regulated |
| 940 | hsa_circ_0068601 | -1.211 | 2.50E-02 | Down-regulated |
| 941 | hsa_circ_0057648 | -1.211 | 4.90E-02 | Down-regulated |
| 942 | hsa_circ_0007407 | -1.212 | 7.00E-03 | Down-regulated |
| 943 | hsa_circ_0053535 | -1.213 | 1.80E-02 | Down-regulated |
| 944 | hsa_circ_0077179 | -1.213 | 2.00E-02 | Down-regulated |
| 945 | hsa_circ_0035406 | -1.213 | 2.30E-02 | Down-regulated |
| 946 | hsa_circ_0044370 | -1.214 | 5.00E-02 | Down-regulated |
| 947 | hsa_circ_0004977 | -1.215 | 3.30E-02 | Down-regulated |
| 948 | hsa_circ_0008055 | -1.215 | 3.30E-02 | Down-regulated |
| 949 | hsa_circ_0001399 | -1.216 | 2.70E-02 | Down-regulated |
| 950 | hsa_circ_0043293 | -1.216 | 2.20E-02 | Down-regulated |
| 951 | hsa_circ_0076248 | -1.216 | 2.20E-02 | Down-regulated |
| 952 | hsa_circ_0004079 | -1.217 | 4.80E-02 | Down-regulated |
| 953 | hsa_circ_0006987 | -1.217 | 3.50E-02 | Down-regulated |
| 954 | hsa_circ_0002180 | -1.218 | 1.00E-02 | Down-regulated |
| 955 | hsa_circ_0080831 | -1.218 | 1.20E-02 | Down-regulated |
| 956 | hsa_circ_0008432 | -1.218 | 4.90E-02 | Down-regulated |
| 957 | hsa_circ_0014628 | -1.218 | 3.20E-02 | Down-regulated |
| 958 | hsa_circ_0025548 | -1.219 | 3.40E-02 | Down-regulated |
| 959 | hsa_circ_0072272 | -1.219 | 1.40E-02 | Down-regulated |
| 960 | hsa_circ_0008501 | -1.222 | 1.30E-02 | Down-regulated |
| 961 | hsa_circ_0003673 | -1.224 | 2.40E-02 | Down-regulated |
| 962 | hsa_circ_0057727 | -1.224 | 2.20E-02 | Down-regulated |
| 963 | hsa_circ_0001420 | -1.224 | 3.50E-02 | Down-regulated |
| 964 | hsa_circ_0002829 | -1.224 | 1.60E-02 | Down-regulated |
| 965 | hsa_circ_0006535 | -1.224 | 1.80E-02 | Down-regulated |
| 966 | hsa_circ_0000842 | -1.225 | 2.90E-02 | Down-regulated |
| 967 | hsa_circ_0054345 | -1.225 | 1.40E-02 | Down-regulated |
| 968 | hsa_circ_0005191 | -1.226 | 2.30E-02 | Down-regulated |
| 969 | hsa_circ_0014601 | -1.227 | 3.80E-02 | Down-regulated |
| 970 | hsa_circ_0060122 | -1.227 | 2.20E-02 | Down-regulated |
| 971 | hsa_circ_0043033 | -1.227 | 4.90E-02 | Down-regulated |
| 972 | hsa_circ_0007042 | -1.227 | 1.50E-02 | Down-regulated |
| 973 | hsa_circ_0077417 | -1.227 | 2.70E-02 | Down-regulated |
| 974 | hsa_circ_0010015 | -1.228 | 3.20E-02 | Down-regulated |
| 975 | hsa_circ_0086492 | -1.228 | 1.40E-02 | Down-regulated |
| 976 | hsa_circ_0037809 | -1.228 | 1.30E-02 | Down-regulated |
| 977 | hsa_circ_0092163 | -1.230 | 2.90E-02 | Down-regulated |
| 978 | hsa_circ_0073819 | -1.230 | 1.40E-02 | Down-regulated |
| 979 | hsa_circ_0000603 | -1.231 | 1.80E-02 | Down-regulated |
| 980 | hsa_circ_0017943 | -1.231 | 3.60E-02 | Down-regulated |
| 981 | hsa_circ_0035505 | -1.232 | 1.30E-02 | Down-regulated |
| 982 | hsa_circ_0082139 | -1.232 | 3.90E-02 | Down-regulated |
| 983 | hsa_circ_0000599 | -1.232 | 1.60E-02 | Down-regulated |
| 984 | hsa_circ_0043423 | -1.232 | 3.30E-02 | Down-regulated |
| 985 | hsa_circ_0031897 | -1.232 | 2.10E-02 | Down-regulated |
| 986 | hsa_circ_0004956 | -1.232 | 3.50E-02 | Down-regulated |
| 987 | hsa_circ_0002537 | -1.233 | 1.80E-02 | Down-regulated |
| 988 | hsa_circ_0027513 | -1.233 | 8.00E-03 | Down-regulated |
| 989 | hsa_circ_0058565 | -1.234 | 2.90E-02 | Down-regulated |
| 990 | hsa_circ_0088515 | -1.234 | 4.10E-02 | Down-regulated |
| 991 | hsa_circ_0031089 | -1.235 | 2.90E-02 | Down-regulated |
| 992 | hsa_circ_0080252 | -1.235 | 3.00E-03 | Down-regulated |
| 993 | hsa_circ_0019390 | -1.236 | 3.20E-02 | Down-regulated |
| 994 | hsa_circ_0001181 | -1.236 | 2.60E-02 | Down-regulated |
| 995 | hsa_circ_0079933 | -1.237 | 4.30E-02 | Down-regulated |
| 996 | hsa_circ_0070401 | -1.238 | 4.00E-02 | Down-regulated |
| 997 | hsa_circ_0082149 | -1.238 | 2.20E-02 | Down-regulated |
| 998 | hsa_circ_0091994 | -1.238 | 4.20E-02 | Down-regulated |
| 999 | hsa_circ_0022308 | -1.238 | 2.60E-02 | Down-regulated |
| 1000 | hsa_circ_0025614 | -1.238 | 1.20E-02 | Down-regulated |
| 1001 | hsa_circ_0044365 | -1.239 | 3.20E-02 | Down-regulated |
| 1002 | hsa_circ_0083388 | -1.239 | 1.00E-02 | Down-regulated |
| 1003 | hsa_circ_0004133 | -1.239 | 3.10E-02 | Down-regulated |
| 1004 | hsa_circ_0001040 | -1.239 | 1.40E-02 | Down-regulated |
| 1005 | hsa_circ_0009114 | -1.240 | 2.70E-02 | Down-regulated |
| 1006 | hsa_circ_0001028 | -1.241 | 2.70E-02 | Down-regulated |
| 1007 | hsa_circ_0012610 | -1.242 | 9.00E-03 | Down-regulated |
| 1008 | hsa_circ_0034920 | -1.242 | 7.00E-03 | Down-regulated |
| 1009 | hsa_circ_0025899 | -1.242 | 1.10E-02 | Down-regulated |
| 1010 | hsa_circ_0034701 | -1.242 | 1.40E-02 | Down-regulated |
| 1011 | hsa_circ_0079508 | -1.244 | 2.20E-02 | Down-regulated |
| 1012 | hsa_circ_0003694 | -1.244 | 3.60E-02 | Down-regulated |
| 1013 | hsa_circ_0020749 | -1.245 | 4.90E-02 | Down-regulated |
| 1014 | hsa_circ_0091934 | -1.245 | 2.20E-02 | Down-regulated |
| 1015 | hsa_circ_0000433 | -1.246 | 1.70E-02 | Down-regulated |
| 1016 | hsa_circ_0008653 | -1.246 | 3.40E-02 | Down-regulated |
| 1017 | hsa_circ_0002314 | -1.246 | 4.00E-03 | Down-regulated |
| 1018 | hsa_circ_0007259 | -1.246 | 1.90E-02 | Down-regulated |
| 1019 | hsa_circ_0006675 | -1.246 | 3.40E-02 | Down-regulated |
| 1020 | hsa_circ_0006823 | -1.246 | 3.20E-02 | Down-regulated |
| 1021 | hsa_circ_0066985 | -1.247 | 2.60E-02 | Down-regulated |
| 1022 | hsa_circ_0006898 | -1.247 | 2.90E-02 | Down-regulated |
| 1023 | hsa_circ_0002744 | -1.247 | 3.50E-02 | Down-regulated |
| 1024 | hsa_circ_0079614 | -1.248 | 1.60E-02 | Down-regulated |
| 1025 | hsa_circ_0007245 | -1.248 | 2.20E-02 | Down-regulated |
| 1026 | hsa_circ_0002798 | -1.248 | 2.10E-02 | Down-regulated |
| 1027 | hsa_circ_0008878 | -1.248 | 1.10E-02 | Down-regulated |
| 1028 | hsa_circ_0054272 | -1.249 | 3.00E-02 | Down-regulated |
| 1029 | hsa_circ_0002569 | -1.249 | 2.50E-02 | Down-regulated |
| 1030 | hsa_circ_0004913 | -1.250 | 1.60E-02 | Down-regulated |
| 1031 | hsa_circ_0001766 | -1.250 | 3.80E-02 | Down-regulated |
| 1032 | hsa_circ_0055038 | -1.250 | 1.80E-02 | Down-regulated |
| 1033 | hsa_circ_0064022 | -1.250 | 1.30E-02 | Down-regulated |
| 1034 | hsa_circ_0066644 | -1.251 | 2.00E-03 | Down-regulated |
| 1035 | hsa_circ_0071236 | -1.251 | 1.80E-02 | Down-regulated |
| 1036 | hsa_circ_0042368 | -1.251 | 4.20E-02 | Down-regulated |
| 1037 | hsa_circ_0008704 | -1.251 | 2.60E-02 | Down-regulated |
| 1038 | hsa_circ_0016503 | -1.252 | 2.30E-02 | Down-regulated |
| 1039 | hsa_circ_0059910 | -1.252 | 1.90E-02 | Down-regulated |
| 1040 | hsa_circ_0075281 | -1.252 | 2.60E-02 | Down-regulated |
| 1041 | hsa_circ_0085187 | -1.253 | 2.50E-02 | Down-regulated |
| 1042 | hsa_circ_0079939 | -1.254 | 1.20E-02 | Down-regulated |
| 1043 | hsa_circ_0012871 | -1.256 | 3.10E-02 | Down-regulated |
| 1044 | hsa_circ_0001459 | -1.256 | 1.70E-02 | Down-regulated |
| 1045 | hsa_circ_0018466 | -1.257 | 3.50E-02 | Down-regulated |
| 1046 | hsa_circ_0077279 | -1.258 | 2.70E-02 | Down-regulated |
| 1047 | hsa_circ_0030239 | -1.260 | 4.90E-02 | Down-regulated |
| 1048 | hsa_circ_0053932 | -1.260 | 6.00E-03 | Down-regulated |
| 1049 | hsa_circ_0051218 | -1.261 | 2.40E-02 | Down-regulated |
| 1050 | hsa_circ_0007083 | -1.261 | 1.40E-02 | Down-regulated |
| 1051 | hsa_circ_0005918 | -1.261 | 1.10E-02 | Down-regulated |
| 1052 | hsa_circ_0027902 | -1.261 | 1.50E-02 | Down-regulated |
| 1053 | hsa_circ_0058175 | -1.262 | 1.90E-02 | Down-regulated |
| 1054 | hsa_circ_0005519 | -1.262 | 2.60E-02 | Down-regulated |
| 1055 | hsa_circ_0062439 | -1.262 | 4.00E-02 | Down-regulated |
| 1056 | hsa_circ_0084353 | -1.263 | 2.80E-02 | Down-regulated |
| 1057 | hsa_circ_0069244 | -1.263 | 1.40E-02 | Down-regulated |
| 1058 | hsa_circ_0009118 | -1.263 | 4.70E-02 | Down-regulated |
| 1059 | hsa_circ_0021928 | -1.263 | 2.30E-02 | Down-regulated |
| 1060 | hsa_circ_0000209 | -1.264 | 1.80E-02 | Down-regulated |
| 1061 | hsa_circ_0020169 | -1.264 | 1.50E-02 | Down-regulated |
| 1062 | hsa_circ_0084583 | -1.264 | 3.00E-02 | Down-regulated |
| 1063 | hsa_circ_0006669 | -1.264 | 3.40E-02 | Down-regulated |
| 1064 | hsa_circ_0028147 | -1.264 | 1.70E-02 | Down-regulated |
| 1065 | hsa_circ_0023224 | -1.265 | 1.80E-02 | Down-regulated |
| 1066 | hsa_circ_0056189 | -1.266 | 2.00E-02 | Down-regulated |
| 1067 | hsa_circ_0005567 | -1.266 | 2.50E-02 | Down-regulated |
| 1068 | hsa_circ_0055243 | -1.266 | 2.00E-02 | Down-regulated |
| 1069 | hsa_circ_0054877 | -1.266 | 3.50E-02 | Down-regulated |
| 1070 | hsa_circ_0046971 | -1.266 | 2.00E-02 | Down-regulated |
| 1071 | hsa_circ_0027684 | -1.267 | 4.00E-02 | Down-regulated |
| 1072 | hsa_circ_0055148 | -1.268 | 3.10E-02 | Down-regulated |
| 1073 | hsa_circ_0076337 | -1.268 | 1.20E-02 | Down-regulated |
| 1074 | hsa_circ_0003323 | -1.268 | 3.00E-02 | Down-regulated |
| 1075 | hsa_circ_0000109 | -1.269 | 4.20E-02 | Down-regulated |
| 1076 | hsa_circ_0007602 | -1.269 | 1.50E-02 | Down-regulated |
| 1077 | hsa_circ_0003640 | -1.269 | 1.70E-02 | Down-regulated |
| 1078 | hsa_circ_0008083 | -1.270 | 1.70E-02 | Down-regulated |
| 1079 | hsa_circ_0003988 | -1.270 | 1.10E-02 | Down-regulated |
| 1080 | hsa_circ_0008898 | -1.270 | 2.50E-02 | Down-regulated |
| 1081 | hsa_circ_0025946 | -1.271 | 4.70E-02 | Down-regulated |
| 1082 | hsa_circ_0025202 | -1.271 | 2.70E-02 | Down-regulated |
| 1083 | hsa_circ_0087004 | -1.271 | 1.70E-02 | Down-regulated |
| 1084 | hsa_circ_0021934 | -1.272 | 6.00E-03 | Down-regulated |
| 1085 | hsa_circ_0082335 | -1.272 | 1.40E-02 | Down-regulated |
| 1086 | hsa_circ_0064195 | -1.273 | 3.50E-02 | Down-regulated |
| 1087 | hsa_circ_0008997 | -1.273 | 3.60E-02 | Down-regulated |
| 1088 | hsa_circ_0090237 | -1.274 | 3.00E-02 | Down-regulated |
| 1089 | hsa_circ_0023918 | -1.275 | 3.00E-02 | Down-regulated |
| 1090 | hsa_circ_0074419 | -1.276 | 2.80E-02 | Down-regulated |
| 1091 | hsa_circ_0088373 | -1.276 | 2.80E-02 | Down-regulated |
| 1092 | hsa_circ_0047288 | -1.276 | 8.00E-03 | Down-regulated |
| 1093 | hsa_circ_0005104 | -1.276 | 2.90E-02 | Down-regulated |
| 1094 | hsa_circ_0003633 | -1.276 | 2.80E-02 | Down-regulated |
| 1095 | hsa_circ_0079449 | -1.277 | 1.30E-02 | Down-regulated |
| 1096 | hsa_circ_0001965 | -1.277 | 3.40E-02 | Down-regulated |
| 1097 | hsa_circ_0085509 | -1.278 | 2.40E-02 | Down-regulated |
| 1098 | hsa_circ_0070613 | -1.279 | 1.80E-02 | Down-regulated |
| 1099 | hsa_circ_0072264 | -1.279 | 1.70E-02 | Down-regulated |
| 1100 | hsa_circ_0005726 | -1.279 | 1.50E-02 | Down-regulated |
| 1101 | hsa_circ_0001491 | -1.280 | 9.00E-03 | Down-regulated |
| 1102 | hsa_circ_0001085 | -1.280 | 2.10E-02 | Down-regulated |
| 1103 | hsa_circ_0006436 | -1.280 | 1.50E-02 | Down-regulated |
| 1104 | hsa_circ_0021464 | -1.280 | 1.40E-02 | Down-regulated |
| 1105 | hsa_circ_0005863 | -1.280 | 2.50E-02 | Down-regulated |
| 1106 | hsa_circ_0088070 | -1.281 | 4.30E-02 | Down-regulated |
| 1107 | hsa_circ_0007443 | -1.281 | 2.40E-02 | Down-regulated |
| 1108 | hsa_circ_0009057 | -1.281 | 1.00E-02 | Down-regulated |
| 1109 | hsa_circ_0001147 | -1.282 | 4.30E-02 | Down-regulated |
| 1110 | hsa_circ_0078368 | -1.282 | 4.20E-02 | Down-regulated |
| 1111 | hsa_circ_0026885 | -1.283 | 3.40E-02 | Down-regulated |
| 1112 | hsa_circ_0043446 | -1.283 | 3.80E-02 | Down-regulated |
| 1113 | hsa_circ_0082141 | -1.283 | 2.70E-02 | Down-regulated |
| 1114 | hsa_circ_0049886 | -1.284 | 4.20E-02 | Down-regulated |
| 1115 | hsa_circ_0001612 | -1.284 | 3.30E-02 | Down-regulated |
| 1116 | hsa_circ_0007457 | -1.284 | 2.60E-02 | Down-regulated |
| 1117 | hsa_circ_0004886 | -1.284 | 4.10E-02 | Down-regulated |
| 1118 | hsa_circ_0064687 | -1.285 | 2.10E-02 | Down-regulated |
| 1119 | hsa_circ_0069811 | -1.285 | 3.70E-02 | Down-regulated |
| 1120 | hsa_circ_0002433 | -1.285 | 2.40E-02 | Down-regulated |
| 1121 | hsa_circ_0037760 | -1.286 | 4.00E-02 | Down-regulated |
| 1122 | hsa_circ_0086277 | -1.286 | 3.20E-02 | Down-regulated |
| 1123 | hsa_circ_0001314 | -1.287 | 1.50E-02 | Down-regulated |
| 1124 | hsa_circ_0075761 | -1.287 | 7.00E-03 | Down-regulated |
| 1125 | hsa_circ_0047878 | -1.288 | 3.10E-02 | Down-regulated |
| 1126 | hsa_circ_0006745 | -1.289 | 1.60E-02 | Down-regulated |
| 1127 | hsa_circ_0007958 | -1.289 | 7.00E-03 | Down-regulated |
| 1128 | hsa_circ_0015839 | -1.289 | 2.10E-02 | Down-regulated |
| 1129 | hsa_circ_0067204 | -1.289 | 1.10E-02 | Down-regulated |
| 1130 | hsa_circ_0007808 | -1.290 | 2.30E-02 | Down-regulated |
| 1131 | hsa_circ_0073927 | -1.290 | 3.60E-02 | Down-regulated |
| 1132 | hsa_circ_0076199 | -1.291 | 9.00E-03 | Down-regulated |
| 1133 | hsa_circ_0010466 | -1.291 | 1.20E-02 | Down-regulated |
| 1134 | hsa_circ_0074806 | -1.291 | 2.90E-02 | Down-regulated |
| 1135 | hsa_circ_0053375 | -1.292 | 7.00E-03 | Down-regulated |
| 1136 | hsa_circ_0021583 | -1.293 | 2.10E-02 | Down-regulated |
| 1137 | hsa_circ_0001650 | -1.294 | 1.40E-02 | Down-regulated |
| 1138 | hsa_circ_0084151 | -1.294 | 3.80E-02 | Down-regulated |
| 1139 | hsa_circ_0012969 | -1.295 | 2.40E-02 | Down-regulated |
| 1140 | hsa_circ_0008081 | -1.295 | 2.60E-02 | Down-regulated |
| 1141 | hsa_circ_0044916 | -1.296 | 1.10E-02 | Down-regulated |
| 1142 | hsa_circ_0007504 | -1.297 | 2.30E-02 | Down-regulated |
| 1143 | hsa_circ_0047419 | -1.298 | 9.00E-03 | Down-regulated |
| 1144 | hsa_circ_0007746 | -1.299 | 1.90E-02 | Down-regulated |
| 1145 | hsa_circ_0090410 | -1.300 | 2.00E-02 | Down-regulated |
| 1146 | hsa_circ_0031288 | -1.300 | 3.90E-02 | Down-regulated |
| 1147 | hsa_circ_0000430 | -1.300 | 3.60E-02 | Down-regulated |
| 1148 | hsa_circ_0004457 | -1.300 | 4.70E-02 | Down-regulated |
| 1149 | hsa_circ_0084443 | -1.301 | 3.60E-02 | Down-regulated |
| 1150 | hsa_circ_0085278 | -1.301 | 3.70E-02 | Down-regulated |
| 1151 | hsa_circ_0003650 | -1.301 | 2.50E-02 | Down-regulated |
| 1152 | hsa_circ_0067550 | -1.301 | 1.30E-02 | Down-regulated |
| 1153 | hsa_circ_0005328 | -1.302 | 2.90E-02 | Down-regulated |
| 1154 | hsa_circ_0000028 | -1.302 | 3.00E-02 | Down-regulated |
| 1155 | hsa_circ_0072083 | -1.302 | 1.90E-02 | Down-regulated |
| 1156 | hsa_circ_0082626 | -1.302 | 4.20E-02 | Down-regulated |
| 1157 | hsa_circ_0045128 | -1.303 | 4.20E-02 | Down-regulated |
| 1158 | hsa_circ_0056519 | -1.303 | 3.10E-02 | Down-regulated |
| 1159 | hsa_circ_0058451 | -1.303 | 3.90E-02 | Down-regulated |
| 1160 | hsa_circ_0002665 | -1.303 | 2.00E-02 | Down-regulated |
| 1161 | hsa_circ_0054656 | -1.304 | 2.40E-02 | Down-regulated |
| 1162 | hsa_circ_0069338 | -1.304 | 2.70E-02 | Down-regulated |
| 1163 | hsa_circ_0017835 | -1.304 | 2.80E-02 | Down-regulated |
| 1164 | hsa_circ_0019104 | -1.305 | 4.00E-02 | Down-regulated |
| 1165 | hsa_circ_0017359 | -1.306 | 1.30E-02 | Down-regulated |
| 1166 | hsa_circ_0013561 | -1.306 | 3.70E-02 | Down-regulated |
| 1167 | hsa_circ_0060255 | -1.307 | 2.40E-02 | Down-regulated |
| 1168 | hsa_circ_0007961 | -1.307 | 3.60E-02 | Down-regulated |
| 1169 | hsa_circ_0003393 | -1.307 | 3.40E-02 | Down-regulated |
| 1170 | hsa_circ_0066631 | -1.308 | 2.30E-02 | Down-regulated |
| 1171 | hsa_circ_0022803 | -1.308 | 2.70E-02 | Down-regulated |
| 1172 | hsa_circ_0054654 | -1.308 | 2.40E-02 | Down-regulated |
| 1173 | hsa_circ_0000283 | -1.308 | 1.30E-02 | Down-regulated |
| 1174 | hsa_circ_0001539 | -1.309 | 2.90E-02 | Down-regulated |
| 1175 | hsa_circ_0073441 | -1.309 | 3.00E-02 | Down-regulated |
| 1176 | hsa_circ_0069749 | -1.309 | 3.80E-02 | Down-regulated |
| 1177 | hsa_circ_0016650 | -1.309 | 1.50E-02 | Down-regulated |
| 1178 | hsa_circ_0014923 | -1.310 | 2.60E-02 | Down-regulated |
| 1179 | hsa_circ_0048344 | -1.310 | 1.90E-02 | Down-regulated |
| 1180 | hsa_circ_0008062 | -1.310 | 3.60E-02 | Down-regulated |
| 1181 | hsa_circ_0061396 | -1.310 | 4.10E-02 | Down-regulated |
| 1182 | hsa_circ_0027719 | -1.312 | 3.30E-02 | Down-regulated |
| 1183 | hsa_circ_0075705 | -1.313 | 4.70E-02 | Down-regulated |
| 1184 | hsa_circ_0008491 | -1.313 | 1.50E-02 | Down-regulated |
| 1185 | hsa_circ_0073983 | -1.313 | 1.30E-02 | Down-regulated |
| 1186 | hsa_circ_0023920 | -1.314 | 2.10E-02 | Down-regulated |
| 1187 | hsa_circ_0000498 | -1.314 | 6.00E-03 | Down-regulated |
| 1188 | hsa_circ_0038465 | -1.314 | 6.00E-03 | Down-regulated |
| 1189 | hsa_circ_0078510 | -1.314 | 3.90E-02 | Down-regulated |
| 1190 | hsa_circ_0036249 | -1.314 | 3.10E-02 | Down-regulated |
| 1191 | hsa_circ_0007254 | -1.315 | 4.10E-02 | Down-regulated |
| 1192 | hsa_circ_0004050 | -1.315 | 2.40E-02 | Down-regulated |
| 1193 | hsa_circ_0046050 | -1.315 | 9.00E-03 | Down-regulated |
| 1194 | hsa_circ_0008813 | -1.315 | 1.40E-02 | Down-regulated |
| 1195 | hsa_circ_0031607 | -1.316 | 8.00E-03 | Down-regulated |
| 1196 | hsa_circ_0072654 | -1.317 | 1.40E-02 | Down-regulated |
| 1197 | hsa_circ_0072305 | -1.318 | 1.10E-02 | Down-regulated |
| 1198 | hsa_circ_0054682 | -1.319 | 1.60E-02 | Down-regulated |
| 1199 | hsa_circ_0067354 | -1.320 | 2.30E-02 | Down-regulated |
| 1200 | hsa_circ_0044185 | -1.320 | 1.60E-02 | Down-regulated |
| 1201 | hsa_circ_0000718 | -1.321 | 3.60E-02 | Down-regulated |
| 1202 | hsa_circ_0001921 | -1.321 | 2.00E-02 | Down-regulated |
| 1203 | hsa_circ_0060733 | -1.322 | 4.50E-02 | Down-regulated |
| 1204 | hsa_circ_0068291 | -1.323 | 2.00E-02 | Down-regulated |
| 1205 | hsa_circ_0001180 | -1.323 | 3.60E-02 | Down-regulated |
| 1206 | hsa_circ_0054101 | -1.323 | 3.00E-02 | Down-regulated |
| 1207 | hsa_circ_0005840 | -1.324 | 8.00E-03 | Down-regulated |
| 1208 | hsa_circ_0081746 | -1.325 | 1.80E-02 | Down-regulated |
| 1209 | hsa_circ_0004665 | -1.325 | 3.40E-02 | Down-regulated |
| 1210 | hsa_circ_0002058 | -1.325 | 3.00E-02 | Down-regulated |
| 1211 | hsa_circ_0021822 | -1.325 | 1.50E-02 | Down-regulated |
| 1212 | hsa_circ_0085053 | -1.326 | 2.20E-02 | Down-regulated |
| 1213 | hsa_circ_0003310 | -1.327 | 3.80E-02 | Down-regulated |
| 1214 | hsa_circ_0065149 | -1.327 | 1.90E-02 | Down-regulated |
| 1215 | hsa_circ_0078610 | -1.327 | 1.90E-02 | Down-regulated |
| 1216 | hsa_circ_0002922 | -1.327 | 8.00E-03 | Down-regulated |
| 1217 | hsa_circ_0075172 | -1.327 | 4.30E-02 | Down-regulated |
| 1218 | hsa_circ_0082029 | -1.328 | 3.00E-02 | Down-regulated |
| 1219 | hsa_circ_0041630 | -1.328 | 2.20E-02 | Down-regulated |
| 1220 | hsa_circ_0066814 | -1.328 | 3.70E-02 | Down-regulated |
| 1221 | hsa_circ_0003590 | -1.328 | 1.40E-02 | Down-regulated |
| 1222 | hsa_circ_0056542 | -1.329 | 1.00E-02 | Down-regulated |
| 1223 | hsa_circ_0070649 | -1.330 | 1.60E-02 | Down-regulated |
| 1224 | hsa_circ_0001325 | -1.330 | 1.50E-02 | Down-regulated |
| 1225 | hsa_circ_0003949 | -1.330 | 1.60E-02 | Down-regulated |
| 1226 | hsa_circ_0030432 | -1.331 | 6.00E-03 | Down-regulated |
| 1227 | hsa_circ_0074410 | -1.331 | 2.60E-02 | Down-regulated |
| 1228 | hsa_circ_0084846 | -1.331 | 1.30E-02 | Down-regulated |
| 1229 | hsa_circ_0015513 | -1.331 | 1.90E-02 | Down-regulated |
| 1230 | hsa_circ_0066083 | -1.332 | 3.60E-02 | Down-regulated |
| 1231 | hsa_circ_0035946 | -1.333 | 3.20E-02 | Down-regulated |
| 1232 | hsa_circ_0036068 | -1.333 | 9.00E-03 | Down-regulated |
| 1233 | hsa_circ_0008620 | -1.333 | 1.70E-02 | Down-regulated |
| 1234 | hsa_circ_0006712 | -1.334 | 1.40E-02 | Down-regulated |
| 1235 | hsa_circ_0076143 | -1.334 | 3.00E-03 | Down-regulated |
| 1236 | hsa_circ_0070190 | -1.334 | 2.10E-02 | Down-regulated |
| 1237 | hsa_circ_0047427 | -1.334 | 1.50E-02 | Down-regulated |
| 1238 | hsa_circ_0070574 | -1.334 | 1.50E-02 | Down-regulated |
| 1239 | hsa_circ_0004879 | -1.335 | 2.80E-02 | Down-regulated |
| 1240 | hsa_circ_0000837 | -1.335 | 2.00E-02 | Down-regulated |
| 1241 | hsa_circ_0013054 | -1.335 | 1.40E-02 | Down-regulated |
| 1242 | hsa_circ_0000296 | -1.335 | 2.10E-02 | Down-regulated |
| 1243 | hsa_circ_0027545 | -1.336 | 1.60E-02 | Down-regulated |
| 1244 | hsa_circ_0017988 | -1.337 | 1.40E-02 | Down-regulated |
| 1245 | hsa_circ_0091183 | -1.337 | 1.30E-02 | Down-regulated |
| 1246 | hsa_circ_0009058 | -1.337 | 1.10E-02 | Down-regulated |
| 1247 | hsa_circ_0009092 | -1.338 | 2.40E-02 | Down-regulated |
| 1248 | hsa_circ_0026722 | -1.338 | 8.00E-03 | Down-regulated |
| 1249 | hsa_circ_0003474 | -1.338 | 3.80E-02 | Down-regulated |
| 1250 | hsa_circ_0001524 | -1.340 | 1.90E-02 | Down-regulated |
| 1251 | hsa_circ_0024920 | -1.340 | 2.10E-02 | Down-regulated |
| 1252 | hsa_circ_0002359 | -1.340 | 2.70E-02 | Down-regulated |
| 1253 | hsa_circ_0008657 | -1.340 | 1.20E-02 | Down-regulated |
| 1254 | hsa_circ_0011153 | -1.340 | 3.70E-02 | Down-regulated |
| 1255 | hsa_circ_0082131 | -1.342 | 2.70E-02 | Down-regulated |
| 1256 | hsa_circ_0012967 | -1.343 | 4.40E-02 | Down-regulated |
| 1257 | hsa_circ_0070934 | -1.343 | 4.20E-02 | Down-regulated |
| 1258 | hsa_circ_0000347 | -1.343 | 2.90E-02 | Down-regulated |
| 1259 | hsa_circ_0008321 | -1.345 | 3.00E-02 | Down-regulated |
| 1260 | hsa_circ_0071023 | -1.345 | 4.90E-02 | Down-regulated |
| 1261 | hsa_circ_0083834 | -1.345 | 1.00E-02 | Down-regulated |
| 1262 | hsa_circ_0037849 | -1.345 | 1.00E-03 | Down-regulated |
| 1263 | hsa_circ_0042806 | -1.345 | 3.00E-02 | Down-regulated |
| 1264 | hsa_circ_0017099 | -1.345 | 2.20E-02 | Down-regulated |
| 1265 | hsa_circ_0002496 | -1.346 | 1.70E-02 | Down-regulated |
| 1266 | hsa_circ_0030378 | -1.346 | 1.60E-02 | Down-regulated |
| 1267 | hsa_circ_0009246 | -1.346 | 4.30E-02 | Down-regulated |
| 1268 | hsa_circ_0031646 | -1.348 | 1.60E-02 | Down-regulated |
| 1269 | hsa_circ_0060521 | -1.348 | 2.00E-02 | Down-regulated |
| 1270 | hsa_circ_0073128 | -1.348 | 1.30E-02 | Down-regulated |
| 1271 | hsa_circ_0025476 | -1.348 | 2.30E-02 | Down-regulated |
| 1272 | hsa_circ_0035265 | -1.348 | 3.00E-02 | Down-regulated |
| 1273 | hsa_circ_0006490 | -1.349 | 3.80E-02 | Down-regulated |
| 1274 | hsa_circ_0068629 | -1.349 | 2.30E-02 | Down-regulated |
| 1275 | hsa_circ_0069582 | -1.349 | 2.70E-02 | Down-regulated |
| 1276 | hsa_circ_0077228 | -1.349 | 1.70E-02 | Down-regulated |
| 1277 | hsa_circ_0014887 | -1.349 | 3.90E-02 | Down-regulated |
| 1278 | hsa_circ_0030050 | -1.350 | 2.00E-02 | Down-regulated |
| 1279 | hsa_circ_0071984 | -1.350 | 1.90E-02 | Down-regulated |
| 1280 | hsa_circ_0001614 | -1.351 | 1.90E-02 | Down-regulated |
| 1281 | hsa_circ_0003865 | -1.351 | 9.00E-03 | Down-regulated |
| 1282 | hsa_circ_0029636 | -1.351 | 1.70E-02 | Down-regulated |
| 1283 | hsa_circ_0001278 | -1.352 | 4.10E-02 | Down-regulated |
| 1284 | hsa_circ_0000251 | -1.352 | 4.40E-02 | Down-regulated |
| 1285 | hsa_circ_0001581 | -1.352 | 1.00E-02 | Down-regulated |
| 1286 | hsa_circ_0018008 | -1.353 | 4.10E-02 | Down-regulated |
| 1287 | hsa_circ_0025554 | -1.353 | 2.90E-02 | Down-regulated |
| 1288 | hsa_circ_0000859 | -1.354 | 1.90E-02 | Down-regulated |
| 1289 | hsa_circ_0006258 | -1.354 | 1.30E-02 | Down-regulated |
| 1290 | hsa_circ_0003722 | -1.355 | 9.00E-03 | Down-regulated |
| 1291 | hsa_circ_0014877 | -1.355 | 3.90E-02 | Down-regulated |
| 1292 | hsa_circ_0001521 | -1.356 | 4.10E-02 | Down-regulated |
| 1293 | hsa_circ_0007557 | -1.356 | 1.40E-02 | Down-regulated |
| 1294 | hsa_circ_0054700 | -1.357 | 1.80E-02 | Down-regulated |
| 1295 | hsa_circ_0014202 | -1.357 | 3.00E-02 | Down-regulated |
| 1296 | hsa_circ_0001574 | -1.357 | 2.80E-02 | Down-regulated |
| 1297 | hsa_circ_0074167 | -1.358 | 4.60E-02 | Down-regulated |
| 1298 | hsa_circ_0003625 | -1.358 | 2.00E-02 | Down-regulated |
| 1299 | hsa_circ_0084679 | -1.358 | 2.40E-02 | Down-regulated |
| 1300 | hsa_circ_0089933 | -1.359 | 3.20E-02 | Down-regulated |
| 1301 | hsa_circ_0051806 | -1.359 | 8.00E-03 | Down-regulated |
| 1302 | hsa_circ_0073539 | -1.359 | 2.30E-02 | Down-regulated |
| 1303 | hsa_circ_0068708 | -1.359 | 4.50E-02 | Down-regulated |
| 1304 | hsa_circ_0002594 | -1.360 | 3.40E-02 | Down-regulated |
| 1305 | hsa_circ_0002039 | -1.361 | 2.00E-02 | Down-regulated |
| 1306 | hsa_circ_0069570 | -1.361 | 4.70E-02 | Down-regulated |
| 1307 | hsa_circ_0023944 | -1.362 | 2.20E-02 | Down-regulated |
| 1308 | hsa_circ_0023891 | -1.362 | 2.70E-02 | Down-regulated |
| 1309 | hsa_circ_0006505 | -1.362 | 1.00E-02 | Down-regulated |
| 1310 | hsa_circ_0076313 | -1.363 | 1.60E-02 | Down-regulated |
| 1311 | hsa_circ_0028057 | -1.363 | 2.60E-02 | Down-regulated |
| 1312 | hsa_circ_0008672 | -1.363 | 1.90E-02 | Down-regulated |
| 1313 | hsa_circ_0087008 | -1.363 | 2.80E-02 | Down-regulated |
| 1314 | hsa_circ_0007383 | -1.364 | 3.90E-02 | Down-regulated |
| 1315 | hsa_circ_0002773 | -1.364 | 8.00E-03 | Down-regulated |
| 1316 | hsa_circ_0005129 | -1.365 | 1.20E-02 | Down-regulated |
| 1317 | hsa_circ_0006101 | -1.365 | 2.30E-02 | Down-regulated |
| 1318 | hsa_circ_0001152 | -1.366 | 3.70E-02 | Down-regulated |
| 1319 | hsa_circ_0084275 | -1.366 | 1.50E-02 | Down-regulated |
| 1320 | hsa_circ_0072430 | -1.366 | 3.20E-02 | Down-regulated |
| 1321 | hsa_circ_0017037 | -1.366 | 1.50E-02 | Down-regulated |
| 1322 | hsa_circ_0004116 | -1.367 | 3.80E-02 | Down-regulated |
| 1323 | hsa_circ_0004346 | -1.367 | 1.50E-02 | Down-regulated |
| 1324 | hsa_circ_0053141 | -1.368 | 4.00E-03 | Down-regulated |
| 1325 | hsa_circ_0004950 | -1.369 | 3.60E-02 | Down-regulated |
| 1326 | hsa_circ_0007401 | -1.369 | 4.50E-02 | Down-regulated |
| 1327 | hsa_circ_0069352 | -1.370 | 1.90E-02 | Down-regulated |
| 1328 | hsa_circ_0051805 | -1.370 | 7.00E-03 | Down-regulated |
| 1329 | hsa_circ_0018657 | -1.372 | 4.70E-02 | Down-regulated |
| 1330 | hsa_circ_0036347 | -1.372 | 1.90E-02 | Down-regulated |
| 1331 | hsa_circ_0026023 | -1.372 | 1.10E-02 | Down-regulated |
| 1332 | hsa_circ_0019646 | -1.372 | 2.80E-02 | Down-regulated |
| 1333 | hsa_circ_0087820 | -1.372 | 3.50E-02 | Down-regulated |
| 1334 | hsa_circ_0003613 | -1.372 | 4.20E-02 | Down-regulated |
| 1335 | hsa_circ_0001287 | -1.372 | 6.00E-03 | Down-regulated |
| 1336 | hsa_circ_0010931 | -1.372 | 2.90E-02 | Down-regulated |
| 1337 | hsa_circ_0024130 | -1.373 | 1.80E-02 | Down-regulated |
| 1338 | hsa_circ_0005232 | -1.374 | 2.40E-02 | Down-regulated |
| 1339 | hsa_circ_0024293 | -1.374 | 1.50E-02 | Down-regulated |
| 1340 | hsa_circ_0088333 | -1.374 | 6.00E-03 | Down-regulated |
| 1341 | hsa_circ_0059060 | -1.376 | 2.40E-02 | Down-regulated |
| 1342 | hsa_circ_0075158 | -1.376 | 2.10E-02 | Down-regulated |
| 1343 | hsa_circ_0053372 | -1.377 | 2.10E-02 | Down-regulated |
| 1344 | hsa_circ_0078780 | -1.377 | 1.70E-02 | Down-regulated |
| 1345 | hsa_circ_0032988 | -1.377 | 2.50E-02 | Down-regulated |
| 1346 | hsa_circ_0029775 | -1.377 | 2.30E-02 | Down-regulated |
| 1347 | hsa_circ_0017772 | -1.378 | 2.20E-02 | Down-regulated |
| 1348 | hsa_circ_0045537 | -1.378 | 9.00E-03 | Down-regulated |
| 1349 | hsa_circ_0000651 | -1.378 | 4.60E-02 | Down-regulated |
| 1350 | hsa_circ_0022102 | -1.378 | 2.80E-02 | Down-regulated |
| 1351 | hsa_circ_0004270 | -1.378 | 1.00E-02 | Down-regulated |
| 1352 | hsa_circ_0005415 | -1.379 | 2.60E-02 | Down-regulated |
| 1353 | hsa_circ_0013167 | -1.379 | 9.00E-03 | Down-regulated |
| 1354 | hsa_circ_0047728 | -1.379 | 8.00E-03 | Down-regulated |
| 1355 | hsa_circ_0067406 | -1.380 | 3.40E-02 | Down-regulated |
| 1356 | hsa_circ_0003549 | -1.380 | 1.80E-02 | Down-regulated |
| 1357 | hsa_circ_0084150 | -1.380 | 3.00E-02 | Down-regulated |
| 1358 | hsa_circ_0068461 | -1.380 | 2.70E-02 | Down-regulated |
| 1359 | hsa_circ_0007591 | -1.381 | 3.30E-02 | Down-regulated |
| 1360 | hsa_circ_0064567 | -1.381 | 7.00E-03 | Down-regulated |
| 1361 | hsa_circ_0001039 | -1.383 | 2.70E-02 | Down-regulated |
| 1362 | hsa_circ_0006395 | -1.383 | 1.70E-02 | Down-regulated |
| 1363 | hsa_circ_0005192 | -1.384 | 1.60E-02 | Down-regulated |
| 1364 | hsa_circ_0028201 | -1.384 | 7.00E-03 | Down-regulated |
| 1365 | hsa_circ_0007608 | -1.385 | 3.00E-02 | Down-regulated |
| 1366 | hsa_circ_0001324 | -1.385 | 2.30E-02 | Down-regulated |
| 1367 | hsa_circ_0045579 | -1.385 | 4.10E-02 | Down-regulated |
| 1368 | hsa_circ_0001685 | -1.385 | 1.50E-02 | Down-regulated |
| 1369 | hsa_circ_0082546 | -1.385 | 2.10E-02 | Down-regulated |
| 1370 | hsa_circ_0066768 | -1.386 | 1.30E-02 | Down-regulated |
| 1371 | hsa_circ_0015261 | -1.388 | 3.60E-02 | Down-regulated |
| 1372 | hsa_circ_0043122 | -1.388 | 1.70E-02 | Down-regulated |
| 1373 | hsa_circ_0042459 | -1.388 | 1.90E-02 | Down-regulated |
| 1374 | hsa_circ_0034294 | -1.388 | 2.40E-02 | Down-regulated |
| 1375 | hsa_circ_0001543 | -1.388 | 3.40E-02 | Down-regulated |
| 1376 | hsa_circ_0005828 | -1.389 | 3.70E-02 | Down-regulated |
| 1377 | hsa_circ_0000762 | -1.389 | 1.70E-02 | Down-regulated |
| 1378 | hsa_circ_0069101 | -1.390 | 1.30E-02 | Down-regulated |
| 1379 | hsa_circ_0000596 | -1.390 | 4.60E-02 | Down-regulated |
| 1380 | hsa_circ_0028965 | -1.390 | 3.30E-02 | Down-regulated |
| 1381 | hsa_circ_0005732 | -1.390 | 3.40E-02 | Down-regulated |
| 1382 | hsa_circ_0004907 | -1.391 | 2.80E-02 | Down-regulated |
| 1383 | hsa_circ_0000564 | -1.392 | 3.50E-02 | Down-regulated |
| 1384 | hsa_circ_0002274 | -1.392 | 3.60E-02 | Down-regulated |
| 1385 | hsa_circ_0025608 | -1.392 | 8.00E-03 | Down-regulated |
| 1386 | hsa_circ_0003308 | -1.392 | 1.70E-02 | Down-regulated |
| 1387 | hsa_circ_0078363 | -1.392 | 3.70E-02 | Down-regulated |
| 1388 | hsa_circ_0074555 | -1.393 | 1.10E-02 | Down-regulated |
| 1389 | hsa_circ_0040639 | -1.394 | 5.00E-03 | Down-regulated |
| 1390 | hsa_circ_0058539 | -1.394 | 1.50E-02 | Down-regulated |
| 1391 | hsa_circ_0088021 | -1.394 | 2.80E-02 | Down-regulated |
| 1392 | hsa_circ_0001075 | -1.394 | 3.30E-02 | Down-regulated |
| 1393 | hsa_circ_0006947 | -1.394 | 4.40E-02 | Down-regulated |
| 1394 | hsa_circ_0008539 | -1.394 | 1.80E-02 | Down-regulated |
| 1395 | hsa_circ_0003508 | -1.394 | 3.70E-02 | Down-regulated |
| 1396 | hsa_circ_0000142 | -1.395 | 1.30E-02 | Down-regulated |
| 1397 | hsa_circ_0031739 | -1.395 | 3.90E-02 | Down-regulated |
| 1398 | hsa_circ_0056894 | -1.396 | 1.10E-02 | Down-regulated |
| 1399 | hsa_circ_0056790 | -1.397 | 4.60E-02 | Down-regulated |
| 1400 | hsa_circ_0063488 | -1.397 | 2.40E-02 | Down-regulated |
| 1401 | hsa_circ_0002098 | -1.397 | 1.10E-02 | Down-regulated |
| 1402 | hsa_circ_0034537 | -1.397 | 6.00E-03 | Down-regulated |
| 1403 | hsa_circ_0001348 | -1.398 | 4.80E-02 | Down-regulated |
| 1404 | hsa_circ_0069500 | -1.398 | 1.60E-02 | Down-regulated |
| 1405 | hsa_circ_0042049 | -1.399 | 5.00E-03 | Down-regulated |
| 1406 | hsa_circ_0064743 | -1.399 | 2.60E-02 | Down-regulated |
| 1407 | hsa_circ_0066078 | -1.399 | 1.40E-02 | Down-regulated |
| 1408 | hsa_circ_0000203 | -1.399 | 3.60E-02 | Down-regulated |
| 1409 | hsa_circ_0072711 | -1.399 | 1.70E-02 | Down-regulated |
| 1410 | hsa_circ_0036460 | -1.399 | 1.30E-02 | Down-regulated |
| 1411 | hsa_circ_0023942 | -1.399 | 1.80E-02 | Down-regulated |
| 1412 | hsa_circ_0000068 | -1.400 | 1.20E-02 | Down-regulated |
| 1413 | hsa_circ_0067864 | -1.400 | 3.40E-02 | Down-regulated |
| 1414 | hsa_circ_0054248 | -1.400 | 2.50E-02 | Down-regulated |
| 1415 | hsa_circ_0039844 | -1.402 | 1.70E-02 | Down-regulated |
| 1416 | hsa_circ_0005379 | -1.403 | 1.80E-02 | Down-regulated |
| 1417 | hsa_circ_0082081 | -1.403 | 1.30E-02 | Down-regulated |
| 1418 | hsa_circ_0036200 | -1.404 | 2.20E-02 | Down-regulated |
| 1419 | hsa_circ_0043781 | -1.404 | 3.60E-02 | Down-regulated |
| 1420 | hsa_circ_0006209 | -1.404 | 1.60E-02 | Down-regulated |
| 1421 | hsa_circ_0008755 | -1.406 | 3.80E-02 | Down-regulated |
| 1422 | hsa_circ_0035065 | -1.406 | 1.80E-02 | Down-regulated |
| 1423 | hsa_circ_0027727 | -1.406 | 1.50E-02 | Down-regulated |
| 1424 | hsa_circ_0000242 | -1.407 | 1.30E-02 | Down-regulated |
| 1425 | hsa_circ_0006732 | -1.408 | 1.70E-02 | Down-regulated |
| 1426 | hsa_circ_0005050 | -1.410 | 2.00E-02 | Down-regulated |
| 1427 | hsa_circ_0031583 | -1.410 | 4.00E-03 | Down-regulated |
| 1428 | hsa_circ_0002043 | -1.410 | 1.20E-02 | Down-regulated |
| 1429 | hsa_circ_0062901 | -1.411 | 2.20E-02 | Down-regulated |
| 1430 | hsa_circ_0027819 | -1.412 | 7.00E-03 | Down-regulated |
| 1431 | hsa_circ_0082547 | -1.412 | 1.10E-02 | Down-regulated |
| 1432 | hsa_circ_0000654 | -1.413 | 2.70E-02 | Down-regulated |
| 1433 | hsa_circ_0008586 | -1.415 | 4.20E-02 | Down-regulated |
| 1434 | hsa_circ_0042165 | -1.415 | 1.70E-02 | Down-regulated |
| 1435 | hsa_circ_0087429 | -1.415 | 2.30E-02 | Down-regulated |
| 1436 | hsa_circ_0082140 | -1.415 | 3.60E-02 | Down-regulated |
| 1437 | hsa_circ_0002607 | -1.416 | 1.50E-02 | Down-regulated |
| 1438 | hsa_circ_0006276 | -1.416 | 1.50E-02 | Down-regulated |
| 1439 | hsa_circ_0036103 | -1.416 | 1.10E-02 | Down-regulated |
| 1440 | hsa_circ_0001755 | -1.417 | 1.40E-02 | Down-regulated |
| 1441 | hsa_circ_0000222 | -1.417 | 4.50E-02 | Down-regulated |
| 1442 | hsa_circ_0001473 | -1.418 | 1.10E-02 | Down-regulated |
| 1443 | hsa_circ_0009000 | -1.420 | 3.00E-02 | Down-regulated |
| 1444 | hsa_circ_0002099 | -1.420 | 4.00E-03 | Down-regulated |
| 1445 | hsa_circ_0000994 | -1.421 | 2.70E-02 | Down-regulated |
| 1446 | hsa_circ_0021560 | -1.421 | 1.70E-02 | Down-regulated |
| 1447 | hsa_circ_0031851 | -1.423 | 8.00E-03 | Down-regulated |
| 1448 | hsa_circ_0018536 | -1.424 | 1.60E-02 | Down-regulated |
| 1449 | hsa_circ_0083469 | -1.424 | 4.80E-02 | Down-regulated |
| 1450 | hsa_circ_0070039 | -1.425 | 3.10E-02 | Down-regulated |
| 1451 | hsa_circ_0054970 | -1.425 | 1.20E-02 | Down-regulated |
| 1452 | hsa_circ_0021787 | -1.425 | 1.90E-02 | Down-regulated |
| 1453 | hsa_circ_0013494 | -1.426 | 3.90E-02 | Down-regulated |
| 1454 | hsa_circ_0008378 | -1.426 | 2.40E-02 | Down-regulated |
| 1455 | hsa_circ_0073997 | -1.426 | 3.10E-02 | Down-regulated |
| 1456 | hsa_circ_0052345 | -1.426 | 1.20E-02 | Down-regulated |
| 1457 | hsa_circ_0047677 | -1.426 | 3.40E-02 | Down-regulated |
| 1458 | hsa_circ_0015063 | -1.426 | 9.00E-03 | Down-regulated |
| 1459 | hsa_circ_0079352 | -1.428 | 2.40E-02 | Down-regulated |
| 1460 | hsa_circ_0032068 | -1.429 | 3.40E-02 | Down-regulated |
| 1461 | hsa_circ_0084514 | -1.429 | 5.00E-03 | Down-regulated |
| 1462 | hsa_circ_0052807 | -1.431 | 1.00E-02 | Down-regulated |
| 1463 | hsa_circ_0067434 | -1.431 | 1.70E-02 | Down-regulated |
| 1464 | hsa_circ_0014364 | -1.432 | 1.50E-02 | Down-regulated |
| 1465 | hsa_circ_0049963 | -1.432 | 2.10E-02 | Down-regulated |
| 1466 | hsa_circ_0000031 | -1.432 | 6.00E-03 | Down-regulated |
| 1467 | hsa_circ_0001263 | -1.432 | 4.40E-02 | Down-regulated |
| 1468 | hsa_circ_0046142 | -1.433 | 4.90E-02 | Down-regulated |
| 1469 | hsa_circ_0018242 | -1.433 | 2.80E-02 | Down-regulated |
| 1470 | hsa_circ_0006479 | -1.433 | 2.40E-02 | Down-regulated |
| 1471 | hsa_circ_0034551 | -1.433 | 2.60E-02 | Down-regulated |
| 1472 | hsa_circ_0032067 | -1.435 | 1.70E-02 | Down-regulated |
| 1473 | hsa_circ_0016113 | -1.436 | 1.70E-02 | Down-regulated |
| 1474 | hsa_circ_0004160 | -1.436 | 2.60E-02 | Down-regulated |
| 1475 | hsa_circ_0032812 | -1.437 | 1.90E-02 | Down-regulated |
| 1476 | hsa_circ_0036464 | -1.438 | 4.30E-02 | Down-regulated |
| 1477 | hsa_circ_0044248 | -1.438 | 3.00E-02 | Down-regulated |
| 1478 | hsa_circ_0086736 | -1.438 | 2.10E-02 | Down-regulated |
| 1479 | hsa_circ_0054547 | -1.438 | 4.40E-02 | Down-regulated |
| 1480 | hsa_circ_0039847 | -1.439 | 2.60E-02 | Down-regulated |
| 1481 | hsa_circ_0046141 | -1.440 | 3.60E-02 | Down-regulated |
| 1482 | hsa_circ_0023233 | -1.440 | 1.90E-02 | Down-regulated |
| 1483 | hsa_circ_0032275 | -1.440 | 2.10E-02 | Down-regulated |
| 1484 | hsa_circ_0053338 | -1.441 | 1.10E-02 | Down-regulated |
| 1485 | hsa_circ_0062688 | -1.441 | 2.80E-02 | Down-regulated |
| 1486 | hsa_circ_0025544 | -1.441 | 1.10E-02 | Down-regulated |
| 1487 | hsa_circ_0043249 | -1.442 | 2.50E-02 | Down-regulated |
| 1488 | hsa_circ_0001551 | -1.442 | 2.40E-02 | Down-regulated |
| 1489 | hsa_circ_0012448 | -1.442 | 1.90E-02 | Down-regulated |
| 1490 | hsa_circ_0001870 | -1.442 | 5.00E-03 | Down-regulated |
| 1491 | hsa_circ_0005493 | -1.443 | 3.20E-02 | Down-regulated |
| 1492 | hsa_circ_0000240 | -1.443 | 3.20E-02 | Down-regulated |
| 1493 | hsa_circ_0006989 | -1.444 | 2.80E-02 | Down-regulated |
| 1494 | hsa_circ_0053339 | -1.444 | 3.00E-03 | Down-regulated |
| 1495 | hsa_circ_0039454 | -1.445 | 7.00E-03 | Down-regulated |
| 1496 | hsa_circ_0000029 | -1.445 | 2.40E-02 | Down-regulated |
| 1497 | hsa_circ_0026700 | -1.445 | 6.00E-03 | Down-regulated |
| 1498 | hsa_circ_0031806 | -1.445 | 3.00E-02 | Down-regulated |
| 1499 | hsa_circ_0003615 | -1.445 | 3.40E-02 | Down-regulated |
| 1500 | hsa_circ_0000094 | -1.445 | 1.40E-02 | Down-regulated |
| 1501 | hsa_circ_0007458 | -1.446 | 2.80E-02 | Down-regulated |
| 1502 | hsa_circ_0008025 | -1.447 | 2.20E-02 | Down-regulated |
| 1503 | hsa_circ_0031710 | -1.447 | 1.20E-02 | Down-regulated |
| 1504 | hsa_circ_0079813 | -1.448 | 1.00E-02 | Down-regulated |
| 1505 | hsa_circ_0068040 | -1.448 | 3.00E-02 | Down-regulated |
| 1506 | hsa_circ_0085301 | -1.448 | 1.10E-02 | Down-regulated |
| 1507 | hsa_circ_0063369 | -1.449 | 8.00E-03 | Down-regulated |
| 1508 | hsa_circ_0007797 | -1.449 | 2.30E-02 | Down-regulated |
| 1509 | hsa_circ_0064742 | -1.450 | 2.60E-02 | Down-regulated |
| 1510 | hsa_circ_0002114 | -1.451 | 2.90E-02 | Down-regulated |
| 1511 | hsa_circ_0005209 | -1.451 | 3.90E-02 | Down-regulated |
| 1512 | hsa_circ_0001884 | -1.451 | 1.30E-02 | Down-regulated |
| 1513 | hsa_circ_0000992 | -1.452 | 2.10E-02 | Down-regulated |
| 1514 | hsa_circ_0039195 | -1.452 | 6.00E-03 | Down-regulated |
| 1515 | hsa_circ_0007540 | -1.452 | 1.50E-02 | Down-regulated |
| 1516 | hsa_circ_0006459 | -1.453 | 3.80E-02 | Down-regulated |
| 1517 | hsa_circ_0007822 | -1.455 | 2.50E-02 | Down-regulated |
| 1518 | hsa_circ_0002831 | -1.455 | 3.10E-02 | Down-regulated |
| 1519 | hsa_circ_0052154 | -1.456 | 3.50E-02 | Down-regulated |
| 1520 | hsa_circ_0077086 | -1.456 | 2.50E-02 | Down-regulated |
| 1521 | hsa_circ_0061853 | -1.456 | 2.70E-02 | Down-regulated |
| 1522 | hsa_circ_0035643 | -1.457 | 3.50E-02 | Down-regulated |
| 1523 | hsa_circ_0009550 | -1.457 | 4.40E-02 | Down-regulated |
| 1524 | hsa_circ_0004293 | -1.458 | 2.70E-02 | Down-regulated |
| 1525 | hsa_circ_0033480 | -1.458 | 4.30E-02 | Down-regulated |
| 1526 | hsa_circ_0084501 | -1.459 | 2.10E-02 | Down-regulated |
| 1527 | hsa_circ_0074206 | -1.460 | 1.90E-02 | Down-regulated |
| 1528 | hsa_circ_0070245 | -1.460 | 2.90E-02 | Down-regulated |
| 1529 | hsa_circ_0084171 | -1.460 | 3.80E-02 | Down-regulated |
| 1530 | hsa_circ_0066649 | -1.460 | 1.70E-02 | Down-regulated |
| 1531 | hsa_circ_0001406 | -1.460 | 1.00E-02 | Down-regulated |
| 1532 | hsa_circ_0070400 | -1.460 | 4.00E-02 | Down-regulated |
| 1533 | hsa_circ_0078488 | -1.460 | 4.80E-02 | Down-regulated |
| 1534 | hsa_circ_0001616 | -1.460 | 1.00E-02 | Down-regulated |
| 1535 | hsa_circ_0033148 | -1.461 | 2.90E-02 | Down-regulated |
| 1536 | hsa_circ_0002577 | -1.461 | 3.20E-02 | Down-regulated |
| 1537 | hsa_circ_0020275 | -1.461 | 5.00E-03 | Down-regulated |
| 1538 | hsa_circ_0008601 | -1.462 | 1.90E-02 | Down-regulated |
| 1539 | hsa_circ_0054740 | -1.462 | 2.10E-02 | Down-regulated |
| 1540 | hsa_circ_0069725 | -1.462 | 1.30E-02 | Down-regulated |
| 1541 | hsa_circ_0091104 | -1.463 | 3.00E-03 | Down-regulated |
| 1542 | hsa_circ_0069724 | -1.463 | 1.30E-02 | Down-regulated |
| 1543 | hsa_circ_0063147 | -1.464 | 4.50E-02 | Down-regulated |
| 1544 | hsa_circ_0038975 | -1.464 | 2.60E-02 | Down-regulated |
| 1545 | hsa_circ_0055387 | -1.465 | 4.90E-02 | Down-regulated |
| 1546 | hsa_circ_0087493 | -1.465 | 1.80E-02 | Down-regulated |
| 1547 | hsa_circ_0089169 | -1.465 | 3.60E-02 | Down-regulated |
| 1548 | hsa_circ_0001069 | -1.466 | 6.00E-03 | Down-regulated |
| 1549 | hsa_circ_0057521 | -1.467 | 2.70E-02 | Down-regulated |
| 1550 | hsa_circ_0001786 | -1.467 | 6.00E-03 | Down-regulated |
| 1551 | hsa_circ_0054104 | -1.467 | 1.70E-02 | Down-regulated |
| 1552 | hsa_circ_0027573 | -1.467 | 7.00E-03 | Down-regulated |
| 1553 | hsa_circ_0021526 | -1.467 | 4.50E-02 | Down-regulated |
| 1554 | hsa_circ_0052522 | -1.468 | 3.40E-02 | Down-regulated |
| 1555 | hsa_circ_0008272 | -1.468 | 1.20E-02 | Down-regulated |
| 1556 | hsa_circ_0008827 | -1.468 | 2.10E-02 | Down-regulated |
| 1557 | hsa_circ_0036399 | -1.470 | 3.10E-02 | Down-regulated |
| 1558 | hsa_circ_0001319 | -1.470 | 2.20E-02 | Down-regulated |
| 1559 | hsa_circ_0031052 | -1.471 | 4.00E-03 | Down-regulated |
| 1560 | hsa_circ_0029634 | -1.471 | 2.70E-02 | Down-regulated |
| 1561 | hsa_circ_0062746 | -1.472 | 2.70E-02 | Down-regulated |
| 1562 | hsa_circ_0007554 | -1.472 | 2.10E-02 | Down-regulated |
| 1563 | hsa_circ_0006382 | -1.472 | 4.30E-02 | Down-regulated |
| 1564 | hsa_circ_0070022 | -1.473 | 1.90E-02 | Down-regulated |
| 1565 | hsa_circ_0070284 | -1.473 | 2.50E-02 | Down-regulated |
| 1566 | hsa_circ_0044136 | -1.473 | 1.00E-02 | Down-regulated |
| 1567 | hsa_circ_0027572 | -1.474 | 3.40E-02 | Down-regulated |
| 1568 | hsa_circ_0007570 | -1.474 | 4.00E-03 | Down-regulated |
| 1569 | hsa_circ_0057234 | -1.474 | 3.00E-03 | Down-regulated |
| 1570 | hsa_circ_0071228 | -1.474 | 9.00E-03 | Down-regulated |
| 1571 | hsa_circ_0004954 | -1.474 | 4.10E-02 | Down-regulated |
| 1572 | hsa_circ_0075511 | -1.475 | 1.40E-02 | Down-regulated |
| 1573 | hsa_circ_0001283 | -1.477 | 1.90E-02 | Down-regulated |
| 1574 | hsa_circ_0087828 | -1.478 | 3.20E-02 | Down-regulated |
| 1575 | hsa_circ_0062390 | -1.479 | 2.10E-02 | Down-regulated |
| 1576 | hsa_circ_0044301 | -1.480 | 3.90E-02 | Down-regulated |
| 1577 | hsa_circ_0012451 | -1.480 | 2.00E-02 | Down-regulated |
| 1578 | hsa_circ_0061575 | -1.480 | 7.00E-03 | Down-regulated |
| 1579 | hsa_circ_0006392 | -1.480 | 2.60E-02 | Down-regulated |
| 1580 | hsa_circ_0001349 | -1.480 | 1.60E-02 | Down-regulated |
| 1581 | hsa_circ_0004872 | -1.481 | 3.30E-02 | Down-regulated |
| 1582 | hsa_circ_0001890 | -1.481 | 2.80E-02 | Down-regulated |
| 1583 | hsa_circ_0058521 | -1.481 | 3.10E-02 | Down-regulated |
| 1584 | hsa_circ_0031720 | -1.481 | 2.20E-02 | Down-regulated |
| 1585 | hsa_circ_0003591 | -1.481 | 2.90E-02 | Down-regulated |
| 1586 | hsa_circ_0003018 | -1.482 | 2.50E-02 | Down-regulated |
| 1587 | hsa_circ_0057929 | -1.482 | 9.00E-03 | Down-regulated |
| 1588 | hsa_circ_0000349 | -1.482 | 1.40E-02 | Down-regulated |
| 1589 | hsa_circ_0016599 | -1.482 | 3.40E-02 | Down-regulated |
| 1590 | hsa_circ_0008546 | -1.483 | 4.50E-02 | Down-regulated |
| 1591 | hsa_circ_0039241 | -1.483 | 2.30E-02 | Down-regulated |
| 1592 | hsa_circ_0030632 | -1.484 | 1.40E-02 | Down-regulated |
| 1593 | hsa_circ_0011167 | -1.484 | 1.70E-02 | Down-regulated |
| 1594 | hsa_circ_0089823 | -1.484 | 8.00E-03 | Down-regulated |
| 1595 | hsa_circ_0038799 | -1.484 | 4.90E-02 | Down-regulated |
| 1596 | hsa_circ_0084677 | -1.485 | 3.10E-02 | Down-regulated |
| 1597 | hsa_circ_0000096 | -1.485 | 2.90E-02 | Down-regulated |
| 1598 | hsa_circ_0003568 | -1.485 | 3.80E-02 | Down-regulated |
| 1599 | hsa_circ_0000827 | -1.486 | 3.20E-02 | Down-regulated |
| 1600 | hsa_circ_0050532 | -1.486 | 1.40E-02 | Down-regulated |
| 1601 | hsa_circ_0007439 | -1.487 | 5.00E-02 | Down-regulated |
| 1602 | hsa_circ_0070857 | -1.487 | 2.70E-02 | Down-regulated |
| 1603 | hsa_circ_0000703 | -1.488 | 3.20E-02 | Down-regulated |
| 1604 | hsa_circ_0041700 | -1.488 | 2.90E-02 | Down-regulated |
| 1605 | hsa_circ_0003183 | -1.488 | 1.90E-02 | Down-regulated |
| 1606 | hsa_circ_0059665 | -1.489 | 1.30E-02 | Down-regulated |
| 1607 | hsa_circ_0067772 | -1.489 | 1.70E-02 | Down-regulated |
| 1608 | hsa_circ_0061395 | -1.489 | 1.90E-02 | Down-regulated |
| 1609 | hsa_circ_0037902 | -1.489 | 1.80E-02 | Down-regulated |
| 1610 | hsa_circ_0000771 | -1.490 | 3.80E-02 | Down-regulated |
| 1611 | hsa_circ_0055054 | -1.491 | 2.00E-02 | Down-regulated |
| 1612 | hsa_circ_0012384 | -1.491 | 2.20E-02 | Down-regulated |
| 1613 | hsa_circ_0008257 | -1.492 | 6.00E-03 | Down-regulated |
| 1614 | hsa_circ_0064690 | -1.492 | 1.60E-02 | Down-regulated |
| 1615 | hsa_circ_0063331 | -1.492 | 4.00E-02 | Down-regulated |
| 1616 | hsa_circ_0040370 | -1.492 | 2.30E-02 | Down-regulated |
| 1617 | hsa_circ_0067240 | -1.494 | 4.50E-02 | Down-regulated |
| 1618 | hsa_circ_0001492 | -1.494 | 1.90E-02 | Down-regulated |
| 1619 | hsa_circ_0016861 | -1.494 | 1.50E-02 | Down-regulated |
| 1620 | hsa_circ_0001613 | -1.494 | 1.50E-02 | Down-regulated |
| 1621 | hsa_circ_0057283 | -1.494 | 2.50E-02 | Down-regulated |
| 1622 | hsa_circ_0035052 | -1.495 | 1.80E-02 | Down-regulated |
| 1623 | hsa_circ_0042451 | -1.495 | 2.40E-02 | Down-regulated |
| 1624 | hsa_circ_0067343 | -1.495 | 1.50E-02 | Down-regulated |
| 1625 | hsa_circ_0055538 | -1.496 | 6.00E-03 | Down-regulated |
| 1626 | hsa_circ_0002863 | -1.496 | 2.10E-02 | Down-regulated |
| 1627 | hsa_circ_0029633 | -1.496 | 2.00E-02 | Down-regulated |
| 1628 | hsa_circ_0006688 | -1.498 | 1.10E-02 | Down-regulated |
| 1629 | hsa_circ_0004134 | -1.498 | 4.80E-02 | Down-regulated |
| 1630 | hsa_circ_0007007 | -1.498 | 2.60E-02 | Down-regulated |
| 1631 | hsa_circ_0061346 | -1.498 | 2.80E-02 | Down-regulated |
| 1632 | hsa_circ_0063257 | -1.498 | 8.00E-03 | Down-regulated |
| 1633 | hsa_circ_0015183 | -1.498 | 1.70E-02 | Down-regulated |
| 1634 | hsa_circ_0052621 | -1.498 | 4.10E-02 | Down-regulated |
| 1635 | hsa_circ_0001007 | -1.499 | 1.60E-02 | Down-regulated |
| 1636 | hsa_circ_0085294 | -1.499 | 4.60E-02 | Down-regulated |
| 1637 | hsa_circ_0053343 | -1.499 | 0.00E+00 | Down-regulated |
| 1638 | hsa_circ_0056234 | -1.500 | 7.00E-03 | Down-regulated |
| 1639 | hsa_circ_0011883 | -1.501 | 3.40E-02 | Down-regulated |
| 1640 | hsa_circ_0077535 | -1.503 | 1.00E-02 | Down-regulated |
| 1641 | hsa_circ_0005633 | -1.503 | 2.20E-02 | Down-regulated |
| 1642 | hsa_circ_0004823 | -1.504 | 4.80E-02 | Down-regulated |
| 1643 | hsa_circ_0000600 | -1.504 | 2.20E-02 | Down-regulated |
| 1644 | hsa_circ_0066595 | -1.505 | 1.00E-02 | Down-regulated |
| 1645 | hsa_circ_0006654 | -1.505 | 3.50E-02 | Down-regulated |
| 1646 | hsa_circ_0009689 | -1.505 | 2.30E-02 | Down-regulated |
| 1647 | hsa_circ_0077032 | -1.506 | 1.00E-03 | Down-regulated |
| 1648 | hsa_circ_0056834 | -1.507 | 2.10E-02 | Down-regulated |
| 1649 | hsa_circ_0014037 | -1.507 | 1.50E-02 | Down-regulated |
| 1650 | hsa_circ_0021578 | -1.507 | 1.50E-02 | Down-regulated |
| 1651 | hsa_circ_0003742 | -1.508 | 2.70E-02 | Down-regulated |
| 1652 | hsa_circ_0007127 | -1.508 | 1.50E-02 | Down-regulated |
| 1653 | hsa_circ_0007539 | -1.509 | 1.60E-02 | Down-regulated |
| 1654 | hsa_circ_0001375 | -1.509 | 2.60E-02 | Down-regulated |
| 1655 | hsa_circ_0008192 | -1.509 | 4.70E-02 | Down-regulated |
| 1656 | hsa_circ_0054339 | -1.510 | 1.70E-02 | Down-regulated |
| 1657 | hsa_circ_0004773 | -1.510 | 3.10E-02 | Down-regulated |
| 1658 | hsa_circ_0015107 | -1.510 | 2.20E-02 | Down-regulated |
| 1659 | hsa_circ_0002165 | -1.510 | 1.70E-02 | Down-regulated |
| 1660 | hsa_circ_0009140 | -1.511 | 2.70E-02 | Down-regulated |
| 1661 | hsa_circ_0014263 | -1.511 | 7.00E-03 | Down-regulated |
| 1662 | hsa_circ_0003307 | -1.511 | 4.30E-02 | Down-regulated |
| 1663 | hsa_circ_0005274 | -1.512 | 7.00E-03 | Down-regulated |
| 1664 | hsa_circ_0075267 | -1.512 | 1.90E-02 | Down-regulated |
| 1665 | hsa_circ_0073580 | -1.512 | 3.60E-02 | Down-regulated |
| 1666 | hsa_circ_0005899 | -1.514 | 3.60E-02 | Down-regulated |
| 1667 | hsa_circ_0053651 | -1.514 | 3.40E-02 | Down-regulated |
| 1668 | hsa_circ_0084493 | -1.515 | 1.10E-02 | Down-regulated |
| 1669 | hsa_circ_0045850 | -1.515 | 3.40E-02 | Down-regulated |
| 1670 | hsa_circ_0053936 | -1.515 | 7.00E-03 | Down-regulated |
| 1671 | hsa_circ_0088284 | -1.516 | 1.60E-02 | Down-regulated |
| 1672 | hsa_circ_0035152 | -1.516 | 3.60E-02 | Down-regulated |
| 1673 | hsa_circ_0043544 | -1.517 | 2.20E-02 | Down-regulated |
| 1674 | hsa_circ_0077953 | -1.519 | 1.30E-02 | Down-regulated |
| 1675 | hsa_circ_0007625 | -1.520 | 1.20E-02 | Down-regulated |
| 1676 | hsa_circ_0061280 | -1.520 | 3.90E-02 | Down-regulated |
| 1677 | hsa_circ_0039337 | -1.521 | 6.00E-03 | Down-regulated |
| 1678 | hsa_circ_0083386 | -1.521 | 3.10E-02 | Down-regulated |
| 1679 | hsa_circ_0082690 | -1.521 | 3.30E-02 | Down-regulated |
| 1680 | hsa_circ_0091468 | -1.522 | 1.50E-02 | Down-regulated |
| 1681 | hsa_circ_0006807 | -1.522 | 4.50E-02 | Down-regulated |
| 1682 | hsa_circ_0036510 | -1.523 | 2.80E-02 | Down-regulated |
| 1683 | hsa_circ_0001146 | -1.523 | 2.80E-02 | Down-regulated |
| 1684 | hsa_circ_0027491 | -1.524 | 4.40E-02 | Down-regulated |
| 1685 | hsa_circ_0011140 | -1.525 | 4.30E-02 | Down-regulated |
| 1686 | hsa_circ_0001317 | -1.526 | 4.70E-02 | Down-regulated |
| 1687 | hsa_circ_0000051 | -1.527 | 1.20E-02 | Down-regulated |
| 1688 | hsa_circ_0000555 | -1.528 | 2.70E-02 | Down-regulated |
| 1689 | hsa_circ_0054205 | -1.529 | 1.60E-02 | Down-regulated |
| 1690 | hsa_circ_0014505 | -1.529 | 1.60E-02 | Down-regulated |
| 1691 | hsa_circ_0054595 | -1.529 | 2.40E-02 | Down-regulated |
| 1692 | hsa_circ_0031627 | -1.529 | 1.30E-02 | Down-regulated |
| 1693 | hsa_circ_0087264 | -1.529 | 3.20E-02 | Down-regulated |
| 1694 | hsa_circ_0070497 | -1.531 | 9.00E-03 | Down-regulated |
| 1695 | hsa_circ_0044839 | -1.531 | 1.50E-02 | Down-regulated |
| 1696 | hsa_circ_0008815 | -1.532 | 1.50E-02 | Down-regulated |
| 1697 | hsa_circ_0008465 | -1.532 | 3.30E-02 | Down-regulated |
| 1698 | hsa_circ_0078085 | -1.533 | 1.70E-02 | Down-regulated |
| 1699 | hsa_circ_0002356 | -1.533 | 1.00E-02 | Down-regulated |
| 1700 | hsa_circ_0002940 | -1.533 | 2.70E-02 | Down-regulated |
| 1701 | hsa_circ_0005664 | -1.534 | 2.60E-02 | Down-regulated |
| 1702 | hsa_circ_0030991 | -1.535 | 1.30E-02 | Down-regulated |
| 1703 | hsa_circ_0073930 | -1.535 | 2.80E-02 | Down-regulated |
| 1704 | hsa_circ_0081807 | -1.536 | 2.40E-02 | Down-regulated |
| 1705 | hsa_circ_0067682 | -1.537 | 1.90E-02 | Down-regulated |
| 1706 | hsa_circ_0026127 | -1.538 | 1.50E-02 | Down-regulated |
| 1707 | hsa_circ_0016266 | -1.538 | 1.50E-02 | Down-regulated |
| 1708 | hsa_circ_0008592 | -1.539 | 4.20E-02 | Down-regulated |
| 1709 | hsa_circ_0065343 | -1.540 | 3.40E-02 | Down-regulated |
| 1710 | hsa_circ_0031775 | -1.540 | 9.00E-03 | Down-regulated |
| 1711 | hsa_circ_0057028 | -1.540 | 2.80E-02 | Down-regulated |
| 1712 | hsa_circ_0084522 | -1.541 | 1.60E-02 | Down-regulated |
| 1713 | hsa_circ_0061739 | -1.541 | 2.20E-02 | Down-regulated |
| 1714 | hsa_circ_0007472 | -1.541 | 2.40E-02 | Down-regulated |
| 1715 | hsa_circ_0002333 | -1.541 | 3.00E-02 | Down-regulated |
| 1716 | hsa_circ_0053752 | -1.542 | 2.10E-02 | Down-regulated |
| 1717 | hsa_circ_0073993 | -1.542 | 1.80E-02 | Down-regulated |
| 1718 | hsa_circ_0008309 | -1.542 | 1.60E-02 | Down-regulated |
| 1719 | hsa_circ_0047128 | -1.543 | 1.30E-02 | Down-regulated |
| 1720 | hsa_circ_0006704 | -1.544 | 3.70E-02 | Down-regulated |
| 1721 | hsa_circ_0002961 | -1.544 | 2.40E-02 | Down-regulated |
| 1722 | hsa_circ_0010932 | -1.544 | 4.60E-02 | Down-regulated |
| 1723 | hsa_circ_0036354 | -1.545 | 2.50E-02 | Down-regulated |
| 1724 | hsa_circ_0092329 | -1.545 | 1.60E-02 | Down-regulated |
| 1725 | hsa_circ_0071794 | -1.545 | 1.70E-02 | Down-regulated |
| 1726 | hsa_circ_0001564 | -1.546 | 1.70E-02 | Down-regulated |
| 1727 | hsa_circ_0000394 | -1.547 | 4.80E-02 | Down-regulated |
| 1728 | hsa_circ_0033493 | -1.548 | 2.30E-02 | Down-regulated |
| 1729 | hsa_circ_0001842 | -1.548 | 3.40E-02 | Down-regulated |
| 1730 | hsa_circ_0001361 | -1.548 | 4.90E-02 | Down-regulated |
| 1731 | hsa_circ_0005796 | -1.549 | 1.40E-02 | Down-regulated |
| 1732 | hsa_circ_0014165 | -1.549 | 1.30E-02 | Down-regulated |
| 1733 | hsa_circ_0002981 | -1.550 | 1.20E-02 | Down-regulated |
| 1734 | hsa_circ_0076704 | -1.550 | 7.00E-03 | Down-regulated |
| 1735 | hsa_circ_0053642 | -1.551 | 2.40E-02 | Down-regulated |
| 1736 | hsa_circ_0057280 | -1.552 | 3.80E-02 | Down-regulated |
| 1737 | hsa_circ_0054908 | -1.552 | 1.80E-02 | Down-regulated |
| 1738 | hsa_circ_0073098 | -1.552 | 2.50E-02 | Down-regulated |
| 1739 | hsa_circ_0035386 | -1.553 | 1.60E-02 | Down-regulated |
| 1740 | hsa_circ_0055116 | -1.554 | 1.40E-02 | Down-regulated |
| 1741 | hsa_circ_0012560 | -1.555 | 1.80E-02 | Down-regulated |
| 1742 | hsa_circ_0002642 | -1.556 | 2.80E-02 | Down-regulated |
| 1743 | hsa_circ_0000798 | -1.557 | 3.20E-02 | Down-regulated |
| 1744 | hsa_circ_0029961 | -1.557 | 2.20E-02 | Down-regulated |
| 1745 | hsa_circ_0005255 | -1.557 | 4.50E-02 | Down-regulated |
| 1746 | hsa_circ_0087400 | -1.557 | 9.00E-03 | Down-regulated |
| 1747 | hsa_circ_0077108 | -1.558 | 1.50E-02 | Down-regulated |
| 1748 | hsa_circ_0025696 | -1.558 | 1.00E-02 | Down-regulated |
| 1749 | hsa_circ_0004317 | -1.559 | 1.50E-02 | Down-regulated |
| 1750 | hsa_circ_0056128 | -1.560 | 9.00E-03 | Down-regulated |
| 1751 | hsa_circ_0001807 | -1.561 | 3.00E-02 | Down-regulated |
| 1752 | hsa_circ_0007292 | -1.562 | 2.20E-02 | Down-regulated |
| 1753 | hsa_circ_0047559 | -1.564 | 1.00E-02 | Down-regulated |
| 1754 | hsa_circ_0031584 | -1.565 | 1.40E-02 | Down-regulated |
| 1755 | hsa_circ_0001165 | -1.566 | 3.80E-02 | Down-regulated |
| 1756 | hsa_circ_0047401 | -1.566 | 1.40E-02 | Down-regulated |
| 1757 | hsa_circ_0000745 | -1.566 | 2.10E-02 | Down-regulated |
| 1758 | hsa_circ_0003643 | -1.567 | 1.40E-02 | Down-regulated |
| 1759 | hsa_circ_0067967 | -1.567 | 3.90E-02 | Down-regulated |
| 1760 | hsa_circ_0044623 | -1.567 | 1.90E-02 | Down-regulated |
| 1761 | hsa_circ_0059062 | -1.567 | 1.80E-02 | Down-regulated |
| 1762 | hsa_circ_0031286 | -1.567 | 3.30E-02 | Down-regulated |
| 1763 | hsa_circ_0008839 | -1.567 | 1.40E-02 | Down-regulated |
| 1764 | hsa_circ_0008336 | -1.568 | 1.60E-02 | Down-regulated |
| 1765 | hsa_circ_0002484 | -1.568 | 2.60E-02 | Down-regulated |
| 1766 | hsa_circ_0068767 | -1.569 | 6.00E-03 | Down-regulated |
| 1767 | hsa_circ_0085139 | -1.569 | 1.90E-02 | Down-regulated |
| 1768 | hsa_circ_0060491 | -1.569 | 3.60E-02 | Down-regulated |
| 1769 | hsa_circ_0023110 | -1.570 | 3.10E-02 | Down-regulated |
| 1770 | hsa_circ_0015381 | -1.570 | 3.50E-02 | Down-regulated |
| 1771 | hsa_circ_0064175 | -1.571 | 1.30E-02 | Down-regulated |
| 1772 | hsa_circ_0006794 | -1.571 | 1.30E-02 | Down-regulated |
| 1773 | hsa_circ_0042190 | -1.573 | 1.90E-02 | Down-regulated |
| 1774 | hsa_circ_0001090 | -1.573 | 1.70E-02 | Down-regulated |
| 1775 | hsa_circ_0005514 | -1.574 | 1.80E-02 | Down-regulated |
| 1776 | hsa_circ_0087557 | -1.575 | 3.00E-02 | Down-regulated |
| 1777 | hsa_circ_0086637 | -1.576 | 2.00E-02 | Down-regulated |
| 1778 | hsa_circ_0076177 | -1.576 | 4.40E-02 | Down-regulated |
| 1779 | hsa_circ_0005031 | -1.576 | 5.00E-03 | Down-regulated |
| 1780 | hsa_circ_0008757 | -1.578 | 2.60E-02 | Down-regulated |
| 1781 | hsa_circ_0057289 | -1.578 | 4.00E-02 | Down-regulated |
| 1782 | hsa_circ_0000256 | -1.579 | 3.40E-02 | Down-regulated |
| 1783 | hsa_circ_0073755 | -1.579 | 1.40E-02 | Down-regulated |
| 1784 | hsa_circ_0064364 | -1.579 | 2.10E-02 | Down-regulated |
| 1785 | hsa_circ_0007967 | -1.580 | 6.00E-03 | Down-regulated |
| 1786 | hsa_circ_0078244 | -1.581 | 1.30E-02 | Down-regulated |
| 1787 | hsa_circ_0010020 | -1.582 | 4.50E-02 | Down-regulated |
| 1788 | hsa_circ_0058123 | -1.582 | 8.00E-03 | Down-regulated |
| 1789 | hsa_circ_0003381 | -1.583 | 3.20E-02 | Down-regulated |
| 1790 | hsa_circ_0000079 | -1.583 | 1.30E-02 | Down-regulated |
| 1791 | hsa_circ_0002216 | -1.584 | 4.70E-02 | Down-regulated |
| 1792 | hsa_circ_0057463 | -1.585 | 1.60E-02 | Down-regulated |
| 1793 | hsa_circ_0001504 | -1.586 | 5.00E-03 | Down-regulated |
| 1794 | hsa_circ_0002448 | -1.586 | 2.10E-02 | Down-regulated |
| 1795 | hsa_circ_0077122 | -1.586 | 1.60E-02 | Down-regulated |
| 1796 | hsa_circ_0005952 | -1.586 | 1.40E-02 | Down-regulated |
| 1797 | hsa_circ_0022029 | -1.589 | 1.10E-02 | Down-regulated |
| 1798 | hsa_circ_0069236 | -1.591 | 1.00E-02 | Down-regulated |
| 1799 | hsa_circ_0058850 | -1.591 | 1.50E-02 | Down-regulated |
| 1800 | hsa_circ_0086443 | -1.591 | 7.00E-03 | Down-regulated |
| 1801 | hsa_circ_0023977 | -1.592 | 1.40E-02 | Down-regulated |
| 1802 | hsa_circ_0017549 | -1.592 | 4.00E-03 | Down-regulated |
| 1803 | hsa_circ_0083964 | -1.592 | 2.10E-02 | Down-regulated |
| 1804 | hsa_circ_0015264 | -1.592 | 1.00E-02 | Down-regulated |
| 1805 | hsa_circ_0003410 | -1.593 | 1.90E-02 | Down-regulated |
| 1806 | hsa_circ_0023936 | -1.593 | 1.60E-02 | Down-regulated |
| 1807 | hsa_circ_0081813 | -1.593 | 2.00E-02 | Down-regulated |
| 1808 | hsa_circ_0001394 | -1.593 | 2.50E-02 | Down-regulated |
| 1809 | hsa_circ_0087159 | -1.595 | 1.60E-02 | Down-regulated |
| 1810 | hsa_circ_0008906 | -1.595 | 3.40E-02 | Down-regulated |
| 1811 | hsa_circ_0011536 | -1.596 | 1.00E-02 | Down-regulated |
| 1812 | hsa_circ_0010440 | -1.596 | 3.60E-02 | Down-regulated |
| 1813 | hsa_circ_0090626 | -1.596 | 2.50E-02 | Down-regulated |
| 1814 | hsa_circ_0068462 | -1.596 | 1.00E-02 | Down-regulated |
| 1815 | hsa_circ_0000169 | -1.597 | 1.70E-02 | Down-regulated |
| 1816 | hsa_circ_0077929 | -1.599 | 3.50E-02 | Down-regulated |
| 1817 | hsa_circ_0087677 | -1.600 | 2.20E-02 | Down-regulated |
| 1818 | hsa_circ_0045404 | -1.600 | 4.50E-02 | Down-regulated |
| 1819 | hsa_circ_0034384 | -1.601 | 1.60E-02 | Down-regulated |
| 1820 | hsa_circ_0011159 | -1.601 | 1.10E-02 | Down-regulated |
| 1821 | hsa_circ_0043931 | -1.601 | 1.20E-02 | Down-regulated |
| 1822 | hsa_circ_0055415 | -1.601 | 1.90E-02 | Down-regulated |
| 1823 | hsa_circ_0024018 | -1.602 | 1.70E-02 | Down-regulated |
| 1824 | hsa_circ_0002303 | -1.603 | 2.50E-02 | Down-regulated |
| 1825 | hsa_circ_0008945 | -1.603 | 1.60E-02 | Down-regulated |
| 1826 | hsa_circ_0041362 | -1.604 | 1.40E-02 | Down-regulated |
| 1827 | hsa_circ_0060158 | -1.604 | 2.20E-02 | Down-regulated |
| 1828 | hsa_circ_0053829 | -1.604 | 2.70E-02 | Down-regulated |
| 1829 | hsa_circ_0084172 | -1.605 | 1.70E-02 | Down-regulated |
| 1830 | hsa_circ_0047700 | -1.605 | 1.60E-02 | Down-regulated |
| 1831 | hsa_circ_0026498 | -1.606 | 1.20E-02 | Down-regulated |
| 1832 | hsa_circ_0070581 | -1.606 | 8.00E-03 | Down-regulated |
| 1833 | hsa_circ_0004428 | -1.606 | 1.50E-02 | Down-regulated |
| 1834 | hsa_circ_0004844 | -1.606 | 7.00E-03 | Down-regulated |
| 1835 | hsa_circ_0071552 | -1.607 | 4.30E-02 | Down-regulated |
| 1836 | hsa_circ_0004152 | -1.607 | 4.50E-02 | Down-regulated |
| 1837 | hsa_circ_0069869 | -1.607 | 1.20E-02 | Down-regulated |
| 1838 | hsa_circ_0028241 | -1.607 | 1.50E-02 | Down-regulated |
| 1839 | hsa_circ_0044287 | -1.609 | 1.50E-02 | Down-regulated |
| 1840 | hsa_circ_0040705 | -1.610 | 6.00E-03 | Down-regulated |
| 1841 | hsa_circ_0067361 | -1.610 | 2.00E-03 | Down-regulated |
| 1842 | hsa_circ_0036599 | -1.610 | 2.20E-02 | Down-regulated |
| 1843 | hsa_circ_0001409 | -1.611 | 1.60E-02 | Down-regulated |
| 1844 | hsa_circ_0002301 | -1.612 | 3.60E-02 | Down-regulated |
| 1845 | hsa_circ_0000424 | -1.612 | 1.10E-02 | Down-regulated |
| 1846 | hsa_circ_0002077 | -1.612 | 2.30E-02 | Down-regulated |
| 1847 | hsa_circ_0037630 | -1.612 | 3.00E-02 | Down-regulated |
| 1848 | hsa_circ_0084444 | -1.613 | 1.90E-02 | Down-regulated |
| 1849 | hsa_circ_0007335 | -1.613 | 3.20E-02 | Down-regulated |
| 1850 | hsa_circ_0039757 | -1.613 | 1.70E-02 | Down-regulated |
| 1851 | hsa_circ_0073932 | -1.614 | 2.30E-02 | Down-regulated |
| 1852 | hsa_circ_0002102 | -1.614 | 3.50E-02 | Down-regulated |
| 1853 | hsa_circ_0065308 | -1.615 | 4.00E-02 | Down-regulated |
| 1854 | hsa_circ_0088390 | -1.616 | 4.20E-02 | Down-regulated |
| 1855 | hsa_circ_0091449 | -1.617 | 1.50E-02 | Down-regulated |
| 1856 | hsa_circ_0072756 | -1.618 | 3.10E-02 | Down-regulated |
| 1857 | hsa_circ_0030670 | -1.619 | 9.00E-03 | Down-regulated |
| 1858 | hsa_circ_0013451 | -1.620 | 2.30E-02 | Down-regulated |
| 1859 | hsa_circ_0005264 | -1.620 | 3.40E-02 | Down-regulated |
| 1860 | hsa_circ_0037896 | -1.620 | 2.10E-02 | Down-regulated |
| 1861 | hsa_circ_0056539 | -1.621 | 3.00E-02 | Down-regulated |
| 1862 | hsa_circ_0009349 | -1.621 | 2.20E-02 | Down-regulated |
| 1863 | hsa_circ_0018100 | -1.622 | 1.50E-02 | Down-regulated |
| 1864 | hsa_circ_0003632 | -1.622 | 1.10E-02 | Down-regulated |
| 1865 | hsa_circ_0006536 | -1.622 | 2.70E-02 | Down-regulated |
| 1866 | hsa_circ_0002602 | -1.622 | 3.90E-02 | Down-regulated |
| 1867 | hsa_circ_0060744 | -1.622 | 1.10E-02 | Down-regulated |
| 1868 | hsa_circ_0047022 | -1.623 | 2.20E-02 | Down-regulated |
| 1869 | hsa_circ_0070941 | -1.624 | 2.50E-02 | Down-regulated |
| 1870 | hsa_circ_0006230 | -1.624 | 1.80E-02 | Down-regulated |
| 1871 | hsa_circ_0000702 | -1.624 | 2.30E-02 | Down-regulated |
| 1872 | hsa_circ_0002649 | -1.624 | 2.40E-02 | Down-regulated |
| 1873 | hsa_circ_0006385 | -1.625 | 1.90E-02 | Down-regulated |
| 1874 | hsa_circ_0005874 | -1.625 | 3.10E-02 | Down-regulated |
| 1875 | hsa_circ_0024231 | -1.625 | 1.30E-02 | Down-regulated |
| 1876 | hsa_circ_0065299 | -1.628 | 1.00E-02 | Down-regulated |
| 1877 | hsa_circ_0054665 | -1.631 | 1.90E-02 | Down-regulated |
| 1878 | hsa_circ_0020407 | -1.631 | 1.80E-02 | Down-regulated |
| 1879 | hsa_circ_0027691 | -1.632 | 6.00E-03 | Down-regulated |
| 1880 | hsa_circ_0072084 | -1.632 | 6.00E-03 | Down-regulated |
| 1881 | hsa_circ_0083866 | -1.632 | 3.00E-02 | Down-regulated |
| 1882 | hsa_circ_0074270 | -1.633 | 2.00E-02 | Down-regulated |
| 1883 | hsa_circ_0019495 | -1.633 | 1.00E-02 | Down-regulated |
| 1884 | hsa_circ_0003205 | -1.634 | 4.80E-02 | Down-regulated |
| 1885 | hsa_circ_0021199 | -1.635 | 1.70E-02 | Down-regulated |
| 1886 | hsa_circ_0017562 | -1.635 | 6.00E-03 | Down-regulated |
| 1887 | hsa_circ_0001423 | -1.635 | 3.60E-02 | Down-regulated |
| 1888 | hsa_circ_0008523 | -1.636 | 3.00E-02 | Down-regulated |
| 1889 | hsa_circ_0001403 | -1.636 | 1.00E-02 | Down-regulated |
| 1890 | hsa_circ_0031482 | -1.636 | 3.40E-02 | Down-regulated |
| 1891 | hsa_circ_0019841 | -1.637 | 3.00E-02 | Down-regulated |
| 1892 | hsa_circ_0054493 | -1.637 | 1.00E-02 | Down-regulated |
| 1893 | hsa_circ_0005917 | -1.637 | 3.30E-02 | Down-regulated |
| 1894 | hsa_circ_0007308 | -1.638 | 3.70E-02 | Down-regulated |
| 1895 | hsa_circ_0002473 | -1.638 | 3.20E-02 | Down-regulated |
| 1896 | hsa_circ_0032135 | -1.638 | 2.40E-02 | Down-regulated |
| 1897 | hsa_circ_0043317 | -1.639 | 2.40E-02 | Down-regulated |
| 1898 | hsa_circ_0024190 | -1.640 | 9.00E-03 | Down-regulated |
| 1899 | hsa_circ_0072714 | -1.641 | 1.80E-02 | Down-regulated |
| 1900 | hsa_circ_0025988 | -1.641 | 3.80E-02 | Down-regulated |
| 1901 | hsa_circ_0070910 | -1.642 | 1.10E-02 | Down-regulated |
| 1902 | hsa_circ_0016648 | -1.644 | 2.00E-02 | Down-regulated |
| 1903 | hsa_circ_0029941 | -1.644 | 1.00E-02 | Down-regulated |
| 1904 | hsa_circ_0004470 | -1.645 | 3.50E-02 | Down-regulated |
| 1905 | hsa_circ_0039663 | -1.645 | 2.40E-02 | Down-regulated |
| 1906 | hsa_circ_0053063 | -1.646 | 1.30E-02 | Down-regulated |
| 1907 | hsa_circ_0012587 | -1.646 | 9.00E-03 | Down-regulated |
| 1908 | hsa_circ_0057072 | -1.646 | 3.60E-02 | Down-regulated |
| 1909 | hsa_circ_0065249 | -1.647 | 2.80E-02 | Down-regulated |
| 1910 | hsa_circ_0002861 | -1.647 | 2.10E-02 | Down-regulated |
| 1911 | hsa_circ_0020005 | -1.647 | 9.00E-03 | Down-regulated |
| 1912 | hsa_circ_0063752 | -1.647 | 1.60E-02 | Down-regulated |
| 1913 | hsa_circ_0057341 | -1.647 | 9.00E-03 | Down-regulated |
| 1914 | hsa_circ_0063756 | -1.648 | 2.40E-02 | Down-regulated |
| 1915 | hsa_circ_0019058 | -1.648 | 8.00E-03 | Down-regulated |
| 1916 | hsa_circ_0002590 | -1.649 | 2.30E-02 | Down-regulated |
| 1917 | hsa_circ_0087265 | -1.650 | 2.80E-02 | Down-regulated |
| 1918 | hsa_circ_0059705 | -1.652 | 1.60E-02 | Down-regulated |
| 1919 | hsa_circ_0080980 | -1.652 | 6.00E-03 | Down-regulated |
| 1920 | hsa_circ_0005322 | -1.652 | 2.50E-02 | Down-regulated |
| 1921 | hsa_circ_0005432 | -1.653 | 7.00E-03 | Down-regulated |
| 1922 | hsa_circ_0003451 | -1.653 | 1.20E-02 | Down-regulated |
| 1923 | hsa_circ_0018203 | -1.656 | 1.30E-02 | Down-regulated |
| 1924 | hsa_circ_0047418 | -1.657 | 6.00E-03 | Down-regulated |
| 1925 | hsa_circ_0022201 | -1.657 | 2.20E-02 | Down-regulated |
| 1926 | hsa_circ_0001943 | -1.657 | 7.00E-03 | Down-regulated |
| 1927 | hsa_circ_0054626 | -1.658 | 2.40E-02 | Down-regulated |
| 1928 | hsa_circ_0060377 | -1.658 | 2.90E-02 | Down-regulated |
| 1929 | hsa_circ_0086622 | -1.659 | 1.00E-02 | Down-regulated |
| 1930 | hsa_circ_0027733 | -1.659 | 6.00E-03 | Down-regulated |
| 1931 | hsa_circ_0004672 | -1.660 | 1.80E-02 | Down-regulated |
| 1932 | hsa_circ_0003280 | -1.661 | 2.80E-02 | Down-regulated |
| 1933 | hsa_circ_0007820 | -1.662 | 2.20E-02 | Down-regulated |
| 1934 | hsa_circ_0056341 | -1.663 | 1.90E-02 | Down-regulated |
| 1935 | hsa_circ_0059456 | -1.664 | 3.60E-02 | Down-regulated |
| 1936 | hsa_circ_0000426 | -1.664 | 7.00E-03 | Down-regulated |
| 1937 | hsa_circ_0079265 | -1.665 | 4.60E-02 | Down-regulated |
| 1938 | hsa_circ_0000345 | -1.665 | 1.10E-02 | Down-regulated |
| 1939 | hsa_circ_0024175 | -1.666 | 1.70E-02 | Down-regulated |
| 1940 | hsa_circ_0007889 | -1.666 | 1.00E-02 | Down-regulated |
| 1941 | hsa_circ_0001379 | -1.666 | 2.00E-02 | Down-regulated |
| 1942 | hsa_circ_0024790 | -1.669 | 7.00E-03 | Down-regulated |
| 1943 | hsa_circ_0000078 | -1.669 | 5.00E-03 | Down-regulated |
| 1944 | hsa_circ_0039934 | -1.669 | 1.00E-02 | Down-regulated |
| 1945 | hsa_circ_0040749 | -1.670 | 1.90E-02 | Down-regulated |
| 1946 | hsa_circ_0066859 | -1.670 | 1.40E-02 | Down-regulated |
| 1947 | hsa_circ_0027427 | -1.672 | 4.80E-02 | Down-regulated |
| 1948 | hsa_circ_0000133 | -1.672 | 1.60E-02 | Down-regulated |
| 1949 | hsa_circ_0004976 | -1.673 | 2.60E-02 | Down-regulated |
| 1950 | hsa_circ_0037377 | -1.673 | 1.70E-02 | Down-regulated |
| 1951 | hsa_circ_0077658 | -1.674 | 8.00E-03 | Down-regulated |
| 1952 | hsa_circ_0025302 | -1.674 | 3.00E-03 | Down-regulated |
| 1953 | hsa_circ_0002120 | -1.675 | 1.90E-02 | Down-regulated |
| 1954 | hsa_circ_0024169 | -1.676 | 7.00E-03 | Down-regulated |
| 1955 | hsa_circ_0002978 | -1.676 | 2.30E-02 | Down-regulated |
| 1956 | hsa_circ_0075161 | -1.678 | 1.90E-02 | Down-regulated |
| 1957 | hsa_circ_0016662 | -1.678 | 2.60E-02 | Down-regulated |
| 1958 | hsa_circ_0000740 | -1.678 | 4.40E-02 | Down-regulated |
| 1959 | hsa_circ_0042986 | -1.680 | 7.00E-03 | Down-regulated |
| 1960 | hsa_circ_0000116 | -1.680 | 5.00E-02 | Down-regulated |
| 1961 | hsa_circ_0007938 | -1.680 | 1.00E-02 | Down-regulated |
| 1962 | hsa_circ_0004163 | -1.681 | 1.20E-02 | Down-regulated |
| 1963 | hsa_circ_0055629 | -1.683 | 2.10E-02 | Down-regulated |
| 1964 | hsa_circ_0025278 | -1.683 | 3.00E-02 | Down-regulated |
| 1965 | hsa_circ_0064986 | -1.684 | 2.20E-02 | Down-regulated |
| 1966 | hsa_circ_0008640 | -1.684 | 1.70E-02 | Down-regulated |
| 1967 | hsa_circ_0046656 | -1.685 | 4.30E-02 | Down-regulated |
| 1968 | hsa_circ_0057261 | -1.685 | 9.00E-03 | Down-regulated |
| 1969 | hsa_circ_0002847 | -1.685 | 2.20E-02 | Down-regulated |
| 1970 | hsa_circ_0006771 | -1.687 | 2.90E-02 | Down-regulated |
| 1971 | hsa_circ_0060762 | -1.687 | 2.10E-02 | Down-regulated |
| 1972 | hsa_circ_0030051 | -1.688 | 8.00E-03 | Down-regulated |
| 1973 | hsa_circ_0020175 | -1.689 | 4.00E-03 | Down-regulated |
| 1974 | hsa_circ_0082096 | -1.689 | 2.20E-02 | Down-regulated |
| 1975 | hsa_circ_0007053 | -1.689 | 3.30E-02 | Down-regulated |
| 1976 | hsa_circ_0006978 | -1.690 | 2.10E-02 | Down-regulated |
| 1977 | hsa_circ_0027766 | -1.690 | 8.00E-03 | Down-regulated |
| 1978 | hsa_circ_0074849 | -1.691 | 9.00E-03 | Down-regulated |
| 1979 | hsa_circ_0025513 | -1.691 | 2.30E-02 | Down-regulated |
| 1980 | hsa_circ_0003226 | -1.693 | 5.00E-03 | Down-regulated |
| 1981 | hsa_circ_0026968 | -1.693 | 2.00E-03 | Down-regulated |
| 1982 | hsa_circ_0005797 | -1.694 | 4.10E-02 | Down-regulated |
| 1983 | hsa_circ_0074137 | -1.694 | 4.00E-02 | Down-regulated |
| 1984 | hsa_circ_0011168 | -1.694 | 2.30E-02 | Down-regulated |
| 1985 | hsa_circ_0001630 | -1.694 | 1.80E-02 | Down-regulated |
| 1986 | hsa_circ_0047481 | -1.695 | 7.00E-03 | Down-regulated |
| 1987 | hsa_circ_0003831 | -1.695 | 1.30E-02 | Down-regulated |
| 1988 | hsa_circ_0000120 | -1.696 | 3.90E-02 | Down-regulated |
| 1989 | hsa_circ_0012172 | -1.696 | 3.60E-02 | Down-regulated |
| 1990 | hsa_circ_0002092 | -1.697 | 2.40E-02 | Down-regulated |
| 1991 | hsa_circ_0023566 | -1.699 | 6.00E-03 | Down-regulated |
| 1992 | hsa_circ_0000420 | -1.700 | 3.20E-02 | Down-regulated |
| 1993 | hsa_circ_0002008 | -1.701 | 3.00E-02 | Down-regulated |
| 1994 | hsa_circ_0058213 | -1.701 | 2.90E-02 | Down-regulated |
| 1995 | hsa_circ_0034160 | -1.701 | 7.00E-03 | Down-regulated |
| 1996 | hsa_circ_0028864 | -1.702 | 1.00E-02 | Down-regulated |
| 1997 | hsa_circ_0057319 | -1.702 | 6.00E-03 | Down-regulated |
| 1998 | hsa_circ_0001698 | -1.703 | 2.40E-02 | Down-regulated |
| 1999 | hsa_circ_0001802 | -1.704 | 1.50E-02 | Down-regulated |
| 2000 | hsa_circ_0061577 | -1.704 | 1.00E-02 | Down-regulated |
| 2001 | hsa_circ_0011542 | -1.704 | 1.70E-02 | Down-regulated |
| 2002 | hsa_circ_0027934 | -1.705 | 1.50E-02 | Down-regulated |
| 2003 | hsa_circ_0006248 | -1.705 | 2.40E-02 | Down-regulated |
| 2004 | hsa_circ_0019181 | -1.705 | 1.20E-02 | Down-regulated |
| 2005 | hsa_circ_0006990 | -1.706 | 2.80E-02 | Down-regulated |
| 2006 | hsa_circ_0078367 | -1.707 | 1.90E-02 | Down-regulated |
| 2007 | hsa_circ_0080242 | -1.708 | 8.00E-03 | Down-regulated |
| 2008 | hsa_circ_0024139 | -1.709 | 1.40E-02 | Down-regulated |
| 2009 | hsa_circ_0072099 | -1.712 | 1.00E-02 | Down-regulated |
| 2010 | hsa_circ_0008201 | -1.713 | 3.00E-02 | Down-regulated |
| 2011 | hsa_circ_0005304 | -1.714 | 1.30E-02 | Down-regulated |
| 2012 | hsa_circ_0062936 | -1.715 | 2.50E-02 | Down-regulated |
| 2013 | hsa_circ_0024067 | -1.715 | 6.00E-03 | Down-regulated |
| 2014 | hsa_circ_0027776 | -1.715 | 8.00E-03 | Down-regulated |
| 2015 | hsa_circ_0018419 | -1.716 | 1.00E-02 | Down-regulated |
| 2016 | hsa_circ_0034293 | -1.718 | 2.70E-02 | Down-regulated |
| 2017 | hsa_circ_0025693 | -1.718 | 1.60E-02 | Down-regulated |
| 2018 | hsa_circ_0020613 | -1.720 | 1.50E-02 | Down-regulated |
| 2019 | hsa_circ_0064080 | -1.721 | 1.70E-02 | Down-regulated |
| 2020 | hsa_circ_0013048 | -1.722 | 4.00E-03 | Down-regulated |
| 2021 | hsa_circ_0031776 | -1.722 | 8.00E-03 | Down-regulated |
| 2022 | hsa_circ_0070635 | -1.722 | 2.90E-02 | Down-regulated |
| 2023 | hsa_circ_0024501 | -1.723 | 2.80E-02 | Down-regulated |
| 2024 | hsa_circ_0007945 | -1.723 | 1.80E-02 | Down-regulated |
| 2025 | hsa_circ_0044647 | -1.724 | 2.50E-02 | Down-regulated |
| 2026 | hsa_circ_0053317 | -1.724 | 6.00E-03 | Down-regulated |
| 2027 | hsa_circ_0001358 | -1.725 | 1.40E-02 | Down-regulated |
| 2028 | hsa_circ_0078563 | -1.726 | 2.10E-02 | Down-regulated |
| 2029 | hsa_circ_0013171 | -1.727 | 3.50E-02 | Down-regulated |
| 2030 | hsa_circ_0072902 | -1.727 | 1.30E-02 | Down-regulated |
| 2031 | hsa_circ_0010470 | -1.728 | 2.50E-02 | Down-regulated |
| 2032 | hsa_circ_0056204 | -1.730 | 1.20E-02 | Down-regulated |
| 2033 | hsa_circ_0005742 | -1.730 | 1.70E-02 | Down-regulated |
| 2034 | hsa_circ_0072473 | -1.731 | 2.50E-02 | Down-regulated |
| 2035 | hsa_circ_0003711 | -1.731 | 2.00E-02 | Down-regulated |
| 2036 | hsa_circ_0058733 | -1.731 | 2.90E-02 | Down-regulated |
| 2037 | hsa_circ_0005898 | -1.732 | 1.80E-02 | Down-regulated |
| 2038 | hsa_circ_0077659 | -1.733 | 1.60E-02 | Down-regulated |
| 2039 | hsa_circ_0041783 | -1.734 | 6.00E-03 | Down-regulated |
| 2040 | hsa_circ_0060596 | -1.734 | 1.00E-02 | Down-regulated |
| 2041 | hsa_circ_0027713 | -1.734 | 5.00E-03 | Down-regulated |
| 2042 | hsa_circ_0030643 | -1.734 | 1.80E-02 | Down-regulated |
| 2043 | hsa_circ_0001276 | -1.735 | 2.20E-02 | Down-regulated |
| 2044 | hsa_circ_0018803 | -1.735 | 1.70E-02 | Down-regulated |
| 2045 | hsa_circ_0004127 | -1.737 | 3.40E-02 | Down-regulated |
| 2046 | hsa_circ_0073492 | -1.737 | 4.30E-02 | Down-regulated |
| 2047 | hsa_circ_0001528 | -1.737 | 1.90E-02 | Down-regulated |
| 2048 | hsa_circ_0082316 | -1.738 | 4.20E-02 | Down-regulated |
| 2049 | hsa_circ_0005801 | -1.739 | 2.20E-02 | Down-regulated |
| 2050 | hsa_circ_0062984 | -1.739 | 2.30E-02 | Down-regulated |
| 2051 | hsa_circ_0065871 | -1.739 | 1.00E-02 | Down-regulated |
| 2052 | hsa_circ_0000425 | -1.740 | 6.00E-03 | Down-regulated |
| 2053 | hsa_circ_0070647 | -1.740 | 1.70E-02 | Down-regulated |
| 2054 | hsa_circ_0053070 | -1.741 | 1.50E-02 | Down-regulated |
| 2055 | hsa_circ_0075796 | -1.743 | 3.00E-03 | Down-regulated |
| 2056 | hsa_circ_0070897 | -1.743 | 1.30E-02 | Down-regulated |
| 2057 | hsa_circ_0019509 | -1.743 | 3.90E-02 | Down-regulated |
| 2058 | hsa_circ_0013255 | -1.743 | 7.00E-03 | Down-regulated |
| 2059 | hsa_circ_0035026 | -1.744 | 3.60E-02 | Down-regulated |
| 2060 | hsa_circ_0000522 | -1.744 | 1.70E-02 | Down-regulated |
| 2061 | hsa_circ_0020814 | -1.744 | 2.40E-02 | Down-regulated |
| 2062 | hsa_circ_0013530 | -1.744 | 3.40E-02 | Down-regulated |
| 2063 | hsa_circ_0006337 | -1.744 | 1.70E-02 | Down-regulated |
| 2064 | hsa_circ_0079330 | -1.745 | 1.50E-02 | Down-regulated |
| 2065 | hsa_circ_0044963 | -1.745 | 1.20E-02 | Down-regulated |
| 2066 | hsa_circ_0041193 | -1.746 | 4.40E-02 | Down-regulated |
| 2067 | hsa_circ_0004283 | -1.747 | 1.30E-02 | Down-regulated |
| 2068 | hsa_circ_0000464 | -1.747 | 3.90E-02 | Down-regulated |
| 2069 | hsa_circ_0000261 | -1.749 | 1.30E-02 | Down-regulated |
| 2070 | hsa_circ_0071616 | -1.750 | 2.50E-02 | Down-regulated |
| 2071 | hsa_circ_0006979 | -1.751 | 2.40E-02 | Down-regulated |
| 2072 | hsa_circ_0000889 | -1.751 | 3.10E-02 | Down-regulated |
| 2073 | hsa_circ_0064835 | -1.752 | 1.00E-02 | Down-regulated |
| 2074 | hsa_circ_0002822 | -1.752 | 1.10E-02 | Down-regulated |
| 2075 | hsa_circ_0086427 | -1.752 | 8.00E-03 | Down-regulated |
| 2076 | hsa_circ_0059426 | -1.753 | 4.00E-03 | Down-regulated |
| 2077 | hsa_circ_0025866 | -1.754 | 2.40E-02 | Down-regulated |
| 2078 | hsa_circ_0002232 | -1.754 | 6.00E-03 | Down-regulated |
| 2079 | hsa_circ_0018909 | -1.754 | 2.40E-02 | Down-regulated |
| 2080 | hsa_circ_0004148 | -1.754 | 2.40E-02 | Down-regulated |
| 2081 | hsa_circ_0059703 | -1.756 | 2.40E-02 | Down-regulated |
| 2082 | hsa_circ_0068702 | -1.756 | 1.00E-02 | Down-regulated |
| 2083 | hsa_circ_0034510 | -1.757 | 7.00E-03 | Down-regulated |
| 2084 | hsa_circ_0080712 | -1.760 | 2.20E-02 | Down-regulated |
| 2085 | hsa_circ_0051238 | -1.763 | 4.10E-02 | Down-regulated |
| 2086 | hsa_circ_0031038 | -1.764 | 1.00E-02 | Down-regulated |
| 2087 | hsa_circ_0072396 | -1.765 | 1.30E-02 | Down-regulated |
| 2088 | hsa_circ_0051246 | -1.765 | 5.00E-03 | Down-regulated |
| 2089 | hsa_circ_0052458 | -1.766 | 3.30E-02 | Down-regulated |
| 2090 | hsa_circ_0008846 | -1.768 | 2.00E-03 | Down-regulated |
| 2091 | hsa_circ_0000670 | -1.768 | 3.20E-02 | Down-regulated |
| 2092 | hsa_circ_0084753 | -1.768 | 3.70E-02 | Down-regulated |
| 2093 | hsa_circ_0042474 | -1.770 | 8.00E-03 | Down-regulated |
| 2094 | hsa_circ_0007970 | -1.770 | 3.00E-02 | Down-regulated |
| 2095 | hsa_circ_0071063 | -1.770 | 3.00E-02 | Down-regulated |
| 2096 | hsa_circ_0017979 | -1.771 | 2.60E-02 | Down-regulated |
| 2097 | hsa_circ_0067938 | -1.772 | 1.20E-02 | Down-regulated |
| 2098 | hsa_circ_0003315 | -1.773 | 3.30E-02 | Down-regulated |
| 2099 | hsa_circ_0004780 | -1.773 | 3.00E-02 | Down-regulated |
| 2100 | hsa_circ_0085144 | -1.773 | 1.60E-02 | Down-regulated |
| 2101 | hsa_circ_0000821 | -1.773 | 1.60E-02 | Down-regulated |
| 2102 | hsa_circ_0010913 | -1.774 | 1.90E-02 | Down-regulated |
| 2103 | hsa_circ_0019068 | -1.775 | 1.00E-02 | Down-regulated |
| 2104 | hsa_circ_0000356 | -1.776 | 1.80E-02 | Down-regulated |
| 2105 | hsa_circ_0081751 | -1.777 | 1.30E-02 | Down-regulated |
| 2106 | hsa_circ_0000241 | -1.777 | 3.10E-02 | Down-regulated |
| 2107 | hsa_circ_0019033 | -1.777 | 2.40E-02 | Down-regulated |
| 2108 | hsa_circ_0023730 | -1.778 | 1.50E-02 | Down-regulated |
| 2109 | hsa_circ_0004437 | -1.781 | 1.70E-02 | Down-regulated |
| 2110 | hsa_circ_0005940 | -1.781 | 3.60E-02 | Down-regulated |
| 2111 | hsa_circ_0024948 | -1.781 | 2.30E-02 | Down-regulated |
| 2112 | hsa_circ_0011228 | -1.783 | 1.40E-02 | Down-regulated |
| 2113 | hsa_circ_0004833 | -1.784 | 1.30E-02 | Down-regulated |
| 2114 | hsa_circ_0001801 | -1.785 | 2.60E-02 | Down-regulated |
| 2115 | hsa_circ_0005771 | -1.786 | 4.70E-02 | Down-regulated |
| 2116 | hsa_circ_0024251 | -1.787 | 4.00E-02 | Down-regulated |
| 2117 | hsa_circ_0075728 | -1.789 | 3.60E-02 | Down-regulated |
| 2118 | hsa_circ_0001522 | -1.790 | 1.00E-02 | Down-regulated |
| 2119 | hsa_circ_0000423 | -1.790 | 2.00E-02 | Down-regulated |
| 2120 | hsa_circ_0001699 | -1.790 | 1.20E-02 | Down-regulated |
| 2121 | hsa_circ_0008327 | -1.790 | 3.40E-02 | Down-regulated |
| 2122 | hsa_circ_0070355 | -1.793 | 1.90E-02 | Down-regulated |
| 2123 | hsa_circ_0008974 | -1.794 | 2.00E-03 | Down-regulated |
| 2124 | hsa_circ_0003898 | -1.795 | 2.30E-02 | Down-regulated |
| 2125 | hsa_circ_0024770 | -1.796 | 1.60E-02 | Down-regulated |
| 2126 | hsa_circ_0071210 | -1.797 | 3.60E-02 | Down-regulated |
| 2127 | hsa_circ_0071022 | -1.797 | 1.40E-02 | Down-regulated |
| 2128 | hsa_circ_0080091 | -1.800 | 4.00E-03 | Down-regulated |
| 2129 | hsa_circ_0005779 | -1.800 | 2.40E-02 | Down-regulated |
| 2130 | hsa_circ_0000558 | -1.800 | 3.10E-02 | Down-regulated |
| 2131 | hsa_circ_0041905 | -1.801 | 1.00E-02 | Down-regulated |
| 2132 | hsa_circ_0004412 | -1.801 | 4.70E-02 | Down-regulated |
| 2133 | hsa_circ_0026512 | -1.801 | 1.30E-02 | Down-regulated |
| 2134 | hsa_circ_0028561 | -1.802 | 2.70E-02 | Down-regulated |
| 2135 | hsa_circ_0035626 | -1.802 | 8.00E-03 | Down-regulated |
| 2136 | hsa_circ_0003081 | -1.803 | 4.80E-02 | Down-regulated |
| 2137 | hsa_circ_0060627 | -1.803 | 3.40E-02 | Down-regulated |
| 2138 | hsa_circ_0006694 | -1.807 | 1.10E-02 | Down-regulated |
| 2139 | hsa_circ_0003214 | -1.807 | 2.00E-03 | Down-regulated |
| 2140 | hsa_circ_0006374 | -1.808 | 3.10E-02 | Down-regulated |
| 2141 | hsa_circ_0061036 | -1.809 | 1.10E-02 | Down-regulated |
| 2142 | hsa_circ_0006806 | -1.810 | 1.60E-02 | Down-regulated |
| 2143 | hsa_circ_0000701 | -1.812 | 2.50E-02 | Down-regulated |
| 2144 | hsa_circ_0021783 | -1.812 | 5.00E-03 | Down-regulated |
| 2145 | hsa_circ_0002500 | -1.812 | 8.00E-03 | Down-regulated |
| 2146 | hsa_circ_0079163 | -1.814 | 1.40E-02 | Down-regulated |
| 2147 | hsa_circ_0043031 | -1.814 | 2.60E-02 | Down-regulated |
| 2148 | hsa_circ_0007364 | -1.814 | 4.80E-02 | Down-regulated |
| 2149 | hsa_circ_0057703 | -1.815 | 3.20E-02 | Down-regulated |
| 2150 | hsa_circ_0056829 | -1.815 | 1.90E-02 | Down-regulated |
| 2151 | hsa_circ_0025201 | -1.815 | 1.60E-02 | Down-regulated |
| 2152 | hsa_circ_0067469 | -1.816 | 1.40E-02 | Down-regulated |
| 2153 | hsa_circ_0070564 | -1.818 | 2.60E-02 | Down-regulated |
| 2154 | hsa_circ_0003529 | -1.820 | 1.40E-02 | Down-regulated |
| 2155 | hsa_circ_0033169 | -1.821 | 2.40E-02 | Down-regulated |
| 2156 | hsa_circ_0065217 | -1.823 | 1.70E-02 | Down-regulated |
| 2157 | hsa_circ_0007503 | -1.824 | 1.30E-02 | Down-regulated |
| 2158 | hsa_circ_0005855 | -1.825 | 4.40E-02 | Down-regulated |
| 2159 | hsa_circ_0000457 | -1.825 | 1.90E-02 | Down-regulated |
| 2160 | hsa_circ_0003695 | -1.827 | 7.00E-03 | Down-regulated |
| 2161 | hsa_circ_0072788 | -1.827 | 8.00E-03 | Down-regulated |
| 2162 | hsa_circ_0036044 | -1.828 | 1.60E-02 | Down-regulated |
| 2163 | hsa_circ_0041195 | -1.830 | 1.60E-02 | Down-regulated |
| 2164 | hsa_circ_0018634 | -1.831 | 1.00E-02 | Down-regulated |
| 2165 | hsa_circ_0000646 | -1.833 | 1.80E-02 | Down-regulated |
| 2166 | hsa_circ_0074441 | -1.836 | 2.40E-02 | Down-regulated |
| 2167 | hsa_circ_0020261 | -1.840 | 1.60E-02 | Down-regulated |
| 2168 | hsa_circ_0008865 | -1.840 | 2.10E-02 | Down-regulated |
| 2169 | hsa_circ_0011722 | -1.841 | 4.90E-02 | Down-regulated |
| 2170 | hsa_circ_0072397 | -1.841 | 1.80E-02 | Down-regulated |
| 2171 | hsa_circ_0037681 | -1.843 | 3.40E-02 | Down-regulated |
| 2172 | hsa_circ_0004119 | -1.843 | 7.00E-03 | Down-regulated |
| 2173 | hsa_circ_0007008 | -1.844 | 3.00E-02 | Down-regulated |
| 2174 | hsa_circ_0005154 | -1.844 | 1.30E-02 | Down-regulated |
| 2175 | hsa_circ_0005078 | -1.844 | 2.10E-02 | Down-regulated |
| 2176 | hsa_circ_0029762 | -1.844 | 2.00E-02 | Down-regulated |
| 2177 | hsa_circ_0005598 | -1.845 | 3.00E-03 | Down-regulated |
| 2178 | hsa_circ_0003517 | -1.846 | 1.40E-02 | Down-regulated |
| 2179 | hsa_circ_0039210 | -1.847 | 1.10E-02 | Down-regulated |
| 2180 | hsa_circ_0001814 | -1.848 | 1.30E-02 | Down-regulated |
| 2181 | hsa_circ_0007878 | -1.849 | 4.20E-02 | Down-regulated |
| 2182 | hsa_circ_0090224 | -1.849 | 4.10E-02 | Down-regulated |
| 2183 | hsa_circ_0080302 | -1.852 | 1.00E-02 | Down-regulated |
| 2184 | hsa_circ_0000826 | -1.853 | 2.20E-02 | Down-regulated |
| 2185 | hsa_circ_0075026 | -1.854 | 2.40E-02 | Down-regulated |
| 2186 | hsa_circ_0058275 | -1.856 | 3.50E-02 | Down-regulated |
| 2187 | hsa_circ_0000533 | -1.859 | 5.00E-03 | Down-regulated |
| 2188 | hsa_circ_0079329 | -1.860 | 1.90E-02 | Down-regulated |
| 2189 | hsa_circ_0013737 | -1.860 | 3.00E-02 | Down-regulated |
| 2190 | hsa_circ_0065551 | -1.860 | 2.00E-02 | Down-regulated |
| 2191 | hsa_circ_0004554 | -1.861 | 2.40E-02 | Down-regulated |
| 2192 | hsa_circ_0001018 | -1.861 | 1.20E-02 | Down-regulated |
| 2193 | hsa_circ_0046843 | -1.861 | 2.70E-02 | Down-regulated |
| 2194 | hsa_circ_0072940 | -1.862 | 3.70E-02 | Down-regulated |
| 2195 | hsa_circ_0082574 | -1.862 | 1.80E-02 | Down-regulated |
| 2196 | hsa_circ_0015663 | -1.864 | 1.50E-02 | Down-regulated |
| 2197 | hsa_circ_0000832 | -1.864 | 2.70E-02 | Down-regulated |
| 2198 | hsa_circ_0010402 | -1.865 | 2.40E-02 | Down-regulated |
| 2199 | hsa_circ_0003304 | -1.865 | 2.80E-02 | Down-regulated |
| 2200 | hsa_circ_0082572 | -1.867 | 1.00E-02 | Down-regulated |
| 2201 | hsa_circ_0001999 | -1.868 | 3.60E-02 | Down-regulated |
| 2202 | hsa_circ_0069498 | -1.868 | 1.90E-02 | Down-regulated |
| 2203 | hsa_circ_0001445 | -1.868 | 1.10E-02 | Down-regulated |
| 2204 | hsa_circ_0001139 | -1.870 | 1.00E-02 | Down-regulated |
| 2205 | hsa_circ_0003916 | -1.871 | 5.00E-03 | Down-regulated |
| 2206 | hsa_circ_0060245 | -1.871 | 1.20E-02 | Down-regulated |
| 2207 | hsa_circ_0002408 | -1.871 | 4.80E-02 | Down-regulated |
| 2208 | hsa_circ_0008915 | -1.874 | 1.30E-02 | Down-regulated |
| 2209 | hsa_circ_0040641 | -1.874 | 5.00E-03 | Down-regulated |
| 2210 | hsa_circ_0007390 | -1.875 | 1.30E-02 | Down-regulated |
| 2211 | hsa_circ_0016498 | -1.877 | 2.40E-02 | Down-regulated |
| 2212 | hsa_circ_0058097 | -1.879 | 9.00E-03 | Down-regulated |
| 2213 | hsa_circ_0001977 | -1.880 | 2.10E-02 | Down-regulated |
| 2214 | hsa_circ_0026976 | -1.881 | 1.10E-02 | Down-regulated |
| 2215 | hsa_circ_0012425 | -1.881 | 2.40E-02 | Down-regulated |
| 2216 | hsa_circ_0047820 | -1.883 | 5.00E-03 | Down-regulated |
| 2217 | hsa_circ_0002819 | -1.884 | 2.90E-02 | Down-regulated |
| 2218 | hsa_circ_0008817 | -1.886 | 3.10E-02 | Down-regulated |
| 2219 | hsa_circ_0008710 | -1.886 | 8.00E-03 | Down-regulated |
| 2220 | hsa_circ_0066629 | -1.886 | 2.60E-02 | Down-regulated |
| 2221 | hsa_circ_0030689 | -1.886 | 2.50E-02 | Down-regulated |
| 2222 | hsa_circ_0006097 | -1.887 | 1.40E-02 | Down-regulated |
| 2223 | hsa_circ_0014362 | -1.888 | 1.90E-02 | Down-regulated |
| 2224 | hsa_circ_0031677 | -1.889 | 4.30E-02 | Down-regulated |
| 2225 | hsa_circ_0002164 | -1.890 | 1.90E-02 | Down-regulated |
| 2226 | hsa_circ_0003580 | -1.894 | 1.80E-02 | Down-regulated |
| 2227 | hsa_circ_0066801 | -1.895 | 8.00E-03 | Down-regulated |
| 2228 | hsa_circ_0042835 | -1.895 | 2.10E-02 | Down-regulated |
| 2229 | hsa_circ_0029229 | -1.895 | 2.00E-02 | Down-regulated |
| 2230 | hsa_circ_0004271 | -1.897 | 1.30E-02 | Down-regulated |
| 2231 | hsa_circ_0019261 | -1.901 | 1.60E-02 | Down-regulated |
| 2232 | hsa_circ_0044251 | -1.901 | 4.70E-02 | Down-regulated |
| 2233 | hsa_circ_0001946 | -1.902 | 3.60E-02 | Down-regulated |
| 2234 | hsa_circ_0004559 | -1.905 | 3.50E-02 | Down-regulated |
| 2235 | hsa_circ_0001414 | -1.906 | 2.00E-02 | Down-regulated |
| 2236 | hsa_circ_0004717 | -1.906 | 1.10E-02 | Down-regulated |
| 2237 | hsa_circ_0067236 | -1.907 | 3.90E-02 | Down-regulated |
| 2238 | hsa_circ_0007209 | -1.907 | 1.90E-02 | Down-regulated |
| 2239 | hsa_circ_0018114 | -1.907 | 1.20E-02 | Down-regulated |
| 2240 | hsa_circ_0055151 | -1.907 | 9.00E-03 | Down-regulated |
| 2241 | hsa_circ_0031647 | -1.909 | 1.30E-02 | Down-regulated |
| 2242 | hsa_circ_0046263 | -1.909 | 1.90E-02 | Down-regulated |
| 2243 | hsa_circ_0008910 | -1.910 | 9.00E-03 | Down-regulated |
| 2244 | hsa_circ_0037866 | -1.910 | 4.80E-02 | Down-regulated |
| 2245 | hsa_circ_0085013 | -1.914 | 5.00E-03 | Down-regulated |
| 2246 | hsa_circ_0003119 | -1.914 | 2.60E-02 | Down-regulated |
| 2247 | hsa_circ_0012491 | -1.916 | 2.00E-02 | Down-regulated |
| 2248 | hsa_circ_0051908 | -1.917 | 4.00E-03 | Down-regulated |
| 2249 | hsa_circ_0074665 | -1.917 | 2.40E-02 | Down-regulated |
| 2250 | hsa_circ_0007911 | -1.918 | 1.40E-02 | Down-regulated |
| 2251 | hsa_circ_0085154 | -1.919 | 1.40E-02 | Down-regulated |
| 2252 | hsa_circ_0002151 | -1.920 | 2.70E-02 | Down-regulated |
| 2253 | hsa_circ_0000799 | -1.921 | 2.40E-02 | Down-regulated |
| 2254 | hsa_circ_0066508 | -1.921 | 1.50E-02 | Down-regulated |
| 2255 | hsa_circ_0005388 | -1.922 | 1.50E-02 | Down-regulated |
| 2256 | hsa_circ_0072492 | -1.923 | 1.60E-02 | Down-regulated |
| 2257 | hsa_circ_0066617 | -1.924 | 1.20E-02 | Down-regulated |
| 2258 | hsa_circ_0073248 | -1.926 | 7.00E-03 | Down-regulated |
| 2259 | hsa_circ_0007652 | -1.927 | 1.50E-02 | Down-regulated |
| 2260 | hsa_circ_0001313 | -1.928 | 9.00E-03 | Down-regulated |
| 2261 | hsa_circ_0023170 | -1.928 | 1.80E-02 | Down-regulated |
| 2262 | hsa_circ_0085323 | -1.929 | 1.70E-02 | Down-regulated |
| 2263 | hsa_circ_0051244 | -1.929 | 7.00E-03 | Down-regulated |
| 2264 | hsa_circ_0007250 | -1.929 | 3.40E-02 | Down-regulated |
| 2265 | hsa_circ_0069486 | -1.929 | 2.30E-02 | Down-regulated |
| 2266 | hsa_circ_0083465 | -1.931 | 2.00E-02 | Down-regulated |
| 2267 | hsa_circ_0001800 | -1.935 | 1.50E-02 | Down-regulated |
| 2268 | hsa_circ_0004808 | -1.935 | 3.40E-02 | Down-regulated |
| 2269 | hsa_circ_0014490 | -1.936 | 1.20E-02 | Down-regulated |
| 2270 | hsa_circ_0020170 | -1.937 | 1.60E-02 | Down-regulated |
| 2271 | hsa_circ_0007643 | -1.937 | 2.80E-02 | Down-regulated |
| 2272 | hsa_circ_0083439 | -1.937 | 3.20E-02 | Down-regulated |
| 2273 | hsa_circ_0001140 | -1.938 | 7.00E-03 | Down-regulated |
| 2274 | hsa_circ_0081224 | -1.940 | 3.40E-02 | Down-regulated |
| 2275 | hsa_circ_0000338 | -1.941 | 1.20E-02 | Down-regulated |
| 2276 | hsa_circ_0044222 | -1.942 | 1.90E-02 | Down-regulated |
| 2277 | hsa_circ_0013158 | -1.943 | 1.00E-02 | Down-regulated |
| 2278 | hsa_circ_0003079 | -1.945 | 2.40E-02 | Down-regulated |
| 2279 | hsa_circ_0038230 | -1.946 | 1.00E-02 | Down-regulated |
| 2280 | hsa_circ_0054264 | -1.947 | 3.00E-02 | Down-regulated |
| 2281 | hsa_circ_0075564 | -1.948 | 2.10E-02 | Down-regulated |
| 2282 | hsa_circ_0004350 | -1.949 | 2.60E-02 | Down-regulated |
| 2283 | hsa_circ_0024187 | -1.951 | 2.60E-02 | Down-regulated |
| 2284 | hsa_circ_0069374 | -1.951 | 1.70E-02 | Down-regulated |
| 2285 | hsa_circ_0010884 | -1.951 | 5.00E-03 | Down-regulated |
| 2286 | hsa_circ_0000226 | -1.953 | 1.80E-02 | Down-regulated |
| 2287 | hsa_circ_0011218 | -1.953 | 1.50E-02 | Down-regulated |
| 2288 | hsa_circ_0006411 | -1.953 | 7.00E-03 | Down-regulated |
| 2289 | hsa_circ_0070299 | -1.955 | 2.70E-02 | Down-regulated |
| 2290 | hsa_circ_0008925 | -1.959 | 1.90E-02 | Down-regulated |
| 2291 | hsa_circ_0046981 | -1.960 | 1.20E-02 | Down-regulated |
| 2292 | hsa_circ_0091074 | -1.960 | 3.30E-02 | Down-regulated |
| 2293 | hsa_circ_0069775 | -1.961 | 2.70E-02 | Down-regulated |
| 2294 | hsa_circ_0020068 | -1.963 | 2.20E-02 | Down-regulated |
| 2295 | hsa_circ_0047021 | -1.965 | 1.50E-02 | Down-regulated |
| 2296 | hsa_circ_0012986 | -1.967 | 1.60E-02 | Down-regulated |
| 2297 | hsa_circ_0006861 | -1.967 | 1.70E-02 | Down-regulated |
| 2298 | hsa_circ_0005761 | -1.969 | 4.90E-02 | Down-regulated |
| 2299 | hsa_circ_0005974 | -1.971 | 3.90E-02 | Down-regulated |
| 2300 | hsa_circ_0077657 | -1.972 | 1.30E-02 | Down-regulated |
| 2301 | hsa_circ_0003639 | -1.972 | 2.90E-02 | Down-regulated |
| 2302 | hsa_circ_0003762 | -1.972 | 1.50E-02 | Down-regulated |
| 2303 | hsa_circ_0087973 | -1.973 | 2.20E-02 | Down-regulated |
| 2304 | hsa_circ_0061372 | -1.974 | 1.40E-02 | Down-regulated |
| 2305 | hsa_circ_0055251 | -1.975 | 7.00E-03 | Down-regulated |
| 2306 | hsa_circ_0009074 | -1.975 | 1.40E-02 | Down-regulated |
| 2307 | hsa_circ_0047827 | -1.978 | 8.00E-03 | Down-regulated |
| 2308 | hsa_circ_0008708 | -1.979 | 1.80E-02 | Down-regulated |
| 2309 | hsa_circ_0072378 | -1.980 | 7.00E-03 | Down-regulated |
| 2310 | hsa_circ_0069519 | -1.980 | 2.40E-02 | Down-regulated |
| 2311 | hsa_circ_0026983 | -1.981 | 1.10E-02 | Down-regulated |
| 2312 | hsa_circ_0085275 | -1.985 | 2.70E-02 | Down-regulated |
| 2313 | hsa_circ_0007581 | -1.987 | 2.40E-02 | Down-regulated |
| 2314 | hsa_circ_0018112 | -1.987 | 2.00E-02 | Down-regulated |
| 2315 | hsa_circ_0003357 | -1.988 | 1.00E-02 | Down-regulated |
| 2316 | hsa_circ_0020028 | -1.990 | 1.70E-02 | Down-regulated |
| 2317 | hsa_circ_0007901 | -1.992 | 1.00E-03 | Down-regulated |
| 2318 | hsa_circ_0086160 | -1.993 | 8.00E-03 | Down-regulated |
| 2319 | hsa_circ_0004610 | -1.995 | 1.20E-02 | Down-regulated |
| 2320 | hsa_circ_0083996 | -1.996 | 2.80E-02 | Down-regulated |
| 2321 | hsa_circ_0054404 | -1.998 | 1.60E-02 | Down-regulated |
| 2322 | hsa_circ_0005671 | -1.998 | 7.00E-03 | Down-regulated |
| 2323 | hsa_circ_0013607 | -1.999 | 2.10E-02 | Down-regulated |
| 2324 | hsa_circ_0051245 | -2.000 | 8.00E-03 | Down-regulated |
| 2325 | hsa_circ_0008107 | -2.000 | 1.30E-02 | Down-regulated |
| 2326 | hsa_circ_0068464 | -2.001 | 1.90E-02 | Down-regulated |
| 2327 | hsa_circ_0001952 | -2.004 | 1.50E-02 | Down-regulated |
| 2328 | hsa_circ_0047962 | -2.004 | 2.90E-02 | Down-regulated |
| 2329 | hsa_circ_0046999 | -2.004 | 1.80E-02 | Down-regulated |
| 2330 | hsa_circ_0064541 | -2.006 | 7.00E-03 | Down-regulated |
| 2331 | hsa_circ_0007841 | -2.006 | 2.50E-02 | Down-regulated |
| 2332 | hsa_circ_0006819 | -2.006 | 1.90E-02 | Down-regulated |
| 2333 | hsa_circ_0027689 | -2.009 | 7.00E-03 | Down-regulated |
| 2334 | hsa_circ_0044436 | -2.011 | 4.00E-02 | Down-regulated |
| 2335 | hsa_circ_0005131 | -2.011 | 2.50E-02 | Down-regulated |
| 2336 | hsa_circ_0007201 | -2.014 | 3.30E-02 | Down-regulated |
| 2337 | hsa_circ_0012421 | -2.014 | 1.40E-02 | Down-regulated |
| 2338 | hsa_circ_0066251 | -2.020 | 2.30E-02 | Down-regulated |
| 2339 | hsa_circ_0012872 | -2.021 | 1.10E-02 | Down-regulated |
| 2340 | hsa_circ_0015270 | -2.021 | 3.20E-02 | Down-regulated |
| 2341 | hsa_circ_0047736 | -2.021 | 6.00E-03 | Down-regulated |
| 2342 | hsa_circ_0002891 | -2.024 | 2.90E-02 | Down-regulated |
| 2343 | hsa_circ_0068282 | -2.025 | 1.80E-02 | Down-regulated |
| 2344 | hsa_circ_0027666 | -2.026 | 1.20E-02 | Down-regulated |
| 2345 | hsa_circ_0003137 | -2.026 | 3.30E-02 | Down-regulated |
| 2346 | hsa_circ_0007249 | -2.027 | 6.00E-03 | Down-regulated |
| 2347 | hsa_circ_0004349 | -2.027 | 1.80E-02 | Down-regulated |
| 2348 | hsa_circ_0043193 | -2.027 | 1.30E-02 | Down-regulated |
| 2349 | hsa_circ_0085096 | -2.028 | 2.30E-02 | Down-regulated |
| 2350 | hsa_circ_0005753 | -2.029 | 2.70E-02 | Down-regulated |
| 2351 | hsa_circ_0006870 | -2.033 | 2.40E-02 | Down-regulated |
| 2352 | hsa_circ_0017873 | -2.034 | 1.10E-02 | Down-regulated |
| 2353 | hsa_circ_0074146 | -2.035 | 1.20E-02 | Down-regulated |
| 2354 | hsa_circ_0005962 | -2.038 | 4.00E-02 | Down-regulated |
| 2355 | hsa_circ_0007096 | -2.042 | 6.00E-03 | Down-regulated |
| 2356 | hsa_circ_0007071 | -2.045 | 5.00E-03 | Down-regulated |
| 2357 | hsa_circ_0088603 | -2.046 | 3.30E-02 | Down-regulated |
| 2358 | hsa_circ_0035957 | -2.054 | 4.50E-02 | Down-regulated |
| 2359 | hsa_circ_0000544 | -2.054 | 1.50E-02 | Down-regulated |
| 2360 | hsa_circ_0016114 | -2.055 | 1.00E-02 | Down-regulated |
| 2361 | hsa_circ_0006095 | -2.055 | 1.50E-02 | Down-regulated |
| 2362 | hsa_circ_0004893 | -2.057 | 4.70E-02 | Down-regulated |
| 2363 | hsa_circ_0004145 | -2.059 | 1.00E-02 | Down-regulated |
| 2364 | hsa_circ_0006393 | -2.059 | 2.90E-02 | Down-regulated |
| 2365 | hsa_circ_0069227 | -2.060 | 7.00E-03 | Down-regulated |
| 2366 | hsa_circ_0008496 | -2.061 | 1.80E-02 | Down-regulated |
| 2367 | hsa_circ_0056322 | -2.063 | 2.40E-02 | Down-regulated |
| 2368 | hsa_circ_0002284 | -2.063 | 1.40E-02 | Down-regulated |
| 2369 | hsa_circ_0000083 | -2.063 | 1.30E-02 | Down-regulated |
| 2370 | hsa_circ_0074450 | -2.066 | 2.70E-02 | Down-regulated |
| 2371 | hsa_circ_0025177 | -2.068 | 1.60E-02 | Down-regulated |
| 2372 | hsa_circ_0057551 | -2.070 | 7.00E-03 | Down-regulated |
| 2373 | hsa_circ_0001873 | -2.071 | 4.20E-02 | Down-regulated |
| 2374 | hsa_circ_0020303 | -2.071 | 3.80E-02 | Down-regulated |
| 2375 | hsa_circ_0025753 | -2.072 | 6.00E-03 | Down-regulated |
| 2376 | hsa_circ_0067435 | -2.075 | 1.20E-02 | Down-regulated |
| 2377 | hsa_circ_0001538 | -2.077 | 2.30E-02 | Down-regulated |
| 2378 | hsa_circ_0039353 | -2.077 | 2.50E-02 | Down-regulated |
| 2379 | hsa_circ_0070014 | -2.079 | 1.70E-02 | Down-regulated |
| 2380 | hsa_circ_0018722 | -2.083 | 2.00E-02 | Down-regulated |
| 2381 | hsa_circ_0075303 | -2.084 | 1.70E-02 | Down-regulated |
| 2382 | hsa_circ_0001975 | -2.087 | 7.00E-03 | Down-regulated |
| 2383 | hsa_circ_0041915 | -2.090 | 2.60E-02 | Down-regulated |
| 2384 | hsa_circ_0006295 | -2.095 | 1.50E-02 | Down-regulated |
| 2385 | hsa_circ_0000458 | -2.096 | 2.00E-02 | Down-regulated |
| 2386 | hsa_circ_0006543 | -2.097 | 2.80E-02 | Down-regulated |
| 2387 | hsa_circ_0028861 | -2.098 | 1.20E-02 | Down-regulated |
| 2388 | hsa_circ_0015240 | -2.098 | 4.30E-02 | Down-regulated |
| 2389 | hsa_circ_0030741 | -2.099 | 2.50E-02 | Down-regulated |
| 2390 | hsa_circ_0091053 | -2.101 | 2.00E-03 | Down-regulated |
| 2391 | hsa_circ_0066277 | -2.104 | 5.00E-03 | Down-regulated |
| 2392 | hsa_circ_0002493 | -2.107 | 4.40E-02 | Down-regulated |
| 2393 | hsa_circ_0069837 | -2.108 | 5.00E-03 | Down-regulated |
| 2394 | hsa_circ_0030614 | -2.109 | 9.00E-03 | Down-regulated |
| 2395 | hsa_circ_0024490 | -2.109 | 2.40E-02 | Down-regulated |
| 2396 | hsa_circ_0078033 | -2.111 | 8.00E-03 | Down-regulated |
| 2397 | hsa_circ_0002793 | -2.111 | 2.30E-02 | Down-regulated |
| 2398 | hsa_circ_0021076 | -2.117 | 1.60E-02 | Down-regulated |
| 2399 | hsa_circ_0008775 | -2.117 | 6.00E-03 | Down-regulated |
| 2400 | hsa_circ_0077879 | -2.122 | 7.00E-03 | Down-regulated |
| 2401 | hsa_circ_0030541 | -2.122 | 3.10E-02 | Down-regulated |
| 2402 | hsa_circ_0027072 | -2.125 | 2.70E-02 | Down-regulated |
| 2403 | hsa_circ_0043397 | -2.126 | 6.00E-03 | Down-regulated |
| 2404 | hsa_circ_0066483 | -2.127 | 1.50E-02 | Down-regulated |
| 2405 | hsa_circ_0024758 | -2.134 | 3.60E-02 | Down-regulated |
| 2406 | hsa_circ_0090104 | -2.134 | 2.00E-02 | Down-regulated |
| 2407 | hsa_circ_0000287 | -2.135 | 3.50E-02 | Down-regulated |
| 2408 | hsa_circ_0006380 | -2.135 | 1.10E-02 | Down-regulated |
| 2409 | hsa_circ_0090122 | -2.137 | 7.00E-03 | Down-regulated |
| 2410 | hsa_circ_0057168 | -2.138 | 4.50E-02 | Down-regulated |
| 2411 | hsa_circ_0068465 | -2.139 | 8.00E-03 | Down-regulated |
| 2412 | hsa_circ_0002491 | -2.141 | 3.00E-03 | Down-regulated |
| 2413 | hsa_circ_0058792 | -2.147 | 1.60E-02 | Down-regulated |
| 2414 | hsa_circ_0059536 | -2.147 | 8.00E-03 | Down-regulated |
| 2415 | hsa_circ_0000089 | -2.147 | 2.70E-02 | Down-regulated |
| 2416 | hsa_circ_0006246 | -2.149 | 2.60E-02 | Down-regulated |
| 2417 | hsa_circ_0005576 | -2.151 | 4.90E-02 | Down-regulated |
| 2418 | hsa_circ_0020174 | -2.151 | 9.00E-03 | Down-regulated |
| 2419 | hsa_circ_0001648 | -2.153 | 2.20E-02 | Down-regulated |
| 2420 | hsa_circ_0056199 | -2.155 | 1.60E-02 | Down-regulated |
| 2421 | hsa_circ_0023569 | -2.161 | 1.00E-02 | Down-regulated |
| 2422 | hsa_circ_0007697 | -2.169 | 2.30E-02 | Down-regulated |
| 2423 | hsa_circ_0015590 | -2.169 | 1.40E-02 | Down-regulated |
| 2424 | hsa_circ_0091692 | -2.170 | 1.10E-02 | Down-regulated |
| 2425 | hsa_circ_0004851 | -2.173 | 3.20E-02 | Down-regulated |
| 2426 | hsa_circ_0085159 | -2.173 | 1.60E-02 | Down-regulated |
| 2427 | hsa_circ_0000984 | -2.176 | 8.00E-03 | Down-regulated |
| 2428 | hsa_circ_0006358 | -2.183 | 1.10E-02 | Down-regulated |
| 2429 | hsa_circ_0032030 | -2.188 | 1.10E-02 | Down-regulated |
| 2430 | hsa_circ_0072732 | -2.191 | 2.20E-02 | Down-regulated |
| 2431 | hsa_circ_0006073 | -2.194 | 1.30E-02 | Down-regulated |
| 2432 | hsa_circ_0021378 | -2.195 | 3.00E-02 | Down-regulated |
| 2433 | hsa_circ_0060134 | -2.197 | 1.20E-02 | Down-regulated |
| 2434 | hsa_circ_0034741 | -2.199 | 1.50E-02 | Down-regulated |
| 2435 | hsa_circ_0034281 | -2.205 | 3.60E-02 | Down-regulated |
| 2436 | hsa_circ_0008252 | -2.207 | 1.30E-02 | Down-regulated |
| 2437 | hsa_circ_0000259 | -2.213 | 1.90E-02 | Down-regulated |
| 2438 | hsa_circ_0057073 | -2.216 | 1.10E-02 | Down-regulated |
| 2439 | hsa_circ_0009608 | -2.220 | 1.00E-02 | Down-regulated |
| 2440 | hsa_circ_0032979 | -2.220 | 7.00E-03 | Down-regulated |
| 2441 | hsa_circ_0025341 | -2.223 | 7.00E-03 | Down-regulated |
| 2442 | hsa_circ_0023940 | -2.223 | 2.60E-02 | Down-regulated |
| 2443 | hsa_circ_0029639 | -2.224 | 3.20E-02 | Down-regulated |
| 2444 | hsa_circ_0072490 | -2.225 | 1.50E-02 | Down-regulated |
| 2445 | hsa_circ_0058794 | -2.228 | 1.30E-02 | Down-regulated |
| 2446 | hsa_circ_0002051 | -2.233 | 1.30E-02 | Down-regulated |
| 2447 | hsa_circ_0073237 | -2.238 | 3.50E-02 | Down-regulated |
| 2448 | hsa_circ_0048545 | -2.241 | 1.80E-02 | Down-regulated |
| 2449 | hsa_circ_0001062 | -2.249 | 2.10E-02 | Down-regulated |
| 2450 | hsa_circ_0058555 | -2.249 | 1.40E-02 | Down-regulated |
| 2451 | hsa_circ_0079347 | -2.260 | 1.50E-02 | Down-regulated |
| 2452 | hsa_circ_0047814 | -2.266 | 1.30E-02 | Down-regulated |
| 2453 | hsa_circ_0031859 | -2.267 | 1.00E-02 | Down-regulated |
| 2454 | hsa_circ_0027610 | -2.269 | 9.00E-03 | Down-regulated |
| 2455 | hsa_circ_0008204 | -2.271 | 2.90E-02 | Down-regulated |
| 2456 | hsa_circ_0057315 | -2.273 | 6.00E-03 | Down-regulated |
| 2457 | hsa_circ_0030431 | -2.274 | 1.80E-02 | Down-regulated |
| 2458 | hsa_circ_0080788 | -2.281 | 2.10E-02 | Down-regulated |
| 2459 | hsa_circ_0084527 | -2.289 | 3.50E-02 | Down-regulated |
| 2460 | hsa_circ_0006442 | -2.290 | 5.00E-03 | Down-regulated |
| 2461 | hsa_circ_0070047 | -2.293 | 1.30E-02 | Down-regulated |
| 2462 | hsa_circ_0001168 | -2.294 | 8.00E-03 | Down-regulated |
| 2463 | hsa_circ_0051899 | -2.297 | 1.60E-02 | Down-regulated |
| 2464 | hsa_circ_0065649 | -2.298 | 3.60E-02 | Down-regulated |
| 2465 | hsa_circ_0001968 | -2.304 | 2.40E-02 | Down-regulated |
| 2466 | hsa_circ_0001486 | -2.318 | 1.00E-02 | Down-regulated |
| 2467 | hsa_circ_0003799 | -2.318 | 1.60E-02 | Down-regulated |
| 2468 | hsa_circ_0064802 | -2.320 | 1.10E-02 | Down-regulated |
| 2469 | hsa_circ_0001527 | -2.320 | 1.10E-02 | Down-regulated |
| 2470 | hsa_circ_0014352 | -2.328 | 1.80E-02 | Down-regulated |
| 2471 | hsa_circ_0047663 | -2.333 | 8.00E-03 | Down-regulated |
| 2472 | hsa_circ_0004402 | -2.337 | 4.30E-02 | Down-regulated |
| 2473 | hsa_circ_0005392 | -2.340 | 5.00E-03 | Down-regulated |
| 2474 | hsa_circ_0009618 | -2.340 | 1.70E-02 | Down-regulated |
| 2475 | hsa_circ_0031856 | -2.345 | 1.00E-02 | Down-regulated |
| 2476 | hsa_circ_0080787 | -2.349 | 2.30E-02 | Down-regulated |
| 2477 | hsa_circ_0006105 | -2.355 | 3.80E-02 | Down-regulated |
| 2478 | hsa_circ_0007106 | -2.356 | 1.90E-02 | Down-regulated |
| 2479 | hsa_circ_0035583 | -2.360 | 4.50E-02 | Down-regulated |
| 2480 | hsa_circ_0082580 | -2.368 | 1.30E-02 | Down-regulated |
| 2481 | hsa_circ_0002397 | -2.374 | 1.60E-02 | Down-regulated |
| 2482 | hsa_circ_0032029 | -2.376 | 1.00E-02 | Down-regulated |
| 2483 | hsa_circ_0022603 | -2.378 | 2.10E-02 | Down-regulated |
| 2484 | hsa_circ_0078529 | -2.380 | 2.20E-02 | Down-regulated |
| 2485 | hsa_circ_0081943 | -2.383 | 1.50E-02 | Down-regulated |
| 2486 | hsa_circ_0006623 | -2.392 | 2.10E-02 | Down-regulated |
| 2487 | hsa_circ_0009158 | -2.396 | 3.00E-03 | Down-regulated |
| 2488 | hsa_circ_0085133 | -2.398 | 5.00E-03 | Down-regulated |
| 2489 | hsa_circ_0002300 | -2.398 | 3.90E-02 | Down-regulated |
| 2490 | hsa_circ_0075402 | -2.402 | 3.00E-03 | Down-regulated |
| 2491 | hsa_circ_0025593 | -2.402 | 7.00E-03 | Down-regulated |
| 2492 | hsa_circ_0055879 | -2.410 | 3.00E-03 | Down-regulated |
| 2493 | hsa_circ_0059963 | -2.412 | 2.20E-02 | Down-regulated |
| 2494 | hsa_circ_0070382 | -2.417 | 2.30E-02 | Down-regulated |
| 2495 | hsa_circ_0006235 | -2.431 | 4.30E-02 | Down-regulated |
| 2496 | hsa_circ_0084755 | -2.452 | 1.10E-02 | Down-regulated |
| 2497 | hsa_circ_0001542 | -2.452 | 1.60E-02 | Down-regulated |
| 2498 | hsa_circ_0059352 | -2.458 | 1.00E-02 | Down-regulated |
| 2499 | hsa_circ_0064794 | -2.475 | 1.90E-02 | Down-regulated |
| 2500 | hsa_circ_0008337 | -2.479 | 6.00E-03 | Down-regulated |
| 2501 | hsa_circ_0015298 | -2.486 | 1.00E-02 | Down-regulated |
| 2502 | hsa_circ_0057125 | -2.494 | 8.00E-03 | Down-regulated |
| 2503 | hsa_circ_0011724 | -2.498 | 3.80E-02 | Down-regulated |
| 2504 | hsa_circ_0005231 | -2.503 | 1.70E-02 | Down-regulated |
| 2505 | hsa_circ_0038383 | -2.508 | 4.00E-03 | Down-regulated |
| 2506 | hsa_circ_0000210 | -2.510 | 7.00E-03 | Down-regulated |
| 2507 | hsa_circ_0001565 | -2.527 | 1.50E-02 | Down-regulated |
| 2508 | hsa_circ_0011222 | -2.529 | 4.00E-03 | Down-regulated |
| 2509 | hsa_circ_0000123 | -2.532 | 2.00E-02 | Down-regulated |
| 2510 | hsa_circ_0075386 | -2.534 | 4.00E-03 | Down-regulated |
| 2511 | hsa_circ_0001572 | -2.543 | 1.70E-02 | Down-regulated |
| 2512 | hsa_circ_0003373 | -2.581 | 1.20E-02 | Down-regulated |
| 2513 | hsa_circ_0073239 | -2.590 | 2.90E-02 | Down-regulated |
| 2514 | hsa_circ_0032136 | -2.592 | 2.90E-02 | Down-regulated |
| 2515 | hsa_circ_0003258 | -2.593 | 2.60E-02 | Down-regulated |
| 2516 | hsa_circ_0091319 | -2.618 | 6.00E-03 | Down-regulated |
| 2517 | hsa_circ_0058192 | -2.629 | 4.00E-03 | Down-regulated |
| 2518 | hsa_circ_0036567 | -2.631 | 1.80E-02 | Down-regulated |
| 2519 | hsa_circ_0002701 | -2.635 | 9.00E-03 | Down-regulated |
| 2520 | hsa_circ_0020273 | -2.639 | 9.00E-03 | Down-regulated |
| 2521 | hsa_circ_0076989 | -2.648 | 2.80E-02 | Down-regulated |
| 2522 | hsa_circ_0023610 | -2.665 | 1.10E-02 | Down-regulated |
| 2523 | hsa_circ_0007976 | -2.669 | 3.00E-02 | Down-regulated |
| 2524 | hsa_circ_0005778 | -2.671 | 2.40E-02 | Down-regulated |
| 2525 | hsa_circ_0000372 | -2.692 | 2.60E-02 | Down-regulated |
| 2526 | hsa_circ_0051239 | -2.707 | 3.30E-02 | Down-regulated |
| 2527 | hsa_circ_0076859 | -2.708 | 1.00E-02 | Down-regulated |
| 2528 | hsa_circ_0005615 | -2.714 | 3.50E-02 | Down-regulated |
| 2529 | hsa_circ_0062051 | -2.722 | 1.30E-02 | Down-regulated |
| 2530 | hsa_circ_0033388 | -2.727 | 1.60E-02 | Down-regulated |
| 2531 | hsa_circ_0086474 | -2.739 | 9.00E-03 | Down-regulated |
| 2532 | hsa_circ_0063266 | -2.759 | 5.00E-03 | Down-regulated |
| 2533 | hsa_circ_0003570 | -2.768 | 3.30E-02 | Down-regulated |
| 2534 | hsa_circ_0000115 | -2.769 | 4.00E-03 | Down-regulated |
| 2535 | hsa_circ_0037376 | -2.824 | 2.30E-02 | Down-regulated |
| 2536 | hsa_circ_0056689 | -2.849 | 4.00E-03 | Down-regulated |
| 2537 | hsa_circ_0001510 | -2.890 | 3.70E-02 | Down-regulated |
| 2538 | hsa_circ_0001713 | -2.939 | 4.40E-02 | Down-regulated |
| 2539 | hsa_circ_0003069 | -2.961 | 1.50E-02 | Down-regulated |
| 2540 | hsa_circ_0058161 | -2.982 | 1.20E-02 | Down-regulated |
| 2541 | hsa_circ_0030162 | -3.083 | 1.90E-02 | Down-regulated |
| 2542 | hsa_circ_0027612 | -3.086 | 7.00E-03 | Down-regulated |
| 2543 | hsa_circ_0013181 | -3.106 | 1.50E-02 | Down-regulated |
| 2544 | hsa_circ_0039697 | -3.255 | 2.60E-02 | Down-regulated |
| 2545 | hsa_circ_0033392 | -3.363 | 1.60E-02 | Down-regulated |
| 2546 | hsa_circ_0085315 | -3.463 | 2.00E-03 | Down-regulated |
| 2547 | hsa_circ_0059516 | -3.560 | 4.00E-03 | Down-regulated |
| 2548 | hsa_circ_0005622 | -3.707 | 1.60E-02 | Down-regulated |


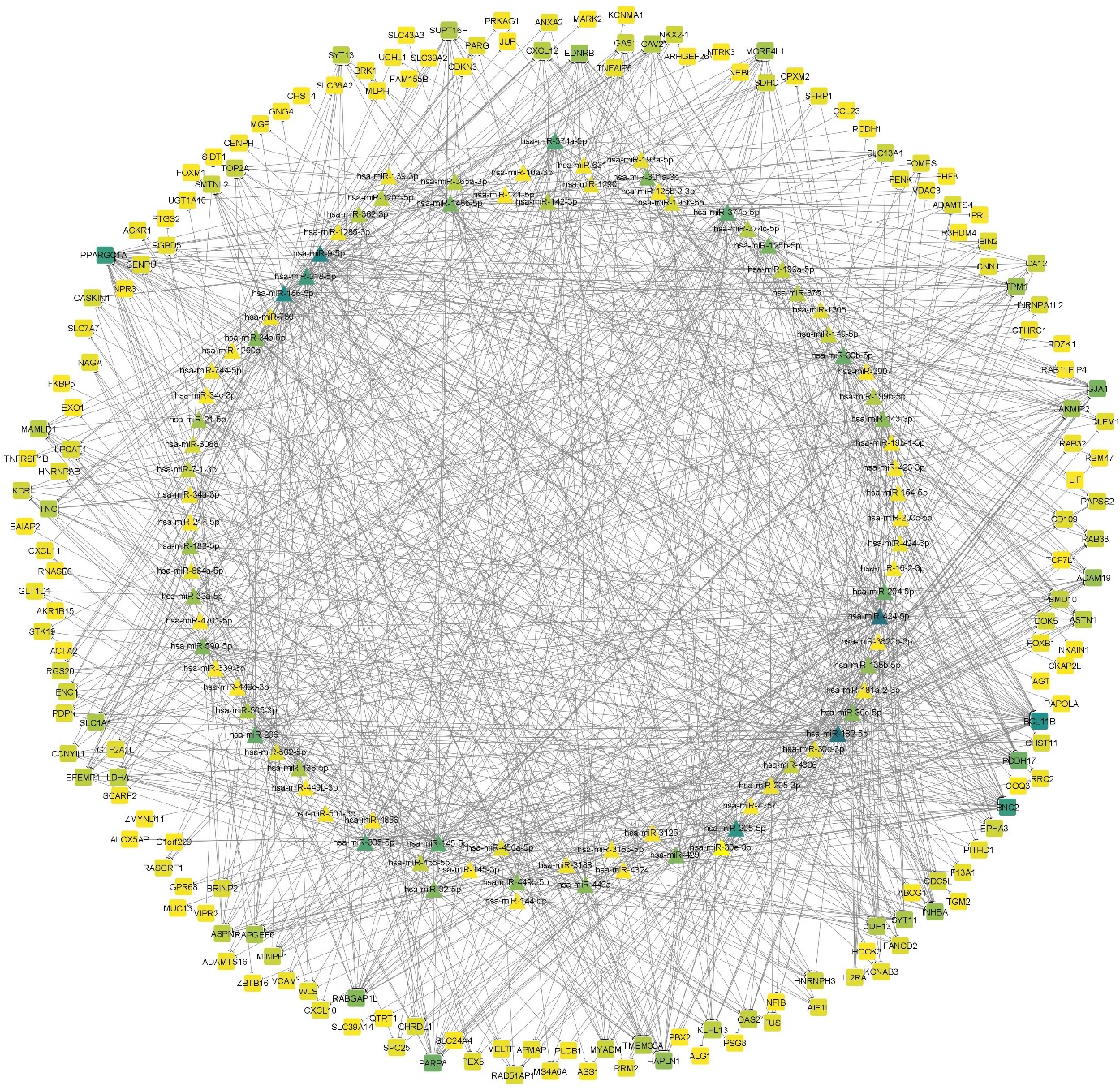


**Supplementary figure 1. The miRNA–mRNA regulatory network.** The regulatory network comprised of 257 nodes and 994 edges. The round rectangle and triangle represent mRNAs, and miRNAs respectively.


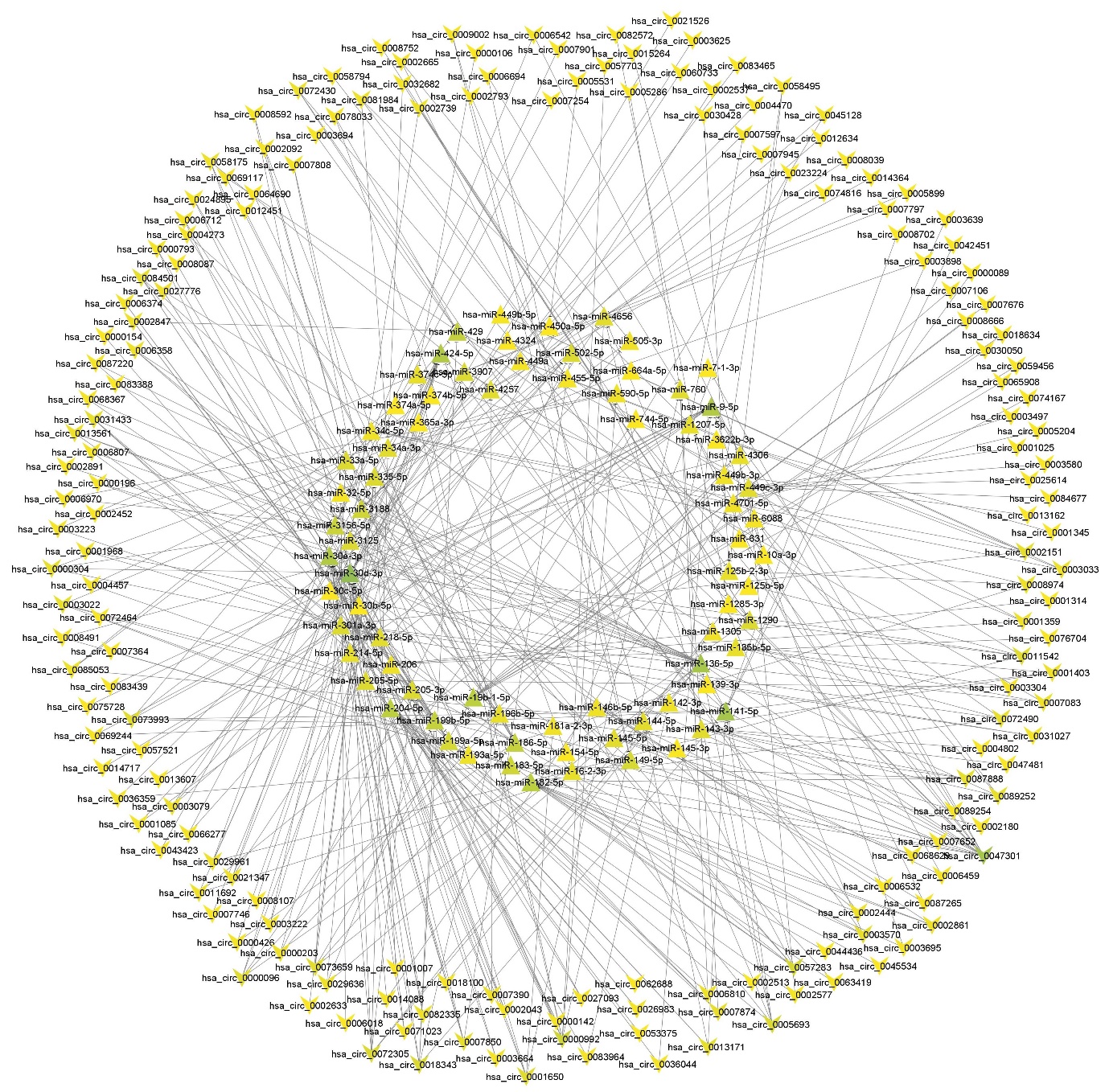


**Supplementary figure 2. The circRNA–miRNA regulatory network.** The regulatory network consisted of 257 nodes and 381 edges. The V shape and the triangle represent circRNA, and miRNAs respectively.
